# Improving the gas sorption capacity in lantern-type metal–organic polyhedra by a scrambled cage method†

**DOI:** 10.1039/d3sc06140j

**Published:** 2024-01-11

**Authors:** Beatriz Doñagueda Suso, Zaoming Wang, Alan R. Kennedy, Ashleigh J. Fletcher, Shuhei Furukawa, Gavin A. Craig

**Affiliations:** a Department of Pure and Applied Chemistry, University of Strathclyde Glasgow G1 1XL UK gavin.craig@strath.ac.uk; b Institute for Integrated Cell-Material Sciences (WPI-iCeMS), Kyoto University Yoshida, Sakyo-ku Kyoto 606-8501 Japan; c Department of Chemical and Process Engineering, University of Strathclyde Glasgow G1 1XJ UK; d Department of Synthetic Chemistry and Biological Chemistry, Graduate School of Engineering, Kyoto University Katsura, Nishikyo-ku Kyoto 615-8510 Japan

## Abstract

The synthesis of multivariate metal–organic frameworks (MOFs) is a well-known method for increasing the complexity of porous frameworks. In these materials, the structural differences of the ligands used in the synthesis are sufficiently subtle that they can each occupy the same site in the framework. However, multivariate or ligand scrambling approaches are rarely used in the synthesis of porous metal–organic polyhedra (MOPs) – the molecular equivalent of MOFs – despite the potential to retain a unique intrinsic pore from the individual cage while varying the extrinsic porosity of the material. Herein we directly synthesise scrambled cages across two families of lantern-type MOPs and find contrasting effects on their gas sorption properties. In one family, the scrambling approach sees a gradual increase in the BET surface area with the maximum and minimum uptakes associated with the two pure homoleptic cages. In the other, the scrambled materials display improved surface areas with respect to both of the original, homoleptic cages. Through analysis of the gas sorption isotherms, we attribute this effect to the balance of micro- and mesoporosity within the materials, which varies as a result of the scrambling approach. The gas uptake of the materials presented here underscores the tunability of cages that springs from their combination of intrinsic, extrinsic, micro- and meso-porosities.

## Abstract 2

Herein we use a scrambled ligand approach to tune the surface areas of metal–organic polyhedra, driven by the creation of mesoporosity.

## Graphical abstract

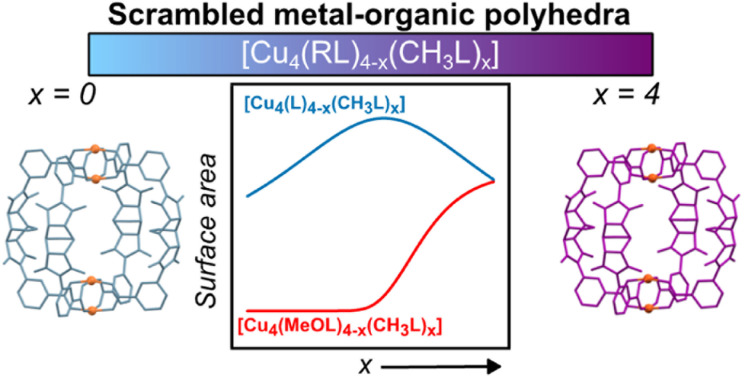

## Introduction

Metal–organic polyhedra (MOPs) or metal–organic cages (MOCs) are assembled through the coordination of organic ligands to metal nodes giving discrete molecules that contain a defined cavity.^1–5^ The shape of this cavity arises from the combination of the bonding preferences of the metal node with the geometry and denticity of the ligand(s) used in the synthesis.^6,7^ Homoleptic cages contain one type of ligand, and examples of this approach include the use of 3,3′-((1,3-phenylene)bis(ethyne-2,1-diyl))dibenzoic acid or 1,3-bis(pyridin-3-ylethynyl)benzene derivatives to synthesise lantern-type cages;^8–13^ carbazole derivatives to access octahedral cages;^14–16^ and isophthalic acid or 1,3-di(pyridine-4-yl)benzene derivatives to synthesise cuboctahedral cages.^17–23^ Alternatively, two or more types of ligand can be used, giving heteroleptic cages, increasing the complexity of the assembly as now the geometry of the cage will be determined by contributions from the bonding angles as well as the relative lengths of the ligands. In the study of self-assembly of metal–organic cages in solution, this approach is used as a way to create more complex micro-environments within the cavity of the cage itself.^24–29^ When MOPs have been investigated for gas sorption in the solid-state, heteroleptic approaches have largely involved the use of two ligands with well-defined, independent structural roles,^30–32^ such as the use of calixarenes to form bowl-type arrangements, which are then connected through isophthalates or other carboxylate-based ligands to complete the discrete cage.^33–36^

The above routes focus on the formation of the internal pore environment. However, the porosity of MOPs in the solid state also depends on the surface functionalisation of the cage,^37–39^ as this affects packing and hence, the extrinsic porosity. In this context, heteroleptic MOPs where the ligands used have the same backbone, but different external functionalisation, are less well explored as a means of tuning porosity in the cages. An early example of this type of MOP is [Cu_24_(OH-bdc)_12_(bdc)_12_], which was obtained *via* solution-phase ligand exchange on the cuboctahedral MOP [Cu_24_(OH-bdc)_24_], where OH-bdc and bdc represent 5-hydroxyisophthalate and isophthalate, respectively.^40^ In this case the effect of the ligand exchange on the gas sorption properties of the material was not reported. These processes require the MOP to present a high degree of solubility, which is the case for [Cu_24_(OH-bdc)_24_],^22,41^ and for the rate of ligand exchange to be favourable.^42,43^ Recent work from von Baeckmann and co-workers described a stepwise strategy based on protection/deprotection strategies available to more robust [Rh_2_] paddlewheels.^44^ If the MOPs are soluble, but no ligand exchange takes place, then pure homoleptic cages can also be blended to obtain alloys.^45^ When direct synthesis is employed with ligands of the same length, then the approach is similar to that found in multivariate MOFs,^46^ or analogous to the dynamic covalent scrambling that is possible for porous organic cages (POCs).^47–49^ Previous work in this area for porous MOPs is limited to a few examples.^50^ Lerma-Berlanga and co-workers used this method to develop a family of MOPs based on mononuclear Ti(iv) nodes, where the different hydrogen bond donor/acceptor capabilities of the MeO– and –NH_2_ functionalised ligands were found to affect the degree of incorporation of the ligands in the final cages.^51^ Antonio and co-workers^52^ and Nam and co-workers^53^ found that the surface areas of scrambled cages lay between those of pure homoleptic materials for [Mo_24_(bdc)_24_]-type cuboctahedra and tetrahedra based on [(Cp_3_Zr)_3_] nodes, respectively.

**Fig. 1:**
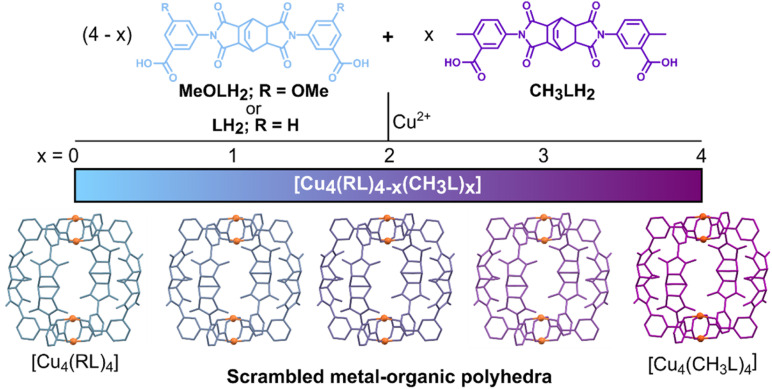
Schematic of the approach used to obtain the scrambled metal–organic polyhedra. The relevant ratios of either MeOLH_2_ or LH_2_ were mixed with CH_3_LH_2_ and reacted with Cu(ii) salts to obtain scrambled materials. The crystal structure of the material [Cu_4_(L)_2_(CH_3_L)_2_]-DMA was used to prepare the representations of the scrambled MOPs, omitting the functional groups on the cages.

Recently, we reported the gas sorption properties of a family of homoleptic lantern-type cages with the general formula [Cu_4_(RL)_4_], where RL^2−^ was a series of derivatives of the ligand LH_2_ (Fig. 1).^54^ The homoleptic lantern [Cu_4_(CH_3_L)_4_] was found to display the highest Brunauer–Emmett–Teller (BET) surface area for lantern cages, as derived from the N_2_ sorption isotherm. Herein, we target the formation of scrambled cages by using mixtures of the ligands LH_2_, CH_3_LH_2_, and MeOLH_2_ (Fig. 1) to investigate two families of MOPs: [Cu_4_(L)_4−*x*_(CH_3_L)_*x*_] and [Cu_4_(MeOL)_4−*x*_(CH_3_L)_*x*_], for *x* = 0, 1, 2, 3, and 4. Using this approach, we demonstrate that it can be used to tune the gas sorption properties in MOPs and, notably, the scrambled material [Cu_4_(L)_2_(CH_3_L)_2_] displays the highest BET surface area observed to date for lantern-type complexes, with improved gas uptake compared to both of the parent homoleptic materials.

## Results and discussion

### Synthesis of the cage families [Cu_4_(MeOL)_4−*x*_(CH_3_L)_*x*_] and [Cu_4_(L)_4−*x*_(CH_3_L)_*x*_]

**Fig. 2:**
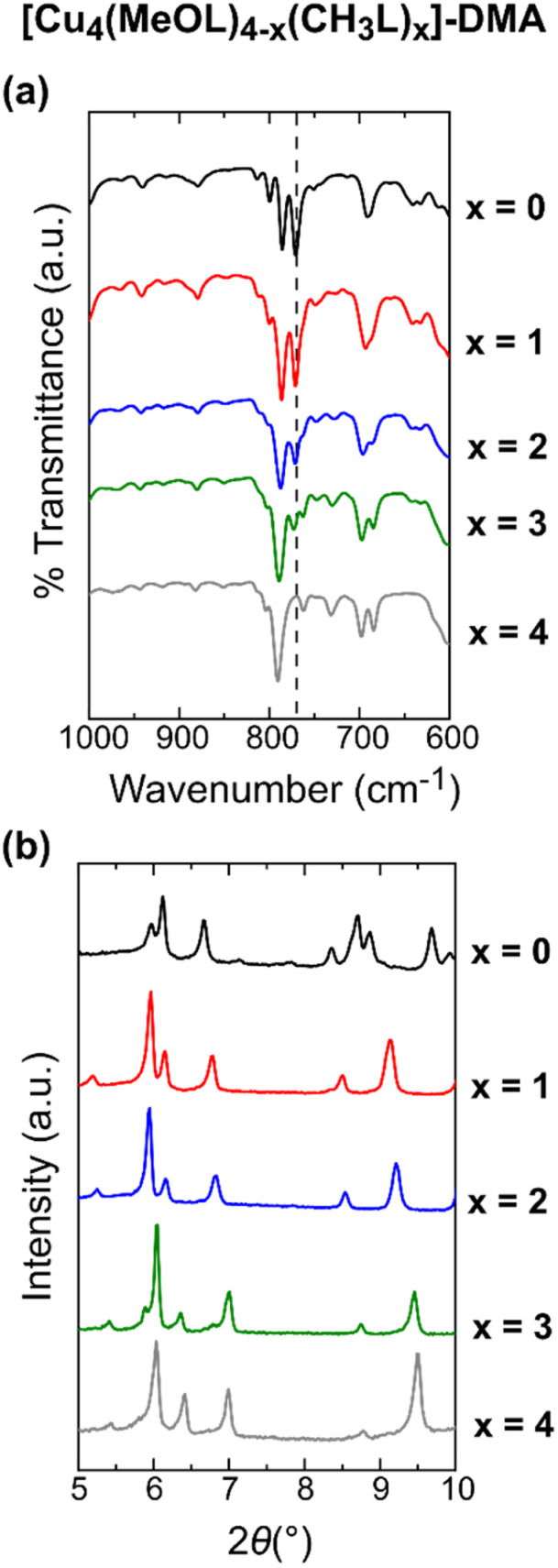
(a) IR spectra in the region 1000–600 cm^−1^ for the family of MOPs [Cu_4_(MeOL)_4−*x*_(CH_3_L)_*x*_]-DMA (*x* = 0, 1, 2, 3, 4). The dashed line highlights the decreasing intensity of a stretch at 770 cm^−1^ as *x* increases. (b) PXRD data for [Cu_4_(MeOL)_4−*x*_(CH_3_L)_*x*_]-DMA (*x* = 0, 1, 2, 3, 4), showing the isostructural nature of the crystalline powders of the scrambled cages, which all crystallise in *P*2_1_/*n*, while the homoleptic cage obtained for *x* = 0 crystallises in *C*2/*c*. The data for *x* = 0 and 4 were previously published.^54^

Although solution-based ligand exchange methods have been used to obtain heteroleptic porous coordination cages based on Cu(ii),^40,55^ the poor solubility of the as-formed homoleptic cages [Cu_4_(CH_3_L)_4_]-DMA and [Cu_4_(MeOL)_4_]-DMA precluded that approach here. Instead, a given ratio of the ligands CH_3_LH_2_ and LH_2_, or CH_3_LH_2_ and MeOLH_2_, was reacted with Cu(OAc)_2_·H_2_O in dimethylacetamide (DMA) overnight at 80 °C, yielding single crystals of the complexes. Attempts to synthesise the series [Cu_4_(MeOL)_4−_*_x_*(L)*_x_*] led to phases with poor reproducibility, and were not studied further. In addition, we synthesised a new polymorph of the cage [Cu_4_(L)_4_]-DMA, using Cu(OAc)_2_·H_2_O as the metal source (Table S1†) – this cage had previously been reported in the space group *P*2_1_/*c* when synthesised from CuCl_2_.^56^ For the cages [Cu_4_(MeOL)_4−*x*_(CH_3_L)_*x*_]-DMA (*x* = 1, 2, 3), the single crystal X-ray diffraction data were of sufficient quality to extract the unit cell parameters (Table S2†) and show the connectivity of the core of the lantern (Fig. S1†), but were too highly disordered to accurately model the positions of the methoxy- and methyl substituents of the cages, in common with other attempts to obtain scrambled metal–organic polyhedra.^52^ These scrambled cages crystallise in the monoclinic space group *P*2_1_/*n*, as does the homoleptic cage [Cu_4_(CH_3_L)_4_]-DMA, but differing from the cage [Cu_4_(MeOL)_4_]-DMA (*C*2/*c*). All of the cages are neutral, formed by two [Cu_2_] paddlewheel nodes linked through four deprotonated organic linkers. As the crystallographic data were too disordered to resolve the positions of the methyl and methoxy-groups, IR spectroscopy was used to track the scrambling of the cages, illustrated by the decrease in the intensity of the stretch at 770 cm^−1^ as the content of the ligand MeOL^2−^ decreases (Fig. 2(a) and S5†). Comparison of the powder X-ray diffraction patterns show that the scrambled cages display similar packing (Fig. 2(b) and S6†). To prove that the scrambled cages were not a mixture of crystallites, a physical mixture of the homoleptic cages [Cu_4_(CH_3_L)_4_]-DMA and [Cu_4_(MeOL)_4_]-DMA was prepared for comparison with the scrambled sample [Cu_4_(MeOL)_2_(CH_3_L)_2_]-DMA. The physical mixture was formed by grinding together separately synthesised samples of the homoleptic cages until formation of a homogenous powder [Cu_4_(MeOL)_4_]-DMA + [Cu_4_(CH_3_L)_4_]-DMA, with this powder showing features from the separate diffractograms corresponding to the unique cages (Fig. S8–S9†), distinct to the diffractogram for [Cu_4_(MeOL)_2_(CH_3_L)_2_]-DMA. From ^1^H-NMR digestion experiments, the connectivity observed in the SXRD data, and thermogravimetric analysis (Fig. S10–S16†), the bulk, averaged composition of the scrambled cages is proposed to be [Cu_4_(MeOL)_3_(CH_3_L)(DMA)_2_(H_2_O)_2_]·7DMA·2H_2_O, [Cu_4_(MeOL)_2_(CH_3_L)_2_(DMA)_2_(H_2_O)_2_]·6DMA·2H_2_O, and [Cu_4_(MeOL)(CH_3_L)_3_(DMA)_2_(H_2_O)_2_]·7DMA·H_2_O, for the cages [Cu_4_(MeOL)_3_(CH_3_L)]-DMA, [Cu_4_(MeOL)_2_(CH_3_L)_2_]-DMA, and [Cu_4_(MeOL)(CH_3_L)_3_]-DMA, respectively.

**Fig. 3:**
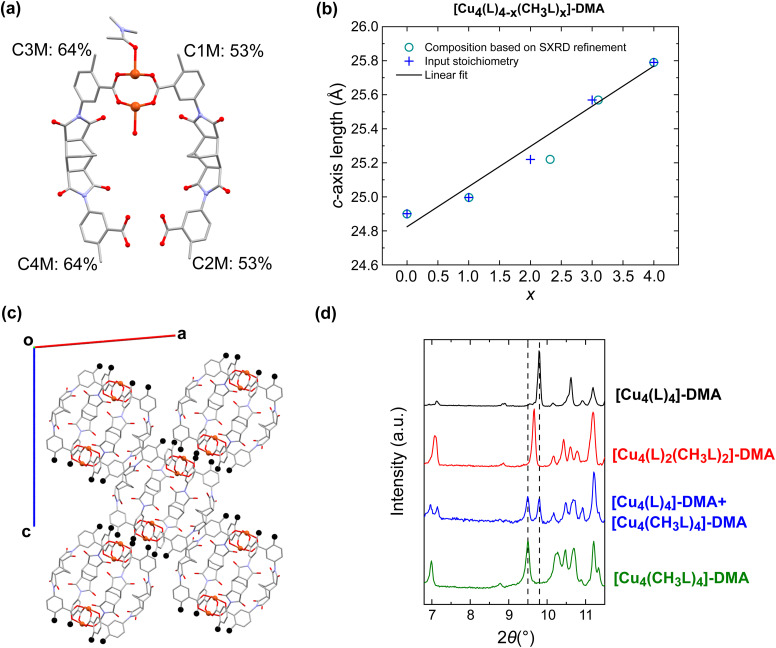
(a) View of the asymmetric unit for the isostructural cages [Cu_4_(L)_4−*x*_(CH_3_L)_*x*_]-DMA. The percentages shown represent the occupancy of the methyl functional groups in the cage for the case *x* = 2. Hydrogen atoms and all non-coordinated solvent molecules have been omitted for clarity. (b) Plot of the dependence of the length of the *c*-axis of the unit cell on the content of CH_3_L^2−^ in the cages. The composition of the cages derived from refinement of the SXRD data is shown together with the input stoichiometry of the ligand, and the line represents a straight line fit. (c) View along the *b*-axis of the cage [Cu_4_(L)_2_(CH_3_L)_2_]-DMA, with the methyl groups shown as black spheres to emphasise that they point along the *c*-axis. (d) Comparison of the powder X-ray diffraction data for the homoleptic cages, the scrambled cage [Cu_4_(L)_2_(CH_3_L)_2_]-DMA, and the physical mixture [Cu_4_(L)_4_]-DMA + [Cu_4_(CH_3_L)_4_]-DMA. The dashed lines correspond to 2*θ* = 9.50 and 9.80°.

The second family of scrambled cages based on varying ratios of CH_3_LH_2_ and LH_2_ were suitable for single-crystal X-ray analysis. Across the series [Cu_4_(L)_4−*x*_(CH_3_L)_*x*_]-DMA (*x* = 0, 1, 2, 3, and 4), all of the cages crystallise in the monoclinic space group *P*2_1_/*n*. DMA molecules coordinate to the outer sites of the paddlewheel while water molecules are coordinated in the inner site of the paddlewheel (Fig. S18 and S19†). The asymmetric unit of each member of the family consists of one half of a cage, with two crystallographically independent positions for the ligands (Fig. 3(a)). Therefore, rather than occupying a unique position within the MOP, yielding an ordered heteroleptic cage of unique composition, the ligand CH_3_L^2−^ is split over both possible sites. It was possible to refine these occupancies, as shown in Fig. 3 for the cage [Cu_4_(L)_2_(CH_3_L)_2_]-DMA, where the occupancy of one site by CH_3_L^2−^ is 64% and in the other it is 53%. Because of the previously mentioned poor solubility of the cages, solution-phase analysis techniques such as mass spectrometry were not possible to evaluate the relative populations of the distinct possible cage compositions. Based on the occupancies determined from the crystallographic data, the most probable composition of a cage in the material [Cu_4_(L)_2_(CH_3_L)_2_]-DMA is [Cu_4_(L)_2_(CH_3_L)_2_] (35.7%), while the next most probable composition is [Cu_4_(L)(CH_3_L)_3_] (33.3%). Similarly, for [Cu_4_(L)_3_(CH_3_L)]-DMA it is [Cu_4_(L)_3_(CH_3_L)] (42.6%), and for [Cu_4_(L)(CH_3_L)_3_]-DMA it is [Cu_4_(L)(CH_3_L)_3_] (43.8%). For all of the scrambled MOPs there is a significant presence of the other possible compositions in the crystal structure, but the statistically most likely composition matches the composition that was targeted (Fig. S20†). The average composition of the cages as determined by NMR digestion experiments is close to that determined from the single crystal diffraction data (Fig. S32–S34†), with the discrepancies attributed to the uncertainty arising from the disorder in the structure. As the content of the ligand CH_3_L^2−^ increases within the material, a smooth increase in the length of the *c*-axis of the *P*2_1_/*n* unit cell is observed, from 24.9013(2) to 25.7952(1) Å for the homoleptic cages [Cu_4_(L)_4_]-DMA and [Cu_4_(CH_3_L)_4_]-DMA at either end of the series, respectively. This behaviour fits with Vegard's law, as has been observed previously for alloys of porous organic cages, and in keeping with the solid solution behaviour of the samples (Fig. 3(b)).^57^ This trend is due to the crystal packing of the molecules, in which the methyl groups of the ligand CH_3_L^2−^ point approximately along the *c*-axis, causing the increase in the cell dimension as the occupancy increases (Fig. 3(c)). To confirm the solid-solution like properties of the as-synthesised scrambled cages, again a physical mixture of the cages [Cu_4_(CH_3_L)_4_]-DMA and [Cu_4_(L)_4_]-DMA was made, with the resulting sample labelled as [Cu_4_(L)_4_]-DMA + [Cu_4_(CH_3_L)_4_]-DMA. Powder X-ray diffraction of the physical mixture displays two, well defined peaks arising from the [1 1 −2] planes of each homoleptic MOP, while the diffractogram obtained for [Cu_4_(L)_2_(CH_3_L)_2_]-DMA, shows one unique peak at 2*θ* = 9.65°, confirming the unique phases obtained through the direct synthesis of the scrambled cages (Fig. 3(d) and S37†).

### Solvent-exchanged phases

**Fig. 4:**
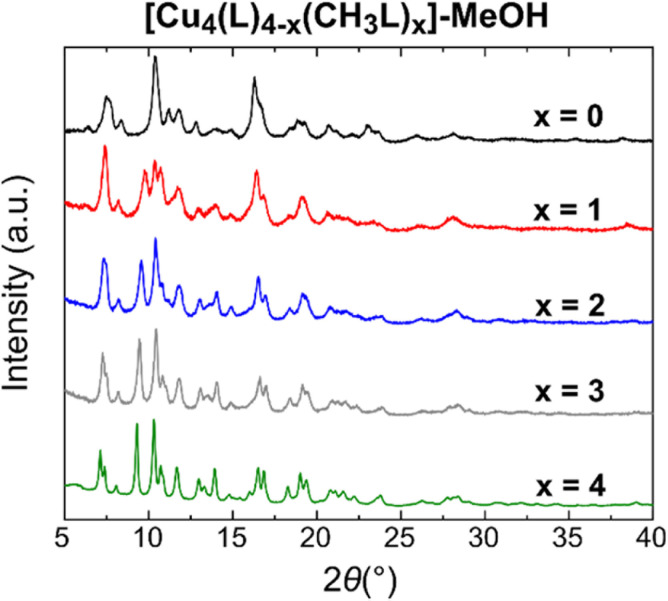
PXRD data showing the increase in crystallinity of the MeOH-exchanged cages [Cu_4_(L)_4−*x*_(CH_3_L)_*x*_]-MeOH as the content of the CH_3_L^2−^ ligand increases.

The scrambled cages were soaked in MeOH to exchange out the DMA molecules prior to activation and gas sorption measurements. All samples were characterised *via* IR spectroscopy, PXRD, TGA, and ^1^H-NMR spectroscopy of digested samples, with the data presented in the ESI.† IR spectra of the samples (Fig. S39 and S40†) show that the characteristic stretching vibration highlighted in Fig. 2(a) is still present in all of the corresponding cages with its relative intensity unchanged, suggesting that the composition of the cages is unaltered after solvent exchange. The ^1^H-NMR spectra (Fig. S41–S47†) of acid-digested samples confirm that the composition of the cages in terms of the ligand ratios remains the same, with successful exchange of DMA for MeOH taking place, while the TGA data show that the overall thermal stability of the cages is retained. The PXRD data for [Cu_4_(L)_4−*x*_(CH_3_L)_*x*_]-MeOH show that as *x* increases, the crystallinity of the bulk powder also increases (Fig. 4). A similar, although less pronounced, effect is also observed for the family [Cu_4_(MeOL)_4−*x*_(CH_3_L)_*x*_]-MeOH (Fig. S48†), although the overall effect of the methoxy substituent appears to be a reduction in the crystallinity of this family of cages compared to the family [Cu_4_(L)_4−*x*_(CH_3_L)_*x*_].

### Gas sorption properties

**Fig. 5:**
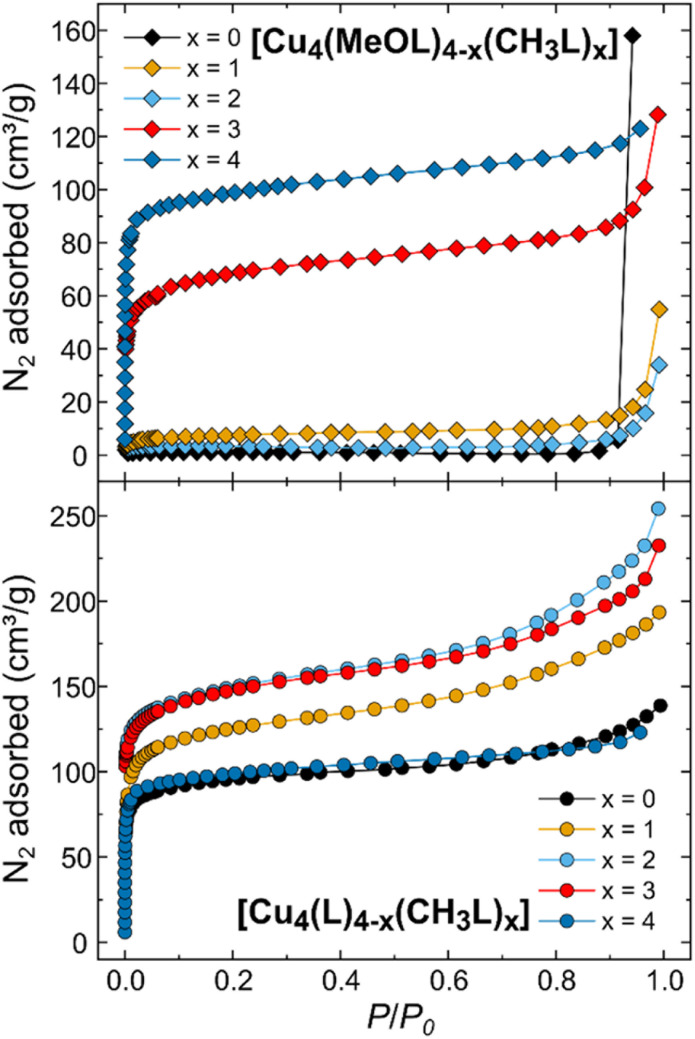
(Top) N_2_ uptake at 77 K for the family [Cu_4_(MeOL)_4−*x*_(CH_3_L)_*x*_]. The data for *x* = 0 and 4 were previously published, and are provided here for comparison. (Bottom) N_2_ uptake measured at 77 K for the family [Cu_4_(L)_4−*x*_(CH_3_L)_*x*_]. The data for *x* = 4 were previously published and are provided here for comparison.^54^ For clarity, only the adsorption branches are shown, and desorption data are given in the ESI.†

**Fig. 6:**
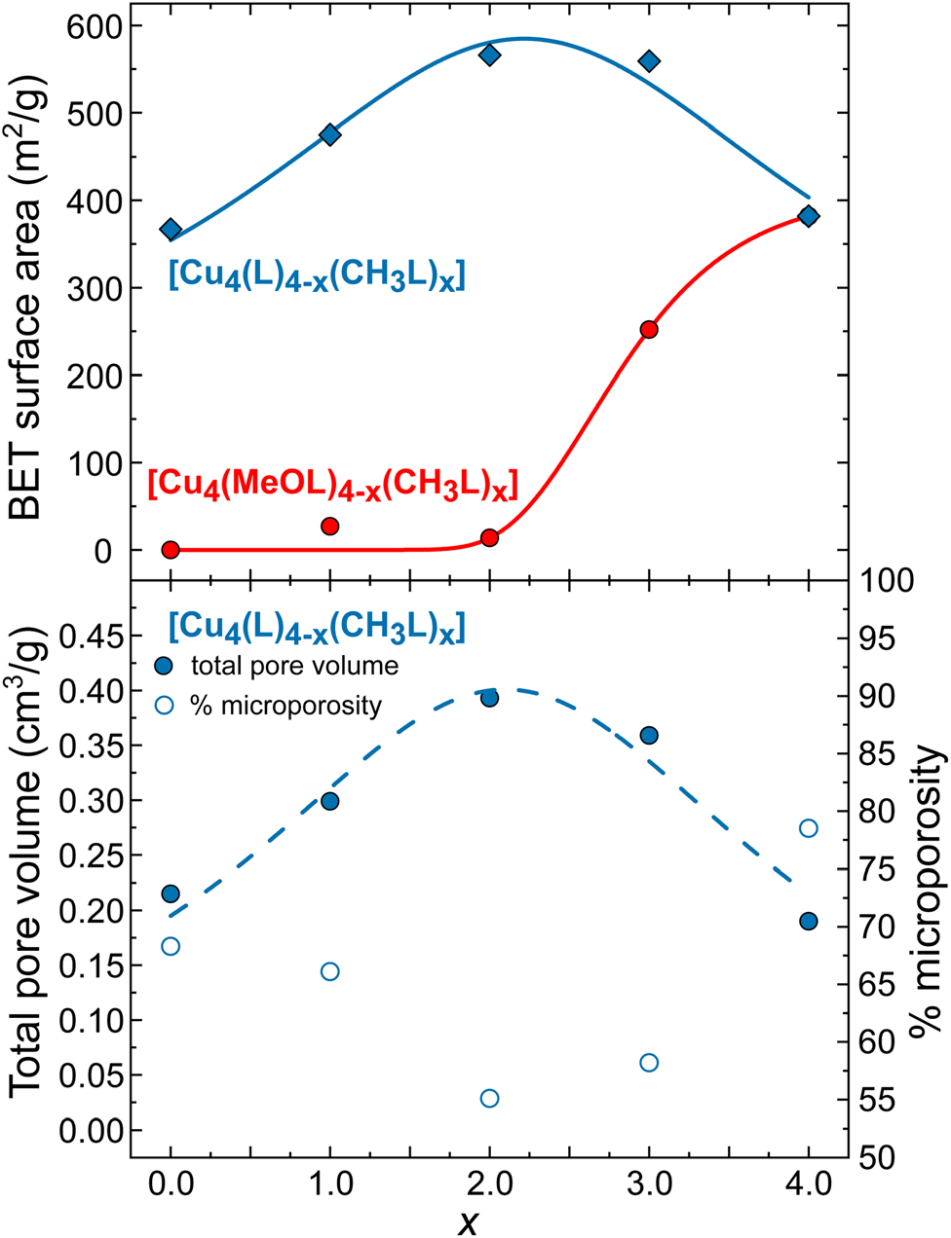
(Top) Calculated BET surface areas for the families of cages [Cu_4_(MeOL)_4−*x*_(CH_3_L)_*x*_] (filled red symbols) and [Cu_4_(L)_4−*x*_(CH_3_L)_*x*_] (filled blue symbols). The red line and blue lines are guides for the eye derived from sigmoidal and Lorentzian fits, respectively. (Bottom) Plot of the total pore volume (filled symbols) and % microporosity (emtpy symbols) for the cages [Cu_4_(L)_4−*x*_(CH_3_L)_*x*_]. The dashed line is a guide for the eye based on a Lorentzian fit of the data.

To assess the effect of scrambling on the gas sorption properties of the cages, the uptake of N_2_ at 77 K and CO_2_ at 195 K was measured. We have also measured the previously unreported gas sorption properties for the non-functionalised, homoleptic cage [Cu_4_(L)_4_]. Previously, we reported the gas sorption isotherms for the homoleptic cages [Cu_4_(MeOL)_4_] and [Cu_4_(CH_3_L)_4_], and those data are included here for comparison. Fig. 5 displays the N_2_ adsorption isotherms for both families of cages measured at 77 K (the desorption data, and isotherms for CO_2_ are provided in the ESI, Fig. S54–S64†). While the homoleptic cage [Cu_4_(MeOL)_4_] does not present N_2_ uptake at 77 K, [Cu_4_(CH_3_L)_4_] displays the steep uptake associated with microporous materials, and BETSI was used to determine a BET surface area of 380 m^2^ g^−1^.^58^ Here, all of the scrambled materials present Type H4 isotherms.^59^ As *x* increases for [Cu_4_(MeOL)_4−*x*_(CH_3_L)_*x*_], the uptake of N_2_ is shown to improve significantly for *x* ≥ 3. For [Cu_4_(MeOL)(CH_3_L)_3_], the uptake at *P*/*P*_0_ ≈ 0.9 is of 85.8 cm^3^ g^−1^, compared to 5.8 cm^3^ g^−1^ for [Cu_4_(MeOL)_2_(CH_3_L)_2_], and 117.4 cm^3^ g^−1^ for [Cu_4_(CH_3_L)_4_]. This is reflected by an increase in the BET surface areas as *x* increases (Fig. 6), reaching a maximum for the homoleptic material [Cu_4_(CH_3_L)_4_]. This trend, in which mixed cages show gas uptake that lies between that found for pure homoleptic cages has been observed for tetrahedra based on [Cp_3_Zr_3_] nodes and cuboctahedra containing [Mo_2_] paddlewheels linked with functionalised isophthalate ligands.^52,53^ In addition, we measured the N_2_ uptake for the physical mixture [Cu_4_(MeOL)_4_] + [Cu_4_(CH_3_L)_4_], and found that it displayed superior gas uptake to the scrambled material [Cu_4_(MeOL)_2_(CH_3_L)_2_] (Fig. S60†). We propose that this is due to the presence in the physical mixture of the pure homoleptic cage [Cu_4_(CH_3_L)_4_], which may not be present to a large extent in the scrambled material [Cu_4_(MeOL)_2_(CH_3_L)_2_]. The uptake of CO_2_ at 195 K, of all of the members of the family [Cu_4_(MeOL)_4−*x*_(CH_3_L)_*x*_], is found to be Type I, with a range from 109.9 cm^3^ g^−1^ to 127.7 cm^3^ g^−1^ at *P*/*P*_0_ ≈ 0.9 for [Cu_4_(CH_3_L)_4_] and [Cu_4_(MeOL)_3_(CH_3_L)], respectively. In this case, the scrambled materials do not greatly improve the CO_2_ sorption capacity with respect to their homoleptic analogues (Fig. S64†). Post-sorption analysis of the scrambled cages [Cu_4_(MeOL)_3_(CH_3_L)], [Cu_4_(MeOL)_2_(CH_3_L)_2_], and [Cu_4_(MeOL)(CH_3_L)_3_] using IR spectroscopy and PXRD suggest that the samples retain the same packing as their MeOH solvates, indicating that the samples do not decompose after activation (Fig. S65–S70†). The ^1^H-NMR spectra of digested samples show that the scrambled cage [Cu_4_(MeOL)_3_(CH_3_L)] retains 0.06 MeOH/cage (Fig. S71†), which we attribute to MeOH coordinated to the paddlewheel. Previously, we had observed that subsequent to activation the homoleptic cage [Cu_4_(MeOL)_4_] also retained MeOH, but to a greater degree, with a solvent content of 0.4 molecules of MeOH per molecule of cage.

Next, we studied the effect of scrambling on the gas sorption properties for the family [Cu_4_(L)_4−*x*_(CH_3_L)_*x*_]. In this case both homoleptic cages show N_2_ uptake, although the sorption properties of the cage [Cu_4_(L)_4_] had not previously been reported. This homoleptic cage presents a Type I isotherm with rapid uptake of N_2_ at 77 K, reaching 88.8 cm^3^ g^−1^ at *P*/*P*_0_ ≈ 0.06. At *P*/*P*_0_ ≈ 0.92, the uptake was of 123.7 cm^3^ g^−1^. The BET surface area calculated for [Cu_4_(L)_4_] is 367 m^2^ g^−1^ (Fig. S74 and S75†), compared to the previously reported BET surface area of 382 m^2^ g^−1^ for [Cu_4_(CH_3_L)_4_]. For the scrambled cages [Cu_4_(L)_3_(CH_3_L)], [Cu_4_(L)_2_(CH_3_L)_2_], and [Cu_4_(L)(CH_3_L)_3_], all show increased uptake of N_2_ in the low pressure region *P*/*P*_0_ < 0.1 when compared to the homoleptic cages. Similarly, all show higher uptake at *P*/*P*_0_ ≈ 0.9, of 172.6, 217.2, and 201 cm^3^ g^−1^ for [Cu_4_(L)_3_(CH_3_L)], [Cu_4_(L)_2_(CH_3_L)_2_], and [Cu_4_(L)(CH_3_L)_3_], respectively. Comparable increases in the adsorption of CO_2_ at 195 K are also found for the scrambled materials. This behaviour is reflected in higher BET surface areas for all of the scrambled cages when compared to the homoleptic materials, of 475, 566, and 559 m^2^ g^−1^ for [Cu_4_(L)_3_(CH_3_L)], [Cu_4_(L)_2_(CH_3_L)_2_], and [Cu_4_(L)(CH_3_L)_3_], respectively, as determined from the N_2_ adsorption isotherms (Fig. 6 and S76–S81†). This trend is rare in metal–organic cages, and we could only identify one other example where scrambling the cages in this way led to higher surface areas than for the respective homoleptic cages, reported by Lerma-Berlanga and co-workers.^51^ In contrast to the family [Cu_4_(MeOL)_4−*x*_(CH_3_L)_*x*_], post-sorption analysis of the cages [Cu_4_(L)_4−*x*_(CH_3_L)_*x*_] (*x* = 0, 1, 2, and 3) showed that all of the cages were fully activated, with no remaining MeOH in the ^1^H-NMR spectra collected subsequent to digestion (Fig. S87–S90†). The remaining post-sorption analysis of the samples *via* IR spectroscopy and PXRD (Fig. S91–S98†) confirm that the materials retain the packing of the MeOH solvated phases upon completion of gas sorption measurements.

To understand the origin of the effect of scrambling on the gas sorption properties of both families of cages, we undertook Dubinin–Radushkevich analysis as an approximation to evaluate the relative contributions from micro- and mesoporosity in the cages. This analysis can be used to calculate the micropore volume within a sample, which is derived from the intercept of the Dubinin–Radushkevich plot with the *y*-axis, log(*V*), where *V* is the adsorbed volume of N_2_ (plots are provided alongside the corresponding N_2_ isotherm in the ESI†).^60,61^ This was then compared with the total pore volumes of the solids, which were obtained from the adsorbed volume of N_2_ observed at higher values of *P*/*P*_0_. Fig. 6 presents this analysis for the family of cages [Cu_4_(L)_4−*x*_(CH_3_L)_*x*_]. The scrambled cages display higher total pore volumes than the respective homoleptic materials. At the same time, the relative contribution to this volume from microporosity decreases: for the scrambled material [Cu_4_(L)_2_(CH_3_L)_2_] this is approximately 55%, compared to 78% for the cage [Cu_4_(CH_3_L)_4_]. Therefore, we suggest that the most important effect of the scrambling process is the creation of mesopores, arising from inefficient packing of the cages.

## Conclusions

In this paper, we have used scrambled cage approaches to tune the gas sorption properties of two families of lantern-type MOPs. While we were able to crystallise and refine the crystal structures of all of the homoleptic cages, the disorder induced by the scrambling in the family [Cu_4_(MeOL)_4−*x*_(CH_3_L)_*x*_] (*x* = 1, 2, 3) meant that it was not possible to determine the exact positions of the functional groups on the surface of the cages, which we attribute to the bulky nature of the substituents causing a greater degree of disorder at the periphery of the MOPs. The effect of scrambling was to gradually increase the surface area observed for the cages as *x* increased, although the scrambled cages showed lower uptake of N_2_ than the homoleptic cage [Cu_4_(CH_3_L)_4_]. For the family [Cu_4_(L)_4−*x*_(CH_3_L)_*x*_], it was found that the ligands scrambled across the entire cage, rather than occupying one specific position of the four possible positions in the lantern cage. The increased incorporation of the bulkier methyl group to the structure led to a smooth increase in the unit cell parameter *c*, in line with solid solutions. In this case, the scrambled cages showed enhanced surface areas with respect to either of the homoleptic cages [Cu_4_(CH_3_L)_4_] or [Cu_4_(L)_4_]. The scrambled material [Cu_4_(L)_2_(CH_3_L)_2_] shows the highest BET surface area determined so far for lantern-type cages. The application of Dubinin–Radushkevich analysis suggests that scrambling leads to materials with a relatively greater contribution to the total pore volume from mesopores than is found for the pure homoleptic cages. Controlling mesoporosity and the extrinsic porosity of these cages remains a challenge. The contrasting effects on the gas sorption properties of the cages presented here highlight that scrambling approaches are a worthwhile method for MOP synthesis, as they can allow gas uptake to be tuned while retaining a unique pore geometry. This effect could also be studied for scrambling metal sites on the paddlewheels of MOPs, as a means of varying the availability and chemical nature of open metal sites in the activated materials.

## Experimental

### Synthesis

The ligands LH_2_, CH_3_LH_2_, and MeOLH_2_ were synthesised following previously reported procedures.^54,56^ The solvent content provided is based on the analysis of TGA data and NMR digestions for [Cu_4_(MeOL)_4−*x*_(CH_3_L)_*x*_] (*x* = 1, 2, 3) as the solvent could not be refined in the diffraction data; and on the single-crystal X-ray diffraction data for [Cu_4_(L)_4−*x*_(CH_3_L)_*x*_] (*x* = 0, 1, 2, 3), where solvent was refined.

[Cu_4_(L)_4_(DMA)_2_(H_2_O)_2_]·11DMA·3H_2_O.([Cu_4_(L)_4_]-DMA). A solution of LH_2_ (144 mg, 0.30 mmol) in DMA (4 mL) was combined with a solution of Cu(OAc)_2_·H_2_O (58 mg, 0.29 mmol) in DMA (4 mL). The resulting blue solution was placed in an oven at 80 °C overnight, giving 234 mg of blue crystals. 158 mg of these crystals were exchanged with fresh MeOH twice per day for 4 days, affording 114 mg of [Cu_4_(L)_4_]·5 MeOH as a blue crystalline powder.

The procedure for the synthesis of the scrambled cages was the same in each case, as described in detail here for [Cu_4_(MeOL)_3_(CH_3_L)]-DMA: [Cu_4_(MeOL)_3_(CH_3_L)(DMA)_2_(H_2_O)_2_]·7DMA·2H_2_O. ([Cu_4_(MeOL)_3_(CH_3_L)]-DMA). A solution containing CH_3_LH_2_ (75 mg, 0.15 mmol) and MeOLH_2_ (240 mg, 0.44 mmol) in DMA (8 mL), was mixed with a solution containing Cu(OAc)_2_·H_2_O (116 mg, 0.58 mmol) in DMA (8 mL). The resulting blue solution was left in the oven at 80 °C overnight giving blue/green crystals that were suitable for single crystal X-ray diffraction. Yield = 478 mg. 304 mg of these crystals were solvent exchanged with fresh MeOH twice per day for 4 days giving 240 mg of [Cu_4_(MeOL)_3_(CH_3_L)]·5MeOH as a blue powder. IR spectra, TGA data, PXRD data, and ^1^H-NMR spectra of digested samples are provided in the ESI.†

[Cu_4_(MeOL)_2_(CH_3_L)_2_(DMA)_2_(H_2_O)_2_]·6DMA·2H_2_O. ([Cu_4_(MeOL)_2_(CH_3_L)_2_]-DMA). CH_3_LH_2_ (148 mg, 0.29 mmol); MeOLH_2_ (159 mg, 0.29 mmol); Cu(OAc)_2_·H_2_O (120 mg, 0.60 mmol). Yield = 428 mg. 310 mg of these crystals were solvent exchanged with MeOH giving 249 mg of [Cu_4_(MeOL)_2_(CH_3_L)_2_]·4 MeOH as a blue powder.

[Cu_4_(MeOL)(CH_3_L)_3_(DMA)_2_(H_2_O)_2_]·7DMA·1H_2_O. ([Cu_4_(MeOL)(CH_3_L)_3_]-DMA). CH_3_LH_2_ (225 mg, 0.44 mmol); MeOLH_2_ (81 mg, 0.15 mmol); Cu(OAc)_2_·H_2_O (118 mg, 0.59 mmol). Yield = 471 mg. 306 mg of these crystals were solvent exchanged with MeOH giving 240 mg of [Cu_4_(MeOL)(CH_3_L)_3_]·5MeOH as blue powder.

[Cu_4_(L)_3_(CH_3_L)(DMA)_2_(H_2_O)_2_]·8.7DMA·1.9H_2_O. ([Cu_4_(L)_3_(CH_3_L)]-DMA). CH_3_LH_2_ (37 mg, 0.07 mmol); LH_2_ (106 mg, 0.22 mmol); Cu(OAc)_2_·H_2_O (60 mg, 0.30 mmol); DMA (8 mL). Yield = 280 mg. 151 mg of these crystals were solvent exchanged with MeOH giving 117 mg of [Cu_4_(L)_3_(CH_3_L)]·5 MeOH as a blue powder.

[Cu_4_(L)_2_(CH_3_L)_2_(DMA)_2_(H_2_O)_2_]·8.6DMA·1.9H_2_O. ([Cu_4_(L)_2_(CH_3_L)_2_]-DMA). CH_3_LH_2_ (74 mg, 0.14 mmol); LH_2_ (70 mg, 0.14 mmol); Cu(OAc)_2_·H_2_O (59 mg, 0.30 mmol); DMA (8 mL). Yield = 222 mg. 143 mg of these crystals were solvent exchanged with MeOH giving 108 mg of [Cu_4_(L)_2_(CH_3_L)_2_]·5 MeOH as a blue powder.

[Cu_4_(L)(CH_3_L)_3_(DMA)_2_(H_2_O)_2_]·5.2DMA·3.5H_2_O. ([Cu_4_(L)(CH_3_L)_3_]-DMA). CH_3_LH_2_ (111 mg, 0.22 mmol); LH_2_ (35 mg, 0.07 mmol); Cu(OAc)_2_·H_2_O (60 mg, 0.30 mmol); DMA (8 mL). Yield = 225 mg. 149 mg of these crystals were solvent exchanged with MeOH giving 114 mg of [Cu_4_(L)(CH_3_L)_3_]·6 MeOH as a blue powder.

### Physical characterisation

Infra-red spectra were collected using a Thermo Scientific spectrometer model NICOLET iS5 using 64 scans and a resolution of 4 cm^−1^. ^1^H-NMR spectra were measured with a Bruker AVANCE 400 NMR spectrometer at 25 °C operating at 400.13 MHz for ^1^H. For acid digestion of the complexes, *ca.* 15 mg of the complex were suspended in DMSO-d_6_ and 40 μL of DCl solution was added. The mixture was left to stand for 3 h at RT, resulting in a yellow solution suitable for NMR measurements. Thermogravimetric analyses (TGA) were performed with a NETZSCH STA 449 F1 Jupiter under N_2_ using an isotherm for 10 min at 30 °C before heating up to 500 °C at a rate of 10 °C min^−1^. Powder X-ray diffraction (PXRD) data were collected in a flat plate configuration using a Bruker D8 Discover diffractometer equipped with Cu K_α_ source (*λ* = 1.54056 Å). SEM samples were prepared by placing a small amount of sample on a conductive carbon adhesive label attached to a metal tack, which was sputter coated using a palladium disc connected to a Polar SC500A coater. Coating was carried out for four minutes at a current density of 20 mA under an argon atmosphere. SEM images were taking on a JEOL JSM-IT100 with a Tungsten source and operating at 5 kV and 40 mA current. For gas sorption measurements, all samples were activated *in situ* by heating at 413 K for 10 hours under vacuum before measuring their N_2_ uptake at 77 K using a Micrometrics ASAP 2420 (University of Strathclyde) and CO_2_ (195 K) using a BELSORP-miniX volumetric adsorption instrument from BEL Japan, Inc (iCeMS, Kyoto University).

### Single crystal X-ray diffraction

All data was collected using a Rigaku model XtaLAB Synergy diffractometer equipped with a Hybrid Pixel Array Detector and Cu Kα radiation (*λ* = 1.54184 Å). Structures were solved with the SHELXT solution program using intrinsic phasing, and refined with ShelXL^62^ using least squares minimisation (Tables S1–S3†), within the program Olex 2-1.5.^63^ Due to high levels of disorder for the family [Cu_4_(MeOL)_4−*x*_(CH_3_L)_*x*_] (*x* = 1, 2, 3), only the unit cell parameters are given. Full details of structural refinements are provided in the ESI.† The .cifs have been deposited with the Cambridge Crystallographic Data Centre (CSD, https://www.ccdc.cam.ac.uk/) and have the deposition numbers 2303899 ([Cu_4_(L)_4_]-DMA), 2303900 ([Cu_4_(L)_3_(CH_3_L)]-DMA), 2303901 ([Cu_4_(L)_2_(CH_3_L)_2_]-DMA), and 2303902 ([Cu_4_(L)(CH_3_L)_3_]-DMA).

## Data availability

The data that support the findings of this study are openly available from the University of Strathclyde KnowledgeBase at https://doi.org/10.15129/abe4cc67-3802-4f83-af0f-d429a799bc3f.

## Author contributions

BD performed all synthetic procedures and collected IR, NMR, TGA, SEM, PXRD, SXRD, and N_2_ sorption data, as well as carrying out initial data interpretation. GAC conceived and supervised the project. ARK finalised the crystal structures and submitted the data to the CSD. ZW and SF collected CO_2_ gas sorption isotherms. AJF proposed suitable models and supervised the interpretation of the gas sorption data. All authors discussed the results and commented on the drafts of the manuscript written by BD and GAC.

## Conflicts of interest

There are no conflicts to declare.

## Supplementary Material

SC-015-D3SC06140J-s001

SC-015-D3SC06140J-s002
