# Supplementary material for: Improving the gas sorption capacity in lantern-type metal–organic polyhedra by a scrambled cage method
Source: Chem Sci. 2024 Jan 11;15(8):2857–66. doi: 10.1039/d3sc06140j (PMC10882442; doi:10.1039/d3sc06140j)
Supplement: SC-015-D3SC06140J-s001 [file SC-015-D3SC06140J-s001.pdf]

Electronic Supplementary Material (ESI) for Chemical Science.
This journal is © The Royal Society of Chemistry 2024

Supporting information for "Improving the gas sorption capacity in lantern-type metal-organic polyhedra by a scrambled cage method."

Beatriz Doñagueda Suso,[a] Zaoming Wang,[b] Alan R. Kennedy,[a] Ashleigh J. Fletcher,[c] Shuhei Furukawa,[b,d] and Gavin A. Craig*[a]

[a]*Department of Pure and Applied Chemistry, University of Strathclyde, Glasgow, G1 1XL, UK.* gavin.craig@strath.ac.uk

[b]*Institute for Integrated Cell-Material Sciences (WPI-iCeMS), Kyoto University, Yoshida, Sakyo-ku, Kyoto 606-8501, Japan.*

[c]*Department of Chemical and Process Engineering, University of Strathclyde, Glasgow, G1 1XJ, UK.*

[d]*Department of Synthetic Chemistry and Biological Chemistry, Graduate School of Engineering, Kyoto University, Katsura, Nishikyo-ku, Kyoto 615-8510, Japan.*

Table of contents

**Table S1.** Selected crystallographic data for compound $[Cu_4(L)_4]$-DMA	1
**Table S2.** Selected crystallographic data for **$[Cu_4(MeOL)_3(CH_3L)]$-DMA**, **$[Cu_4(MeOL)_2(CH_3L)_2]$-DMA**, and **$[Cu_4(MeOL)(CH_3L)_3]$-DMA**	2
**Table S3.** Selected crystallographic data for **$[Cu_4(L)_3(CH_3L)]$-DMA**, **$[Cu_4(L)_2(CH_3L)_2]$-DMA**, and **$[Cu_4(L)(CH_3L)_3]$-DMA**	3
**Fig. S1.** Representative crystal structure for the family $[Cu_4(MeOL)_{4-x}(CH_3L)_x]$-DMA ($x$= 1, 2, 3)	4
**Fig. S2.** PXRD data for **$[Cu_4(MeOL)_3(CH_3L)]$-DMA** calculated vs experimental	5
**Fig. S3.** PXRD data for **$[Cu_4(MeOL)_2(CH_3L)_2]$-DMA** calculated vs experimental	6
**Fig. S4.** PXRD data for **$[Cu_4(MeOL)(CH_3L)_3]$-DMA** calculated vs experimental	7
**Fig. S5.** IR spectra for family $[Cu_4(MeOL)_{4-x}(CH_3L)_x]$-DMA ($x$=0, 1, 2, 3, 4)	8
**Fig. S6.** PXRD data for family $[Cu_4(MeOL)_{4-x}(CH_3L)_x]$-DMA ($x$=0, 1, 2, 3, 4)	9
**Fig. S7.** IR spectra for **$[Cu_4(MeOL)_2(CH_3L)_2]$-DMA** versus **$[Cu_4(MeOL)_4]$-DMA + $[Cu_4(CH_3L)_4]$-DMA**	10
**Fig. S8.** PXRD data for $[Cu_4(MeOL)_{4-x}(CH_3L)_x]$-DMA ($x$=0, 2, 4) *vs.* physical mixture	11

Fig. S9. Full PXRD data for family [Cu_4(MeOL)$_{4-x}$(CH_3L)$_x$]-DMA (*x*=0, 2, 4) versus physical mixture 12

Fig. S10. TGA data for **[Cu_4(MeOL)$_3$(CH_3L)]-DMA** and **[Cu_4(MeOL)$_3$(CH_3L)]-MeOH** 13

Fig. S11. TGA data for **[Cu_4(MeOL)$_2$(CH_3L)$_2$]-DMA** and **[Cu_4(MeOL)$_2$(CH_3L)$_2$]-MeOH** 14

Fig. S12. TGA data for **[Cu_4(MeOL)(CH_3L)$_3$]-DMA** and **[Cu_4(MeOL)(CH_3L)$_3$]-MeOH** 15

Fig. S13. TGA data for **[Cu_4(MeOL)$_2$(CH_3L)$_2$]-DMA** and **[Cu_4(MeOL)$_4$]-DMA + [Cu_4(CH_3L)$_4$]-DMA** 16

Fig. S14. ^{1}H NMR spectrum for the digestion of **[Cu_4(MeOL)$_3$(CH_3L)]-DMA** 17

Fig. S15. ^{1}H NMR spectrum for the digestion of **[Cu_4(MeOL)$_2$(CH_3L)$_2$]-DMA** 17

Fig. S16. ^{1}H NMR spectrum for the digestion of **[Cu_4(MeOL)(CH_3L)$_3$]-DMA** 18

Fig. S17. SEM images for the family [Cu_4(MeOL)$_{4-x}$(CH_3L)$_x$]-DMA (*x*=0, 1, 2, 3, 4) 18

Fig. S18. Crystal structure of the cage **[Cu_4(L)$_3$(CH_3L)]-DMA** 19

Fig. S19. Crystal structure of the cage **[Cu_4(L)$_3$(CH_3L)]-DMA**, **[Cu_4(L)$_2$(CH_3L)$_2$]-DMA**, and **[Cu_4(L)(CH_3L)$_3$]-DMA** 20

Fig. S20. All possible compositions and their probability based on SXRD for the family [Cu_4(L)$_{4-x}$(CH_3L)$_x$]-DMA (*x* = 1, 2, 3). 21

Fig. S21. PXRD data for **[Cu_4(L)$_4$]-DMA** calculated vs experimental 22

Fig. S22. PXRD data for **[Cu_4(L)$_3$(CH_3L)]-DMA** calculated vs experimental 23

Fig. S23. PXRD data for **[Cu_4(L)$_2$(CH_3L)$_2$]-DMA** calculated vs experimental 24

Fig. S24. PXRD data for **[Cu_4(L)(CH_3L)$_3$]-DMA** calculated vs experimental 25

Fig. S25. IR spectra for family [Cu_4(L)$_{4-x}$(CH_3L)$_x$]-DMA (*x*=0, 1, 2, 3, 4) 26

Fig. S26. PXRD data for family [Cu_4(L)$_{4-x}$(CH_3L)$_x$]-DMA (*x*=0, 1, 2, 3, 4) 27

Fig. S27. TGA data for **[Cu_4(L)$_4$]-DMA** and **[Cu_4(L)$_4$]-MeOH** 28

Fig. S28. TGA data for **[Cu_4(L)$_3$(CH_3L)]-DMA** and **[Cu_4(L)$_3$(CH_3L)]-MeOH** 29

Fig. S29. TGA data for **[Cu_4(L)$_2$(CH_3L)$_2$]-DMA** and **[Cu_4(L)$_2$(CH_3L)$_2$]-MeOH** 30

Fig. S30. TGA data for **[Cu_4(L)(CH_3L)$_3$]-DMA** and **[Cu_4(L)(CH_3L)$_3$]-MeOH** 31

Fig. S31. ^{1}H NMR spectrum for the digestion of **[$Cu_4(L)_4$]-DMA** 32

Fig. S32. ^{1}H NMR spectrum for the digestion of **[$Cu_4(L)_3(CH_3L)$]-DMA** 32

Fig. S33. ^{1}H NMR spectrum for the digestion of **[$Cu_4(L)_2(CH_3L)_2$]-DMA** 33

Fig. S34. ^{1}H NMR spectrum for the digestion of **[$Cu_4(L)(CH_3L)_3$]-DMA** 33

Fig. S35. SEM images for the family [$Cu_4(L)_{4-x}(CH_3L)_x$]-DMA (*x*=0, 1, 2, 3, 4) 34

Fig. S36. IR spectra for **[$Cu_4(L)_2(CH_3L)_2$]-DMA** versus **[$Cu_4(L)_4$]-DMA + [$Cu_4(CH_3L)_4$]-DMA** 34

Fig. S37. PXRD data for family [$Cu_4(L)_{4-x}(CH_3L)_x$]-DMA (*x*=0, 2, 4) versus physical mixture 35

Fig. S38. TGA data for **[$Cu_4(L)_2(CH_3L)_2$]-DMA** and **[$Cu_4(L)_4$]-DMA + [$Cu_4(CH_3L)_4$]-DMA** 36

Fig. S39. IR spectra for family [$Cu_4(L)_{4-x}(CH_3L)_x$]-MeOH (*x*=0, 1, 2, 3, 4) 37

Fig. S40. IR spectra for family [$Cu_4(MeOL)_{4-x}(CH_3L)_x$]-MeOH (*x*=0, 1, 2, 3, 4) 38

Fig. S41. ^{1}H NMR spectrum for the digestion of **[$Cu_4(MeOL)_3(CH_3L)$]-MeOH** 39

Fig. S42. ^{1}H NMR spectrum for the digestion of **[$Cu_4(MeOL)_2(CH_3L)_2$]-MeOH** 39

Fig. S43. ^{1}H NMR spectrum for the digestion of **[$Cu_4(MeOL)(CH_3L)_3$]-MeOH** 40

Fig. S44. ^{1}H NMR spectrum for the digestion of **[$Cu_4(L)_4$]-MeOH** 40

Fig. S45. ^{1}H NMR spectrum for the digestion of **[$Cu_4(L)_3(CH_3L)$]-MeOH** 41

Fig. S46. ^{1}H NMR spectrum for the digestion of **[$Cu_4(L)_2(CH_3L)_2$]-MeOH** 41

Fig. S47. ^{1}H NMR spectrum for the digestion of **[$Cu_4(L)(CH_3L)_3$]-MeOH** 42

Fig. S48. PXRD data for family [$Cu_4(MeOL)_{4-x}(CH_3L)_x$]-MeOH (*x*=0, 1, 2, 3, 4) 42

Fig. S49. PXRD data for family [$Cu_4(MeOL)_{4-x}(CH_3L)_x$]-MeOH (*x*=0, 2, 4) versus physical mixture 43

Fig. S50. PXRD data for family [$Cu_4(L)_{4-x}(CH_3L)_x$]-MeOH (*x*=0, 1, 2, 3, 4) 44

Fig. S51. PXRD data for family [$Cu_4(L)_{4-x}(CH_3L)_x$]-MeOH (*x*=0, 2, 4) versus physical mixture 45

Fig. S52. SEM images for the family [$Cu_4(MeOL)_{4-x}(CH_3L)_x$]-MeOH (*x*=0, 1, 2, 3, 4) 46

Fig. S53. SEM images for the family [$Cu_4(L)_{4-x}(CH_3L)_x$]-MeOH (x=0, 1, 2, 3, 4) 46

Fig. S54. N_2 isotherm and Dubinin-Radishkevich analysis for **[$Cu_4(MeOL)_3(CH_3L)$]** 47

Fig. S55. Rouquerol and BET analysis for **[$Cu_4(MeOL)_3(CH_3L)$]** 48

Fig. S56. N_2 isotherm and Dubinin-Radishkevich analysis for **[$Cu_4(MeOL)_2(CH_3L)_2$]** 49

Fig. S57. Rouquerol and BET analysis for **[$Cu_4(MeOL)_2(CH_3L)_2$]** 50

Fig. S58. N_2 isotherm and Dubinin-Radishkevich analysis for **[$Cu_4(MeOL)(CH_3L)_3$]** 51

Fig. S59. Rouquerol and BET analysis for **[$Cu_4(MeOL)(CH_3L)_3$]** 52

Fig. S60. N_2 isotherm and BET surface area analysis for **[$Cu_4(MeOL)_4$] + [$Cu_4(CH_3L)_4$]** 53

Fig. S61. CO_2 isotherm for **[$Cu_4(MeOL)_3(CH_3L)$]** 54

Fig. S62.CO_2 isotherm for **[$Cu_4(MeOL)_2(CH_3L)_2$]** 54

Fig. S63. CO_2 isotherm for **[$Cu_4(MeOL)(CH_3L)_3$]** 55

Fig. S64. CO_2 isotherm for the family [$Cu_4(MeOL)_{4-x}(CH_3L)_x$] ($x$=0, 1, 2, 3, 4) 55

Fig. S65. IR spectra of **[$Cu_4(MeOL)_3(CH_3L)$]** post sorption 56

Fig. S66. IR spectra of **[$Cu_4(MeOL)_2(CH_3L)_2$]** post sorption 57

Fig. S67. IR spectra of **[$Cu_4(MeOL)(CH_3L)_3$]** post sorption 58

Fig. S68. PXRD data for **[$Cu_4(MeOL)_3(CH_3L)$]** post sorption 59

Fig. S69. PXRD data for **[$Cu_4(MeOL)_2(CH_3L)_2$]** post sorption 60

Fig. S70. PXRD data for **[$Cu_4(MeOL)(CH_3L)_3$]** post sorption 61

Fig. S71. ^{1}H NMR spectrum for the digestion of **[$Cu_4(MeOL)_3(CH_3L)$]** post sorption 62

Fig. S72. ^{1}H NMR spectrum for the digestion of **[$Cu_4(MeOL)_2(CH_3L)_2$]** post sorption 62

Fig. S73. ^{1}H NMR spectrum for the digestion of **[$Cu_4(MeOL)(CH_3L)_3$]** post soprtion 63

Fig. S74. N_2 isotherm and Dubinin-Radishkevich analysis for **[$Cu_4(L)_4$]** 64

Fig. S75. Rouquerol and BET analysis for **[$Cu_4(L)_4$]** 65

Fig. S76. N_2 isotherm and BET surface area analysis for **[$Cu_4(L)_3(CH_3L)$]** 66

Fig. S77. Rouquerol and BET analysis for **[$Cu_4(L)_3(CH_3L)$]** 67

Fig. S78. N_2 isotherm and BET surface area analysis for **[$Cu_4(L)_2(CH_3L)_2$]** 68

Fig. S79. Rouquerol and BET analysis for **[$Cu_4(L)_2(CH_3L)_2$]** 69

Fig. S80. N_2 isotherm and BET surface area analysis for **[$Cu_4(L)(CH_3L)_3$]** 70

Fig. S81. Rouquerol and BET analysis for **[$Cu_4(L)(CH_3L)_3$]** 71

Fig. S82. N_2 isotherm and BET surface area analysis for **[$Cu_4(L)_4$] + [$Cu_4(CH_3L)_4$]** 72

Fig. S83. CO_2 isotherm for **[$Cu_4(L)_4$]** 73

Fig. S84. CO_2 isotherm for **[$Cu_4(L)_3(CH_3L)$]** 73

Fig. S85. CO_2 isotherm for **[$Cu_4(L)_2(CH_3L)_2$]** 74

Fig. S86. CO_2 isotherm for **[$Cu_4(L)(CH_3L)_3$]** 74

Fig. S87. ^1H NMR spectrum for the digestion of **[$Cu_4(L)_4$]** post sorption 75

Fig. S88. ^1H NMR spectrum for the digestion of **[$Cu_4(L)_3(CH_3L)$]** post sorption 75

Fig. S89. ^1H NMR spectrum for the digestion of **[$Cu_4(L)_2(CH_3L)_2$]** post sorption 76

Fig. S90. ^1H NMR spectrum for the digestion of **[$Cu_4(L)(CH_3L)_3$]** post soprtion 76

Fig. S91. IR spectra of **[$Cu_4(L)_4$]** post sorption 77

Fig. S92. IR spectra of **[$Cu_4(L)_3(CH_3L)$]** post sorption 78

Fig. S93. IR spectra of **[$Cu_4(L)_2(CH_3L)_2$]** post sorption 79

Fig. S94. IR spectra of **[$Cu_4(L)(CH_3L)_3$]** post sorption 80

Fig. S95. PXRD data for **[$Cu_4(L)_4$]** post sorption 81

Fig. S96. PXRD data for **[$Cu_4(L)_3(CH_3L)$]** post sorption 82

Fig. S97. PXRD data for **[$Cu_4(L)_2(CH_3L)_2$]** post sorption 83

Fig. S98. PXRD data for **[$Cu_4(L)(CH_3L)_3$]** post sorption 84

Single Crystal X-ray diffraction details 85

Table S1. Selected single crystal x-ray diffraction crystallographic data for compound [$Cu_4(L)_4$]-DMA.

	**[$Cu_4(L)_4$]-DMA, CCDC 2303899**
λ (Å)	1.54184
T (K)	100(2)
Crystal System	Monoclinic
Space Group	*P21/n*
a (Å)	19.7309(2)
b (Å)	16.8006(2)
c (Å)	24.9013(2)
α (°)	90
β (°)	93.538(1)
γ (°)	90
V (Å^3)	8238.82(15)
Z	2
$2\vartheta_{max}$ (°)	146.478
Reflections	88490
Unique Data	16376
Observed Data	14452
No. Parameters	854
R_{int}	0.0294
$R[F^2 > 2\sigma(F^2)]$	0.0782
$wR(F^2)$	0.2468
S	1.047
ρ_{max}, ρ_{min} ($eÅ^{-3}$)	0.984/-0.717

Table S2. Selected crystallographic data for the scrambled cages in the family $[Cu_4(MeOL)_{4-x}(CH_3L)_x]$-DMA (*x*=1, 2, 3). These data are taken from attempts to resolve the single-crystal structures using data collected at the University of Strathclyde, however the data were not good enough to enable accurate determination of atomic positions beyond the lantern core. An example showing the connectivity is given in Fig. S1.

	**$[Cu_4(MeOL)_3(CH_3L)]$-DMA**	**$[Cu_4(MeOL)_2(CH_3L)_2]$-DMA**	**$[Cu_4(MeOL)(CH_3L)_3]$-DMA**
λ (Å)		1.54184	
T (K)		100(2)	
Crystal System	Monoclinic	Monoclinic	Monoclinic
Space Group	$P2_1/n$	$P2_1/n$	$P2_1/n$
a (Å)	20.0054(3)	19.8970(2)	19.9223(2)
b (Å)	17.1240(3)	17.0838(3)	16.9302(2)
c (Å)	26.1957(4)	25.9597(4)	25.7928(3)
α (°)	90	90	90
β (°)	98.173(1)	97.294(1)	96.814(1)
γ (°)	90	90	90
V (Å^3)	8882.8(3)	8752.7(2)	8638.17(17)
Z	2	2	2

Table S3. Selected crystallographic data from single crystal X-ray diffraction for the scrambled cages in the family $[Cu_4(L)_{4-x}(CH_3L)_x]$-DMA (*x*=1, 2, 3)

	**$[Cu_4(L)_3(CH_3L)]$-DMA**	**$[Cu_4(L)_2(CH_3L)_2]$-DMA**	**$[Cu_4(L)(CH_3L)_3]$-DMA**
λ (Å)		1.54184	
T (K)		100(2)	
CCDC ref no.	2303900	2303901	2303902
Crystal System	Monoclinic	Monoclinic	Monoclinic
Space Group	$P2_1/n$	$P2_1/n$	$P2_1/n$
a (Å)	19.9031(2)	19.9329(2)	19.8340(2)
b (Å)	16.7668(1)	16.7488(2)	16.8359(2)
c (Å)	24.9964(2)	25.2197(2)	25.5694(2)
α (°)	90	90	90
β (°)	94.081(1)	94.831(1)	96.331(1)
γ (°)	90	90	90
V ($Å^3$)	8320.43(12)	8389.74(15)	8486.14(15)
Z	2	2	2
$2\vartheta_{max}$ (°)	146.596	146.480	146.358
Reflections	89644	91065	93791
Unique Data	16543	16741	16852
Observed Data	14251	14832	14047
No. Parameters	982	993	892
R_{int}	0.0353	0.0341	0.0405
$R[F^2 > 2\sigma(F^2)]$	0.0995	0.1050	0.1366
$wR(F^2)$	0.3470	0.3660	0.4323
S	1.610	1.709	1.965
ρ_{max}, ρ_{min} ($eÅ^{-3}$)	1.275/-0.847	1.165/-0.863	1.627/-0.852

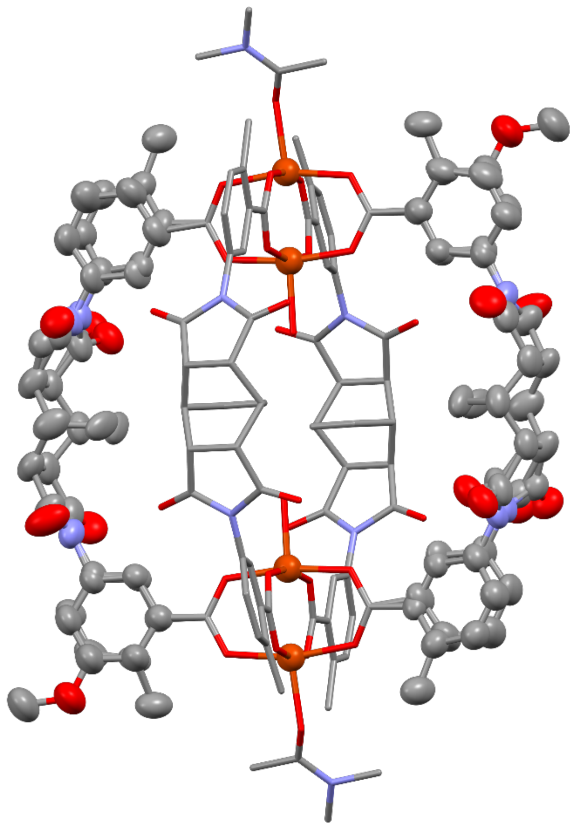

Figure S1. Crystal structure for the cage **[Cu_4(MeOL)(CH_3L)$_3$]-DMA** showing connectivity of the lantern cage. The disordered ligands in the cage could not be properly refined to obtain occupancies of the attached methoxy- or methyl- groups. Hydrogen atoms and solvent molecules in the void have been omitted for clarity. Rust, red, grey and light blue represent copper, oxygen, carbon and nitrogen atoms respectively.

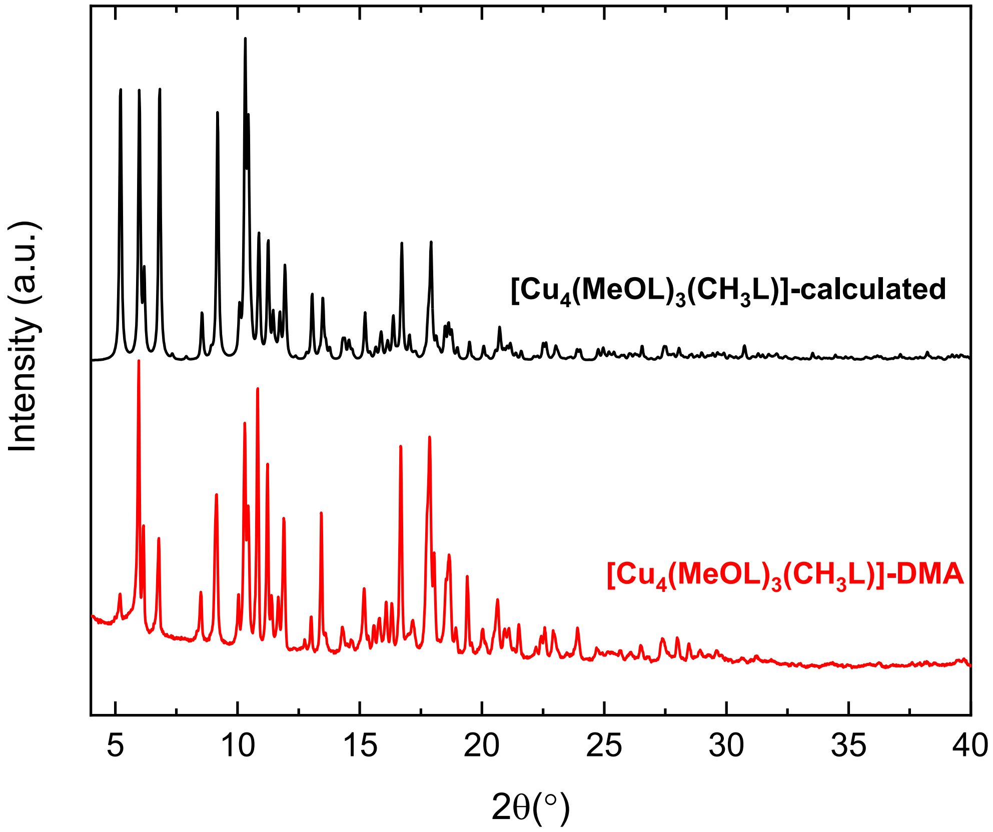

Figure S2. Powder X-ray diffractograms calculated for **[Cu_4(MeOL)$_3$(CH_3L)]-DMA** from .cif files (black) and collected on the sample of **[Cu_4(MeOL)$_3$(CH_3L)]-DMA** (red).

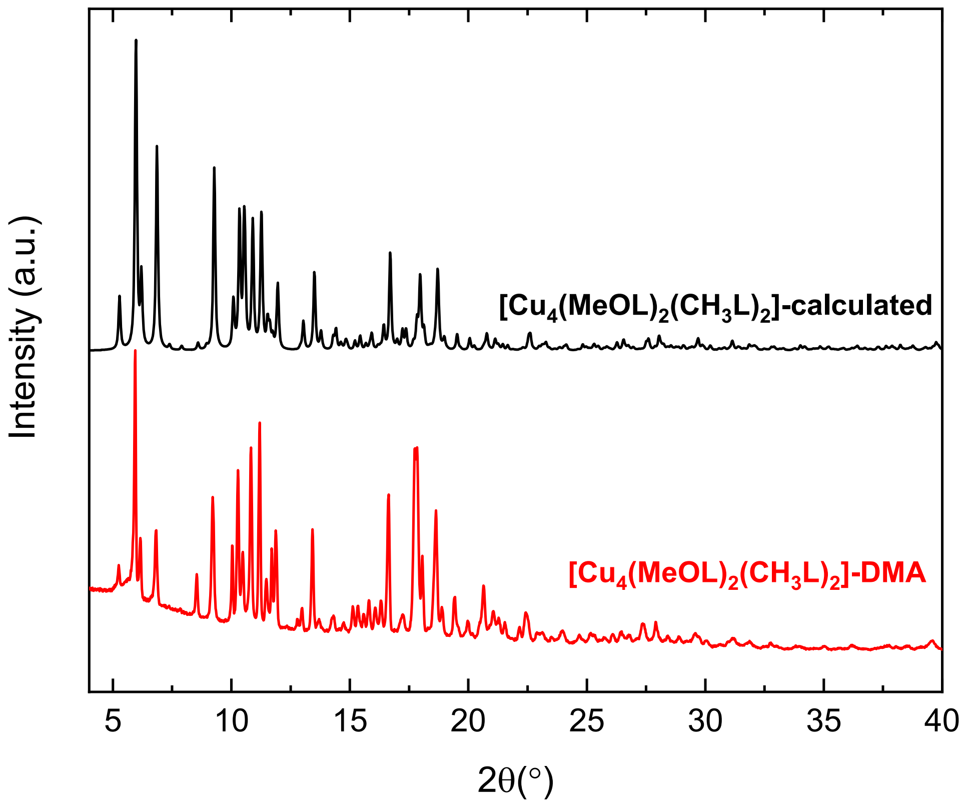

Figure S3. Powder X-ray diffractograms calculated for **[$Cu_4(MeOL)_2(CH_3L)_2$]-DMA** from .cif files (black) and collected on the sample of **[$Cu_4(MeOL)_2(CH_3L)_2$]-DMA** (red).

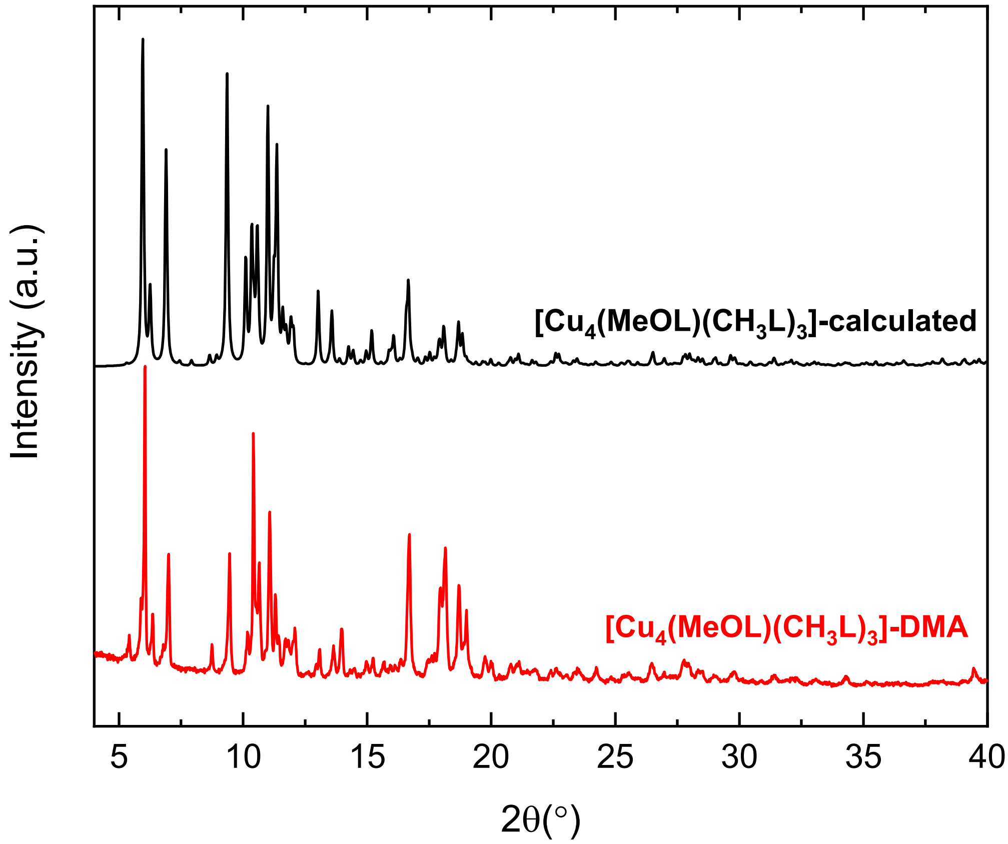

Figure S4. Powder X-ray diffractograms calculated for **$[Cu_4(MeOL)(CH_3L)_3]$-DMA** from .cif files (black) and collected on the sample of **$[Cu_4(MeOL)(CH_3L)_3]$-DMA** (red).

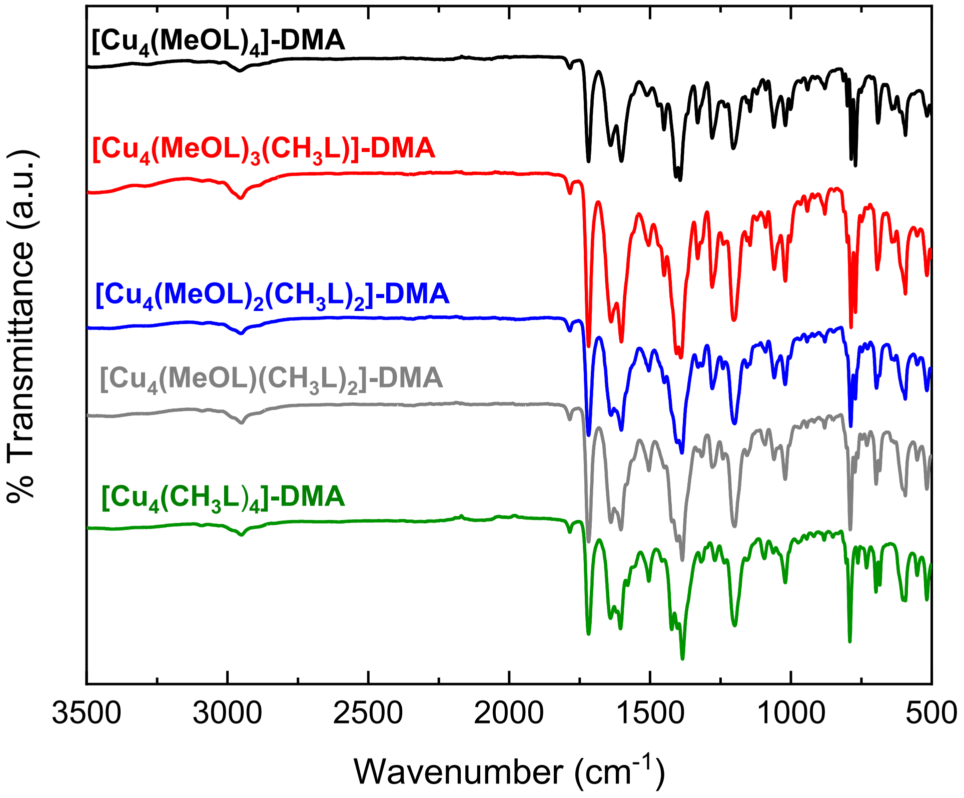

Figure S5. IR spectra of the as-synthesised phases for the family $[Cu_4(MeOL)_{4-x}(CH_3L)_x]$-DMA (*x*=0, 1, 2, 3, 4).

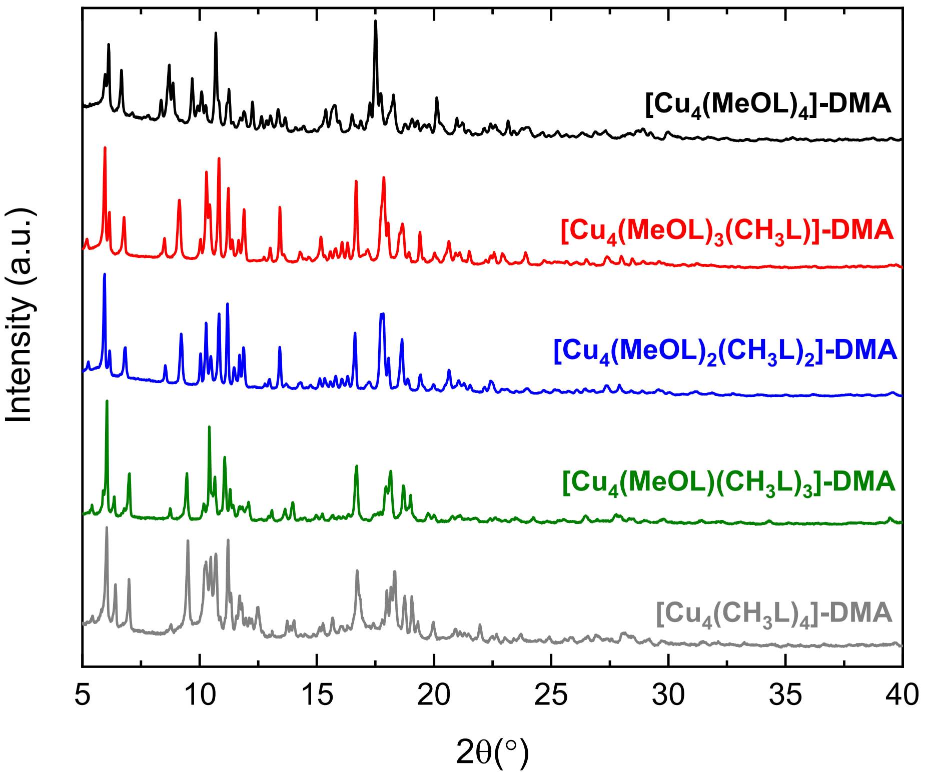

Figure S6. Powder X-ray diffractogram pattern of the as-synthesised family $[Cu_4(MeOL)_{4-x}(CH_3L)_x]$-DMA ($x$=0, 1, 2, 3, 4).

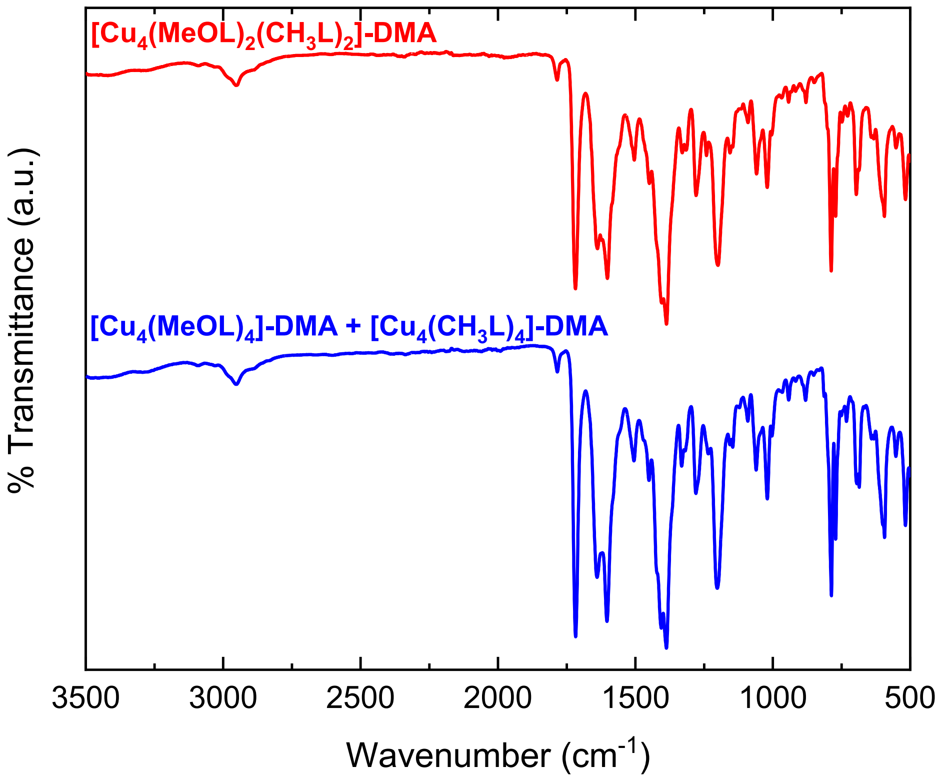

Figure S7. IR spectra of the scrambled cage **[$Cu_4(MeOL)_2(CH_3L)_2$]-DMA** (red) and the physical mixture **[$Cu_4(MeOL)_4$]-DMA + [$Cu_4(CH_3L)_4$]-DMA** (blue).

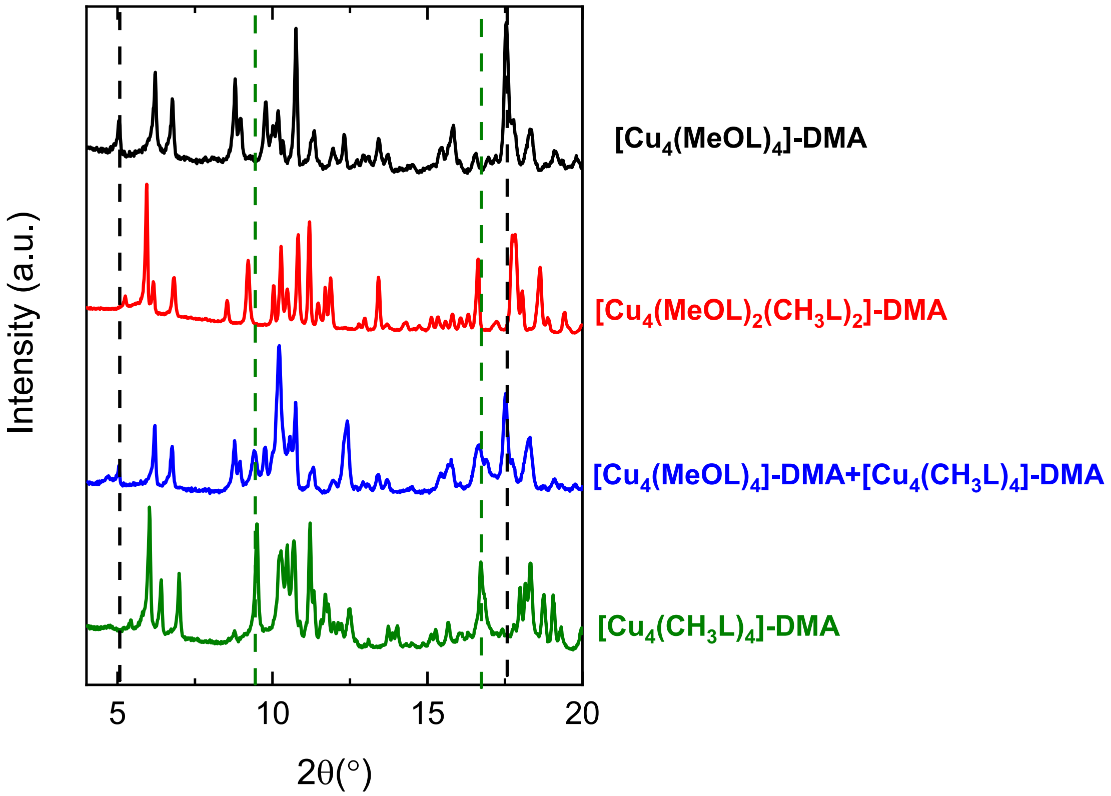

Figure S8. Powder X-ray diffractogram of as-synthesised phases for bulk homoleptic cage **[Cu$_4$(MeOL)$_4$]** (black), scrambled cage **[Cu$_4$(MeOL)$_2$(CH$_3$L)$_2$]** (red), physical mixture **[Cu$_4$(MeOL)$_4$]-DMA + [Cu$_4$(CH$_3$L)$_4$]-DMA** and bulk sample of homoleptic cage **[Cu$_4$(CH$_3$L)$_4$]** (olive). The dashed lines correspond to 2ϑ = 5.07, 9.44, 16.74 and 17.57°.

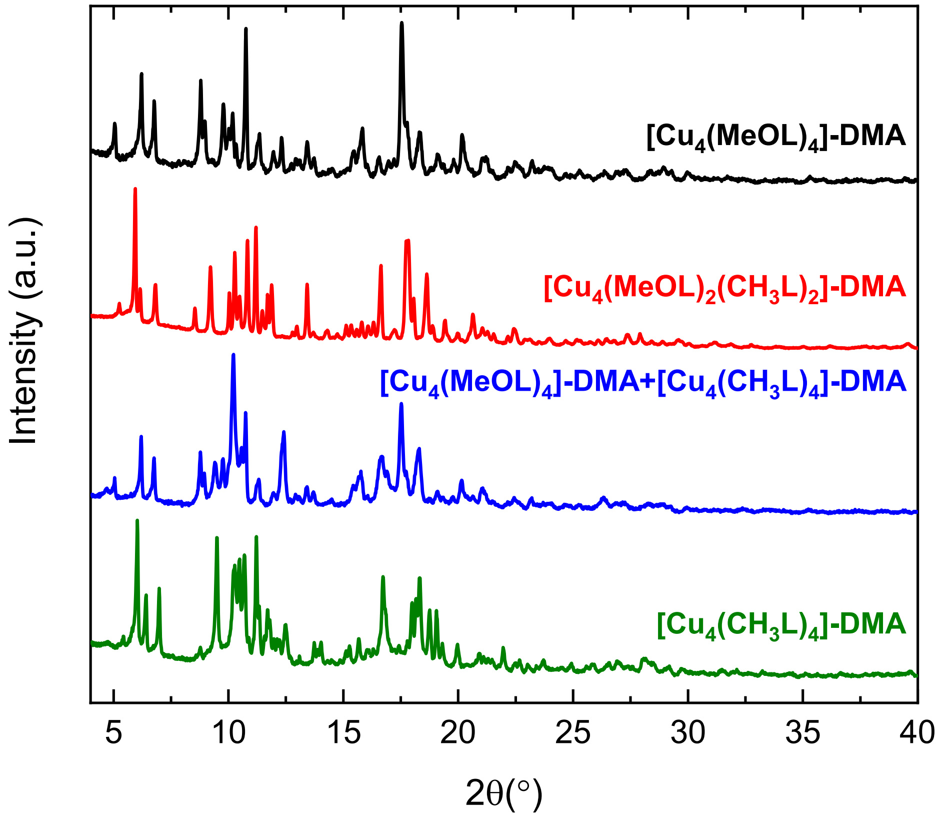

Figure S9. Full powder X-ray diffractogram of bulk homoleptic cage **$[Cu_4(MeOL)_4]$-DMA** (black), scrambled cage **$[Cu_4(MeOL)_2(CH_3L)_2]$-DMA** (red), physical mixture **$[Cu_4(MeOL)_4]$-DMA + $[Cu_4(CH_3L)_4]$-DMA** and bulk sample of homoleptic cage **$[Cu_4(CH_3L)_4]$-DMA** (olive).

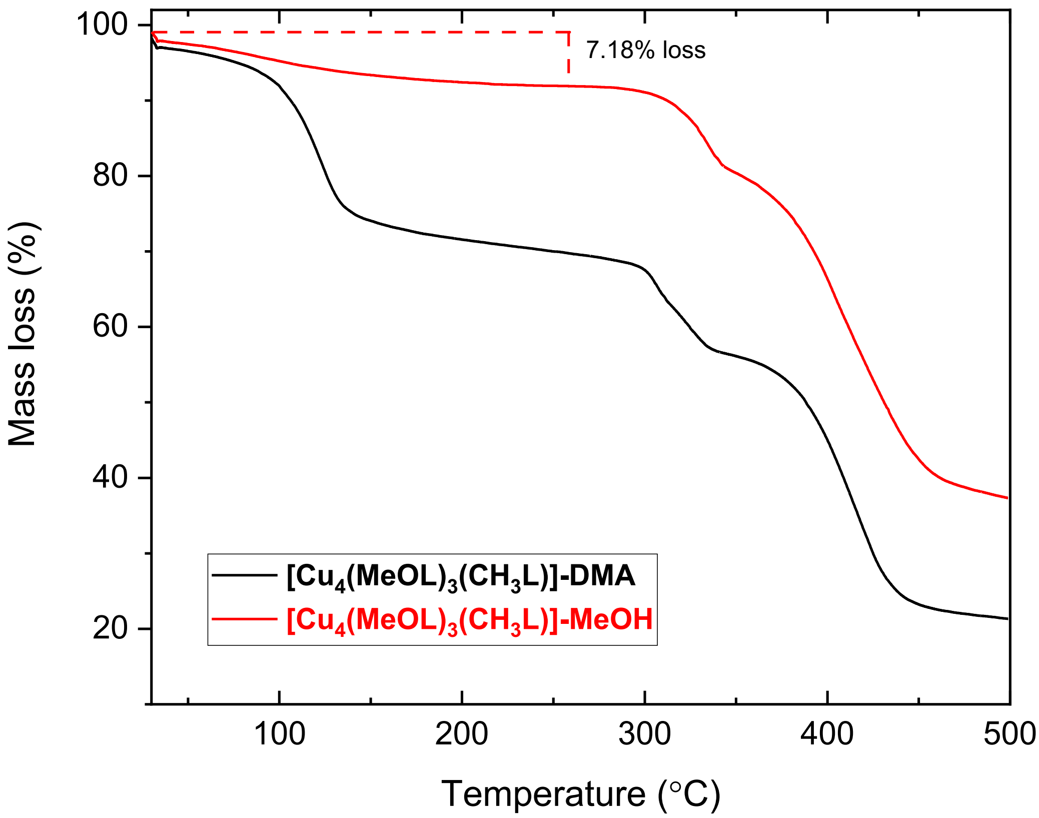

Figure S10. TGA trace of sample **[Cu_4(MeOL)$_3$(CH_3L)]-DMA** (black) and after solvent exchange with MeOH **[Cu_4(MeOL)$_3$(CH_3L)]-MeOH** (red). The mass loss in **[Cu_4(MeOL)$_3$(CH_3L)]-DMA** is divided into three main steps before decomposition, which are attributed to the loss of solvent from the pores up to approximately 140 °C, before loss of solvent attached to the paddlewheels and decomposition just above 300 °C. The trace for **[Cu_4(MeOL)$_3$(CH_3L)]-MeOH** shows mass loss of 7.18 % which is attributed to the loss of non-coordinated solvent before entering a plateau at around 260 °C. Based on the TGA trace of MeOH exchanged phase, the proposed formula for **[Cu_4(MeOL)$_3$(CH_3L)]-MeOH** is [Cu_4(MeOL)$_3$(CH_3L)(MeOH)$_2$(H_2O)$_2$]·5 MeOH.

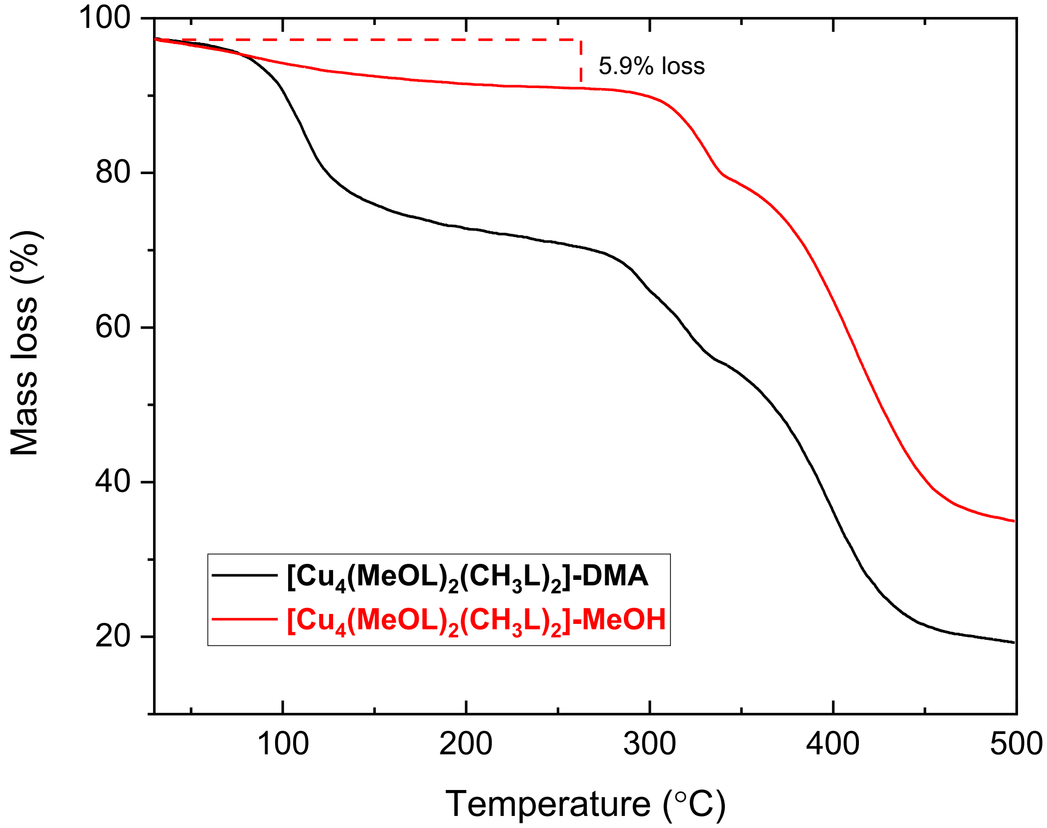

Figure S11. TGA trace of sample **[$Cu_4(MeOL)_2(CH_3L)_2$]-DMA** (black) and after solvent exchange with MeOH **[$Cu_4(MeOL)_2(CH_3L)_2$]-MeOH** (red). The mass loss in **[$Cu_4(MeOL)_2(CH_3L)_2$]-DMA** is divided into three main steps before decomposition, which are attributed to the loss of solvent from the pores up to approximately 150 °C, before loss of solvent attached to the paddlewheels and decomposition just above 270 °C. The trace for **[$Cu_4(MeOL)_2(CH_3L)_2$]-MeOH** shows mass loss of 5.9 % which is attributed to the loss of non-coordinated solvent before entering a plateau at around 260 °C. Based on the TGA trace of the MeOH exchanged phase, the proposed formula for **[$Cu_4(MeOL)_2(CH_3L)_2$]-MeOH** is [$Cu_4(MeOL)_2(CH_3L)_2(MeOH)_2(H_2O)_2$]·4 MeOH

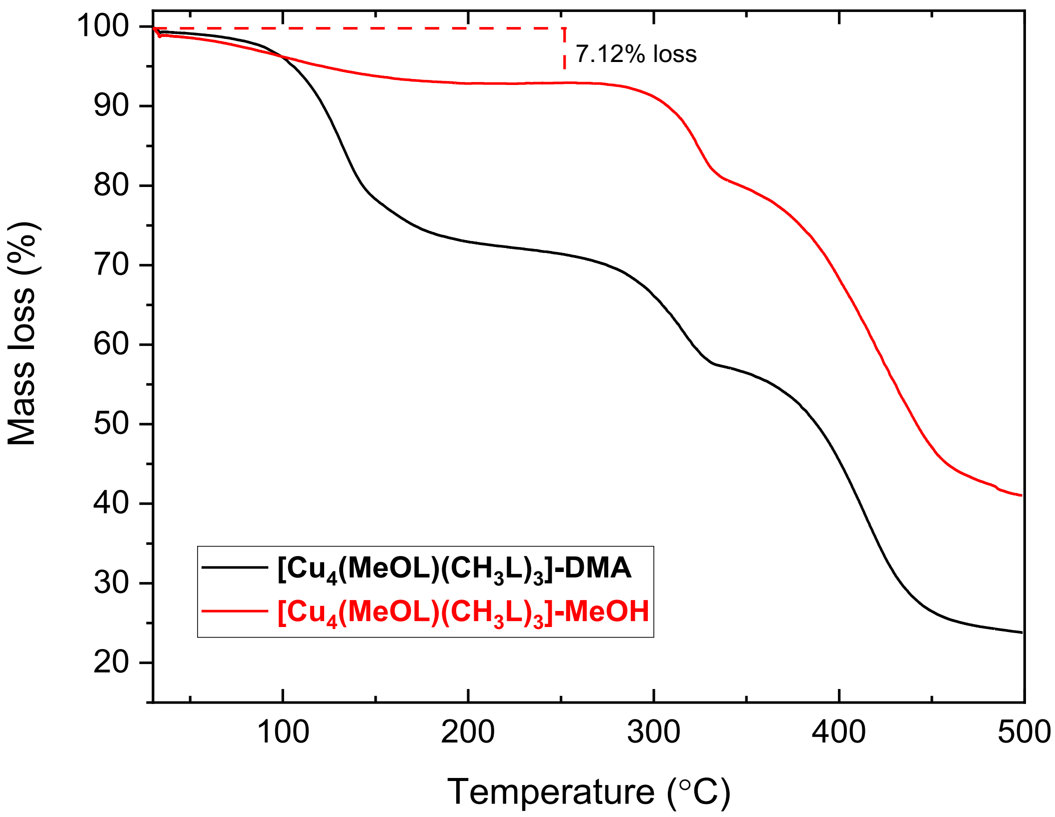

Figure S12. TGA trace of sample **[Cu_4(MeOL)(CH_3L)$_3$]-DMA** (black) and after solvent exchange with MeOH **[Cu_4(MeOL)(CH_3L)$_3$]-MeOH** (red). The mass loss in **[Cu_4(MeOL)(CH_3L)$_3$]-DMA** is divided into three main steps before decomposition, which are attributed to the loss of solvent from the pores up to approximately 160 °C, before loss of solvent attached to the paddlewheels and decomposition just above 270 °C. The trace for **[Cu_4(MeOL)(CH_3L)$_3$]-MeOH** shows mass loss of 7.12 % which is attributed to the loss of non-coordinated solvent before entering a plateau at around 250 °C. Based on the TGA trace of MeOH exchanged phase, the proposed formula for **[Cu_4(MeOL)(CH_3L)$_3$]-MeOH** is [Cu_4(MeOL)(CH_3L)$_3$(MeOH)$_2$(H_2O)$_2$]·5 MeOH.

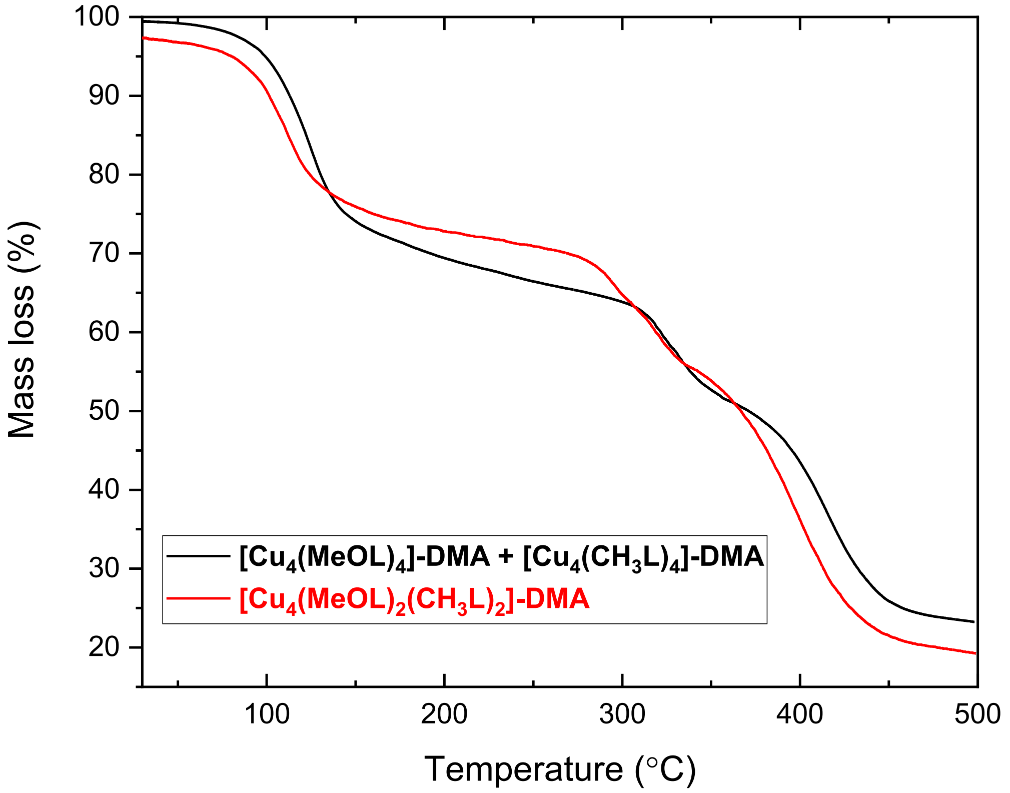

Figure S13. TGA trace of the scrambled cage **[$Cu_4(MeOL)_2(CH_3L)_2$]-DMA** (red) and the physical mixture **[$Cu_4(MeOL)_4$]-DMA + [$Cu_4(CH_3L)_4$]-DMA** (black) showing different degradation temperatures and different TGA traces.

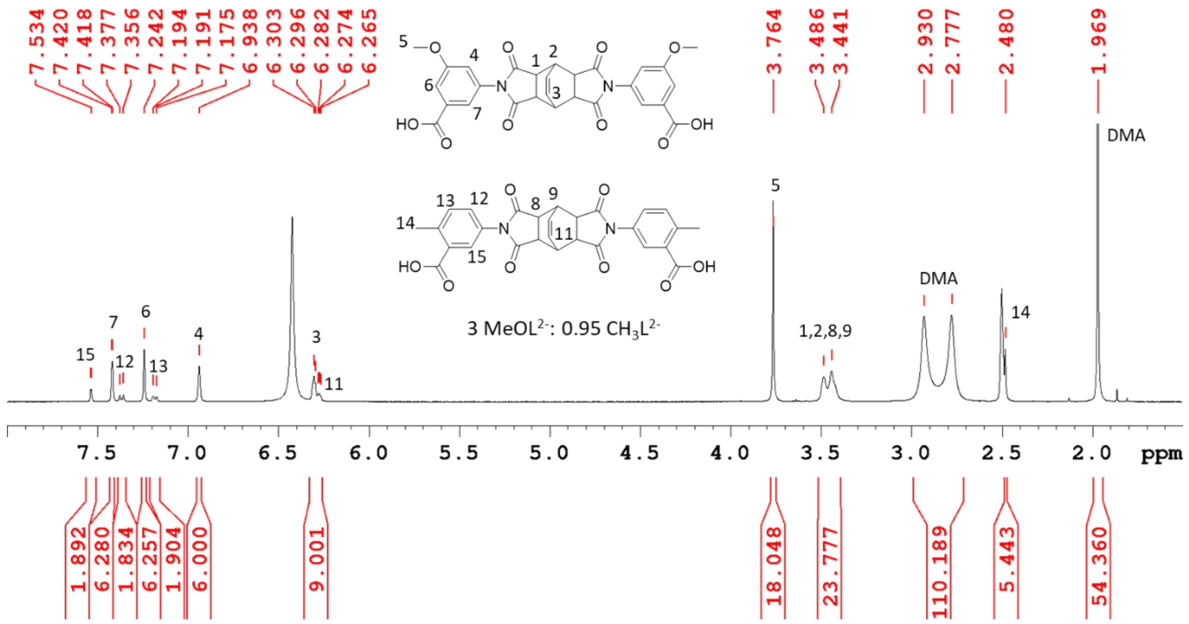

Figure S14. ^{1}H-NMR spectrum of the digestion in DMSO/DCl of **[$Cu_4(MeOL)_3(CH_3L)$]-DMA** showing the presence of 18 DMA in the structure and a ratio of 3 : 0.95 for MeOL2^{2-}:CH_3L^{2-}.

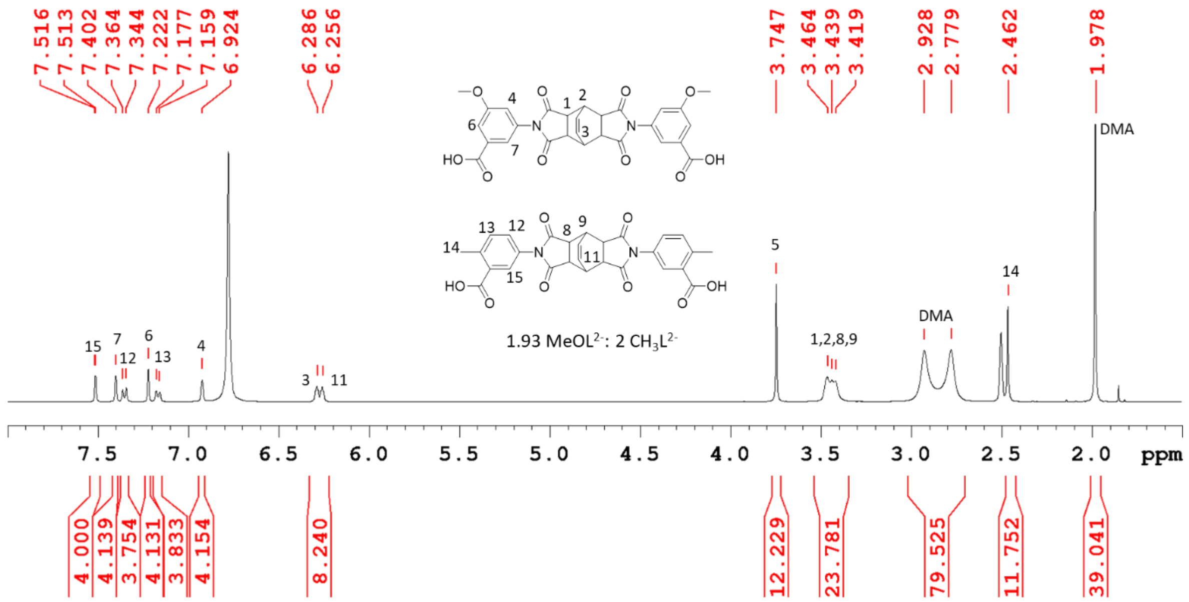

Figure S15. ^{1}H-NMR spectrum of the digestion in DMSO/DCl of **[$Cu_4(MeOL)_2(CH_3L)_2$]-DMA** showing the presence of 13 DMA in the structure and a ratio of 1.93 : 2 for MeOL2^{2-}:CH_3L^{2-}.

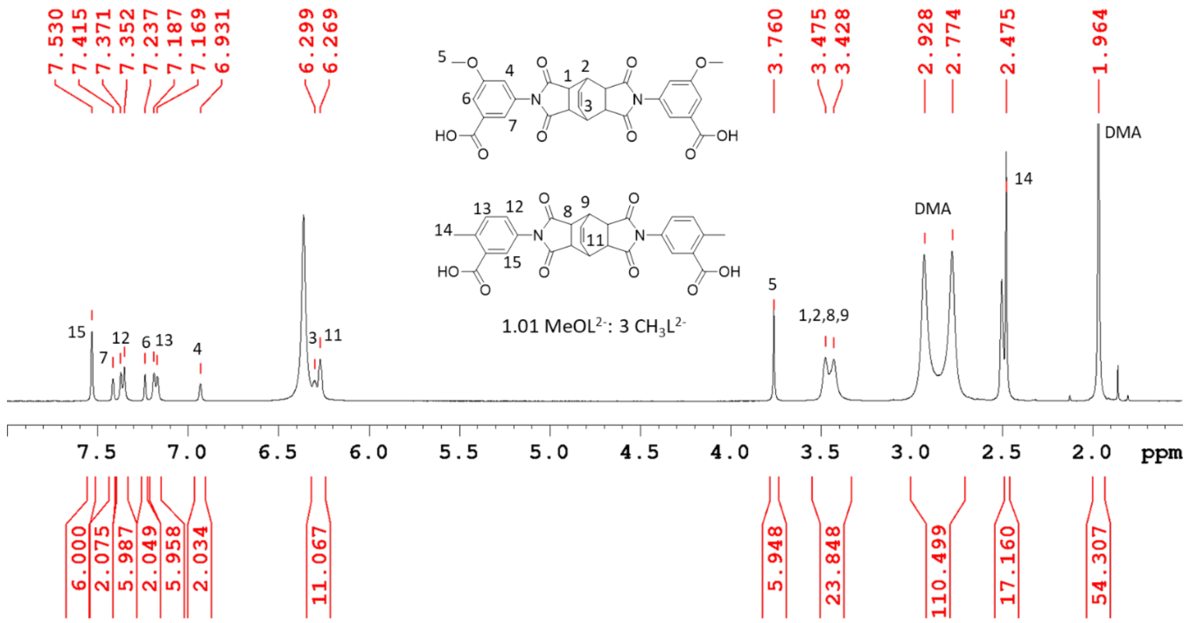

Figure S16. ^{1}H-NMR spectrum of the digestion in DMSO/DCl of **[Cu_4(MeOL)(CH_3L)$_3$]-DMA** showing the presence of 18 DMA in the structure and a ratio of 1.01 : 3 for MeOL2^{2-}:CH_3L^{2-}.

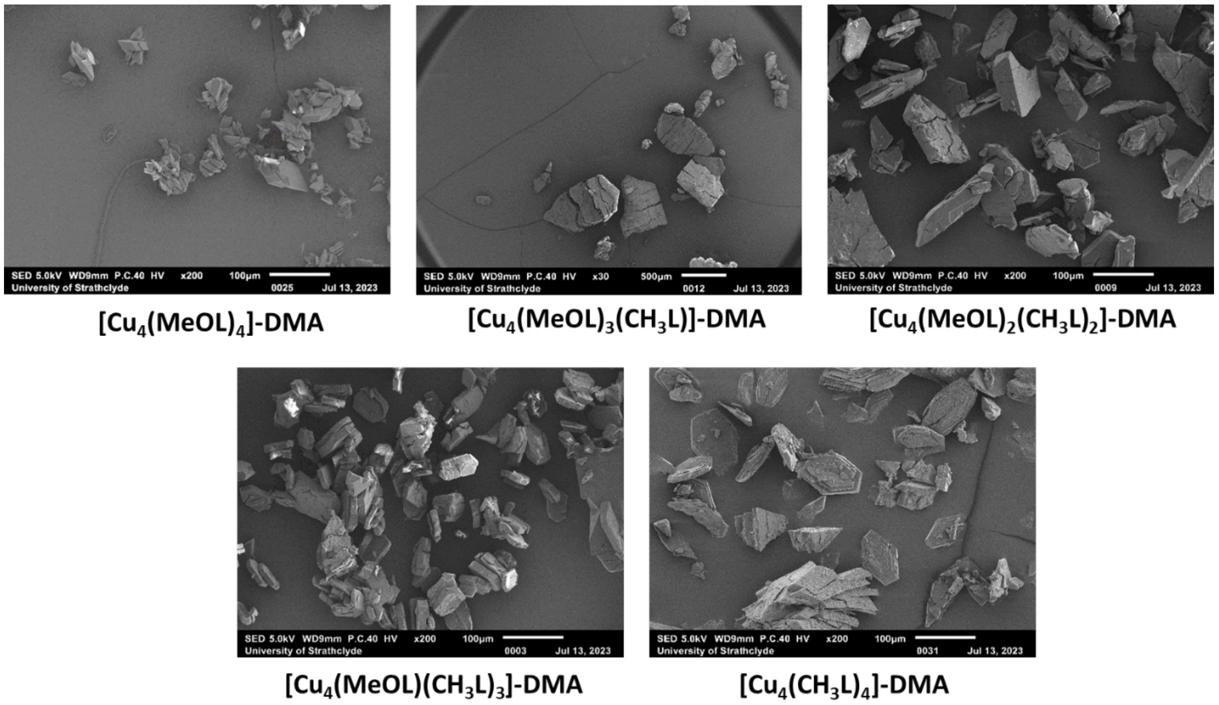

Figure S17. SEM images for the family [Cu_4(MeOL)$_{4-x}$(CH_3L)$_x$]-DMA (x=0, 1, 2, 3, 4). Magnification used is x200.

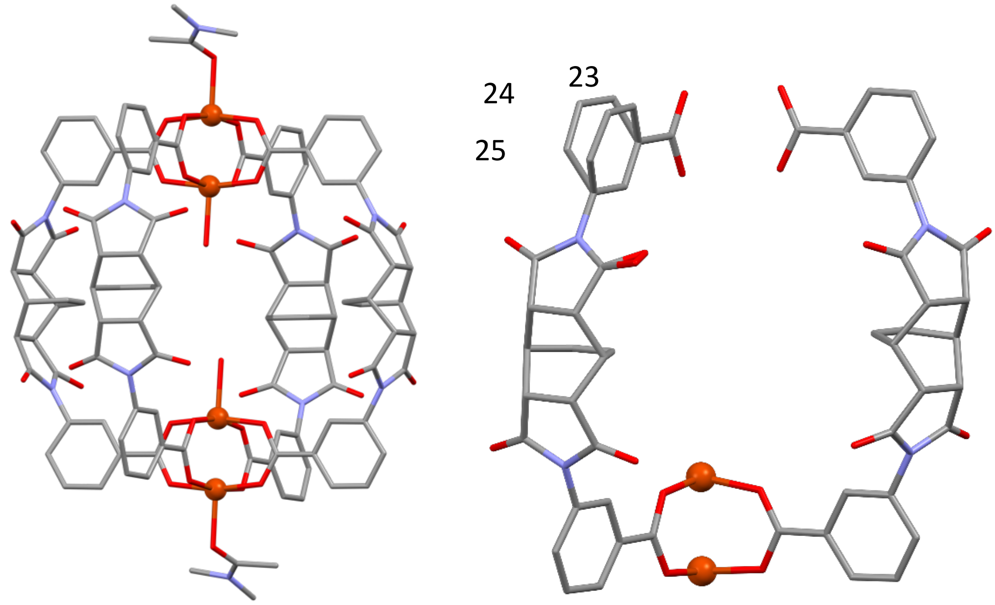

Figure S18. Left: Crystal structure of the cage **[Cu_4(L)$_4$]-DMA** showing DMA molecules coordinated to the outer axial sites of the paddlewheel and water molecules coordinated to the inner sites of the copper paddlewheel. Hydrogen atoms and solvent molecules in the void have been omitted for clarity. Right: asymmetric unit showing modelled disorder in the atoms C23, C24 and C25 over two positions. Hydrogen atoms and all solvent molecules have been omitted for clarity. Rust, red, grey and light blue represent copper, oxygen, carbon and nitrogen atoms respectively in both cases.

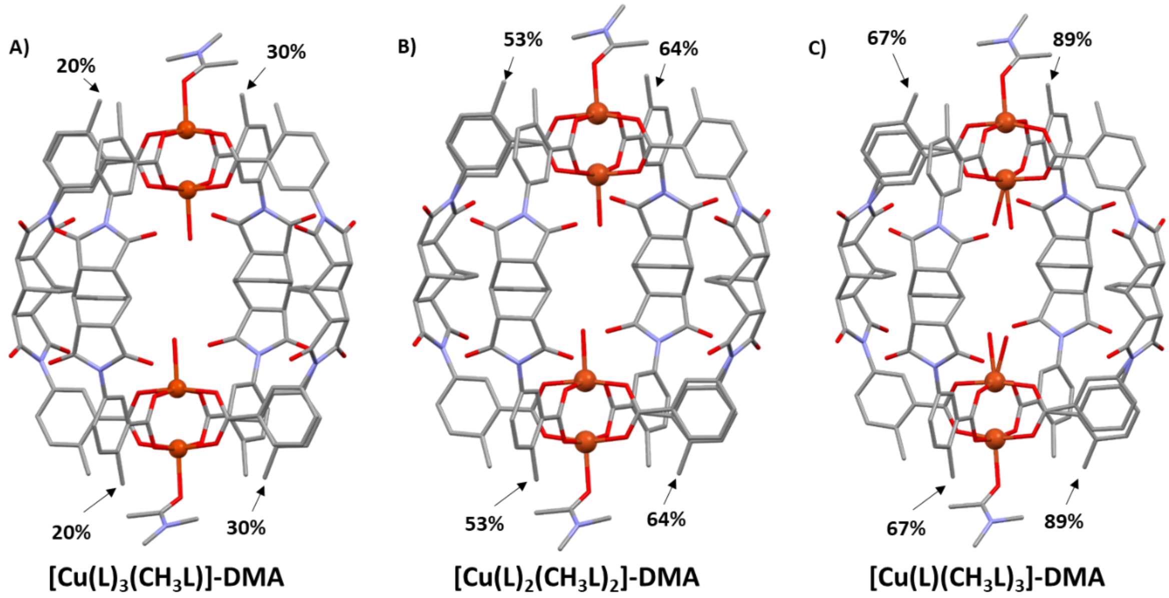

Figure S19. A) Crystal structure of the cage **[$Cu_4(L)_3(CH_3L)$]-DMA** and the relative occupancies for each methyl group. B) Crystal structure of the cage **[$Cu_4(L)_2(CH_3L)_2$]-DMA** and the relative occupancies for each methyl group. C) Crystal structure of the cage **[$Cu_4(L)(CH_3L)_3$]-DMA** and the relative occupancies for each methyl group. All of the relative occupancies are represented as a percentage. All of them show DMA molecules coordinated to the outer axial sites of the paddlewheel and water molecules coordinated to the inner sites of the copper paddlewheel. Hydrogen atoms and solvent molecules in the void have been omitted for clarity. Rust, red, grey and light blue represent copper, oxygen, carbon and nitrogen atoms respectively.

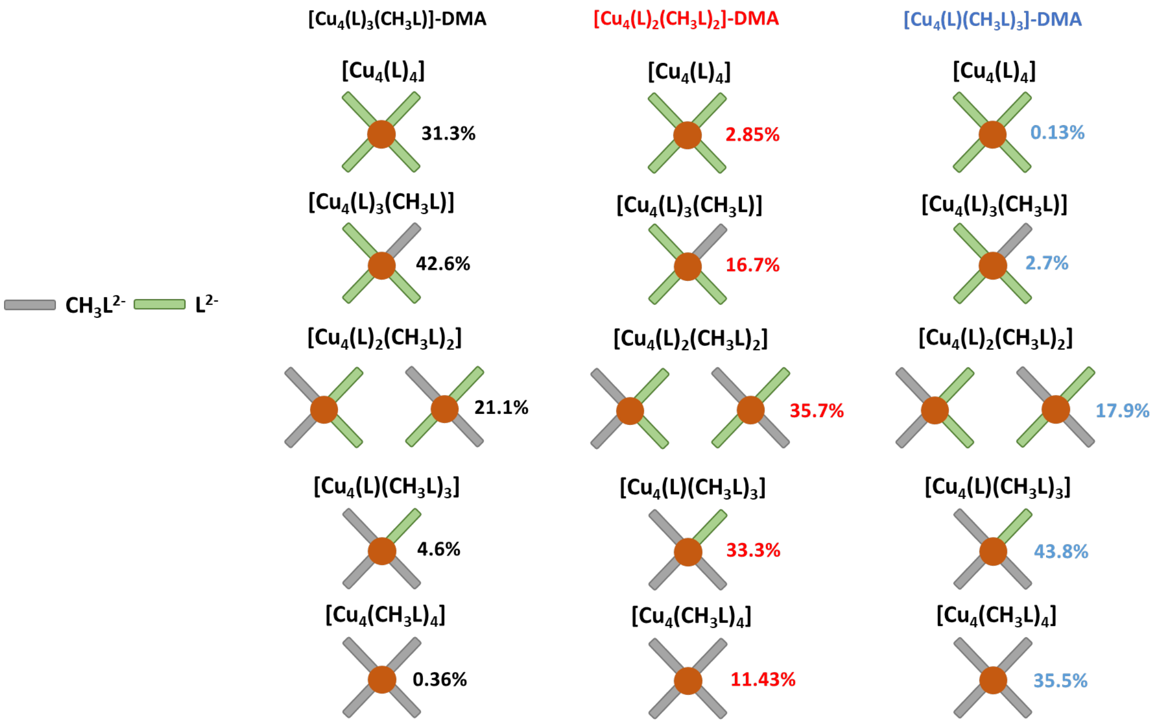

Figure S20. The statistical probability of the different possible compositions for each scrambled cage $[Cu_4(L)_{4-x}(CH_3L)_x]$-DMA (*x* = 1, 2, 3, represented by black, red and blue colours respectively), based on the occupancies derived from single crystal X-ray diffraction data. The schematic view of the cage is along the paddlewheel axis, with the grey and green rods representing the ligands CH_3L^{2-} and L^{2-} respectively. The rust sphere represent the copper paddlewheel. The probability for each composition based on the single crystal X-ray diffraction data is displayed underneath for the family.

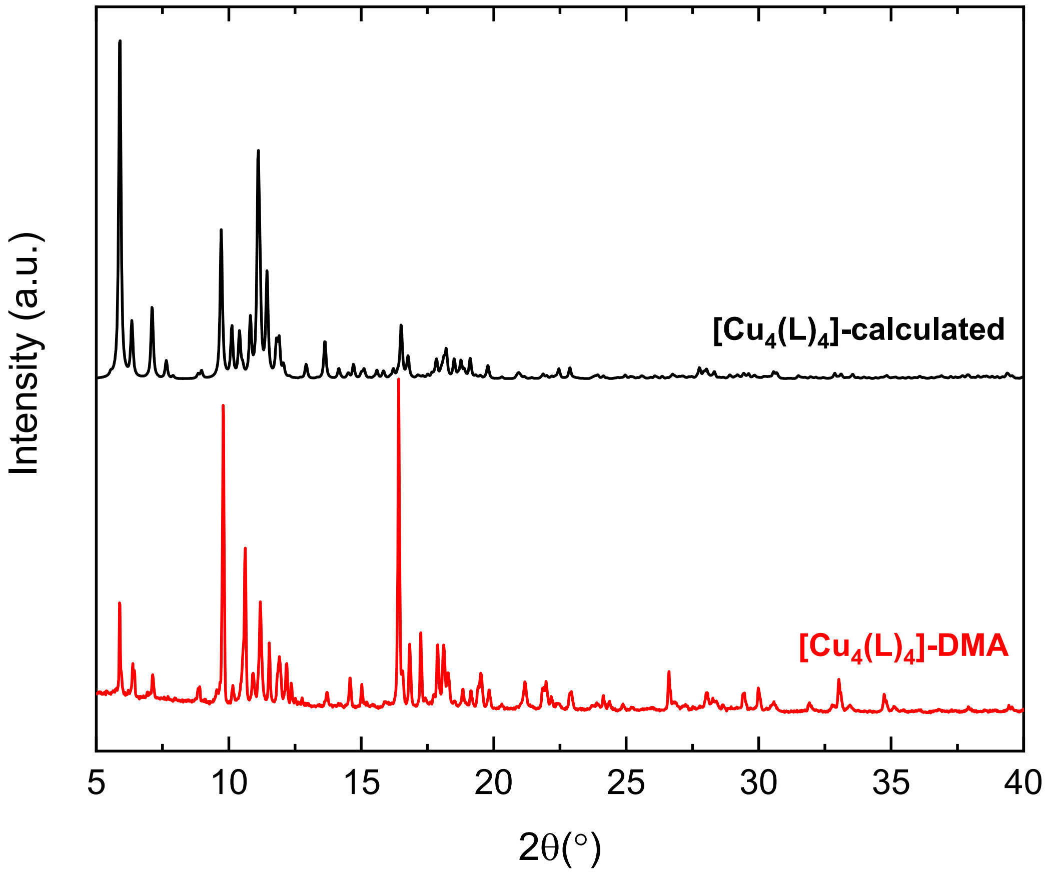

Figure S21. Powder X-ray diffractograms calculated for **$[Cu_4(L)_4]$-DMA** from .cif files (black) and collected on the sample of **$[Cu_4(L)_4]$-DMA** (red).

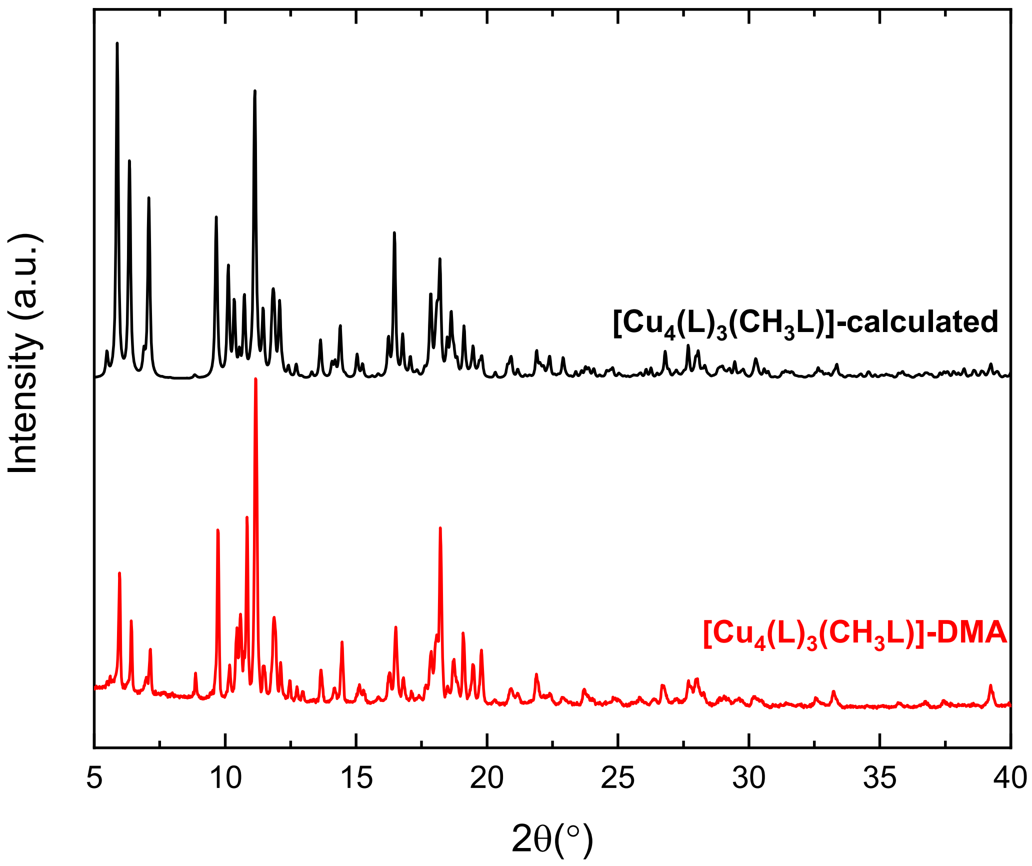

Figure S22. Powder X-ray diffractograms calculated for **[Cu_4(L)$_3$(CH_3L)]-DMA** from .cif files (black) and collected on the sample of **[Cu_4(L)$_3$(CH_3L)]-DMA** (red).

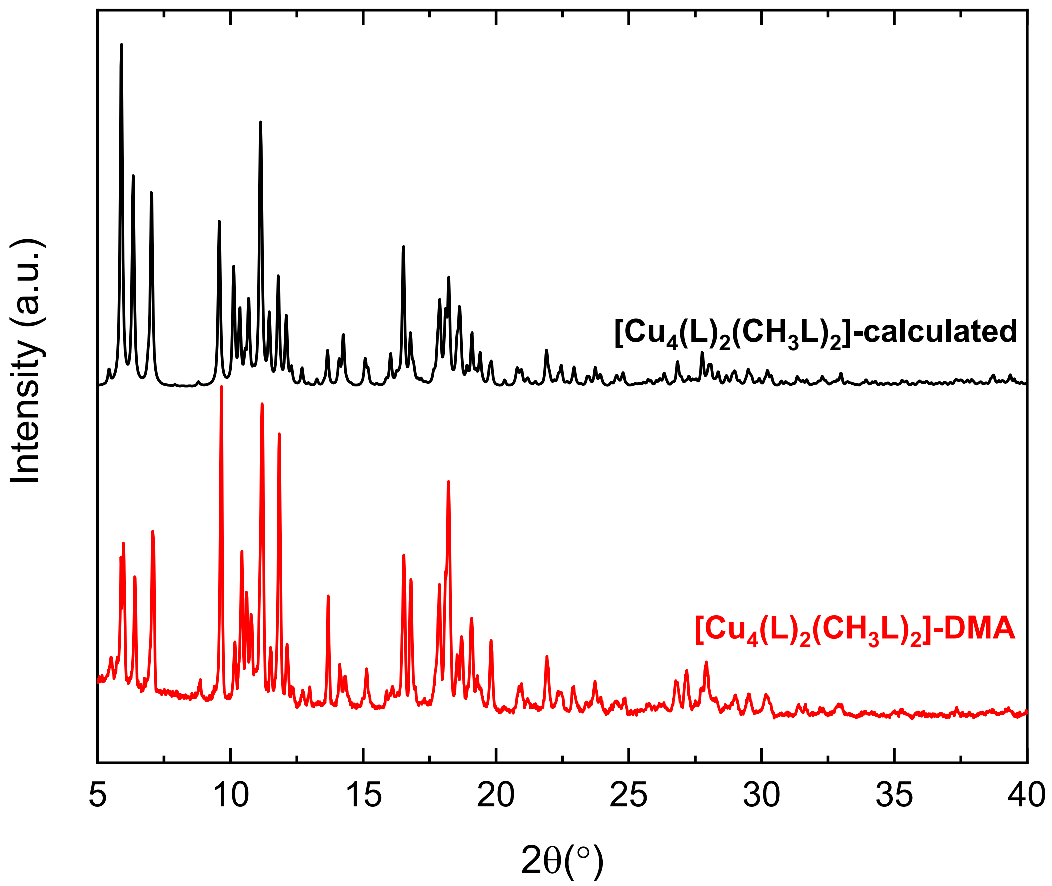

Figure S23. Powder X-ray diffractograms calculated for **[$Cu_4(L)_2(CH_3L)_2$]-DMA** from .cif files (black) and collected on the sample of **[$Cu_4(L)_2(CH_3L)_2$]-DMA** (red).

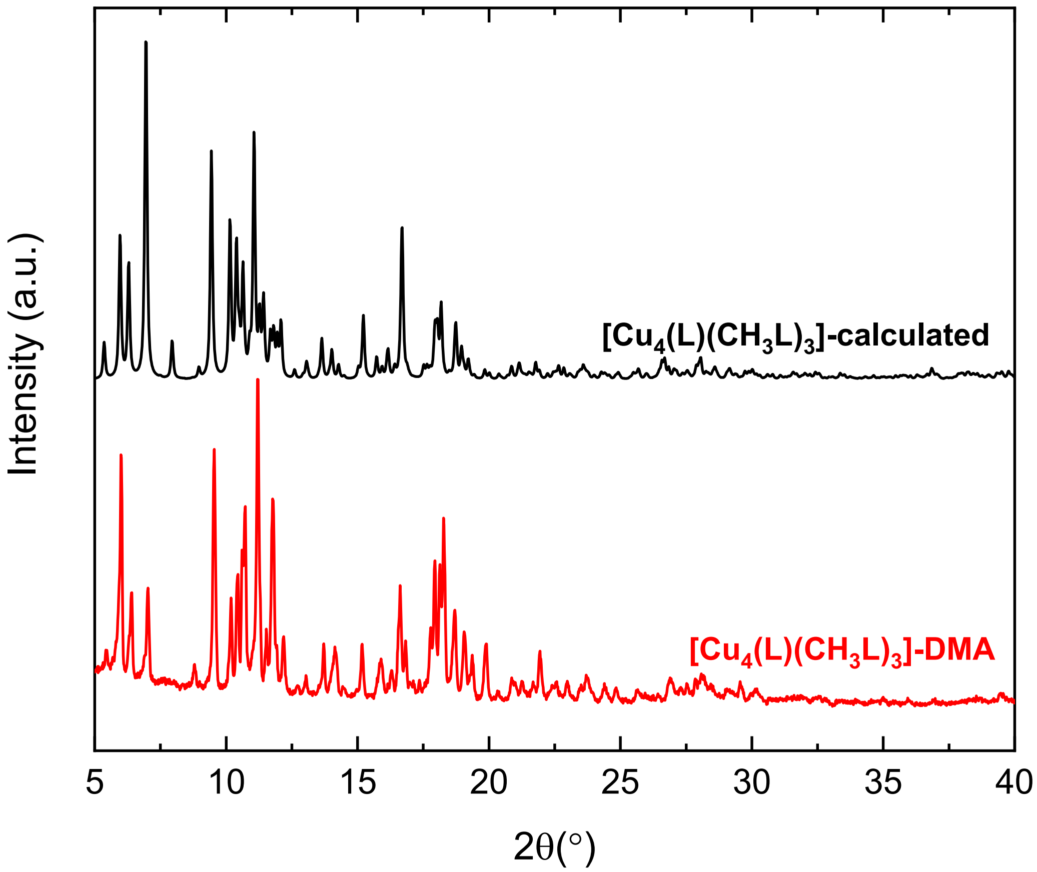

Figure S24. Powder X-ray diffractograms calculated for **[Cu_4(L)(CH_3L)$_3$]-DMA** from .cif files (black) and collected on the sample of **[Cu_4(L)(CH_3L)$_3$]-DMA** (red).

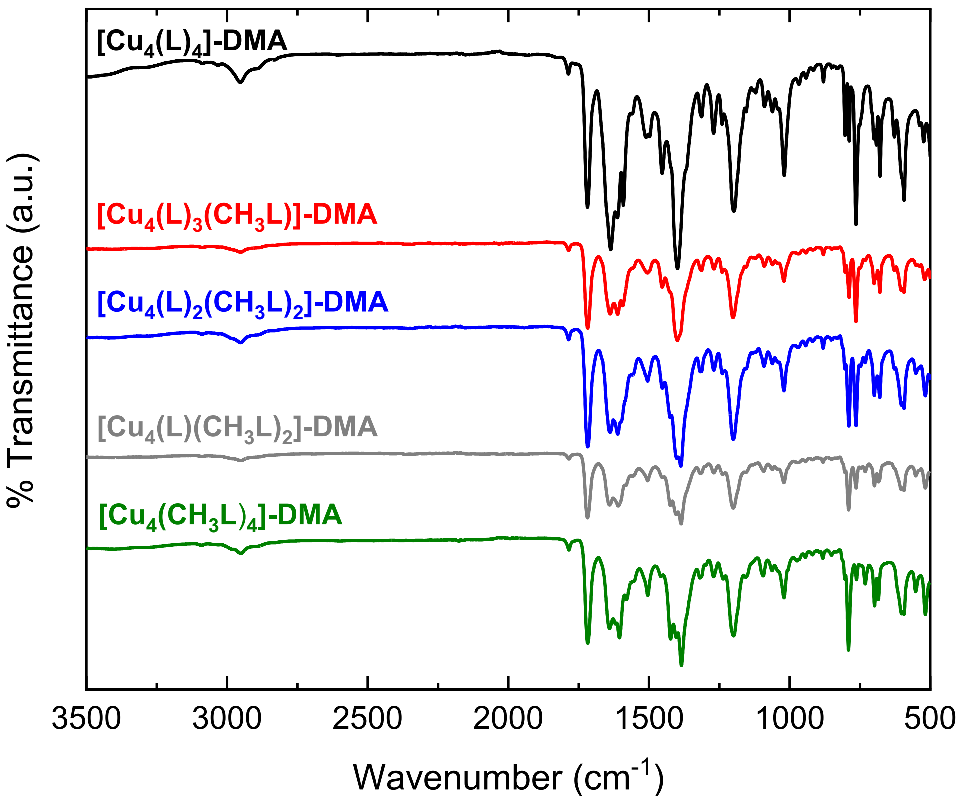

Figure S25. IR spectra of the as-synthesised cages for the family $[Cu_4(L)_{4-x}(CH_3L)_x]$-DMA (x = 0, 1, 2, 3, 4).

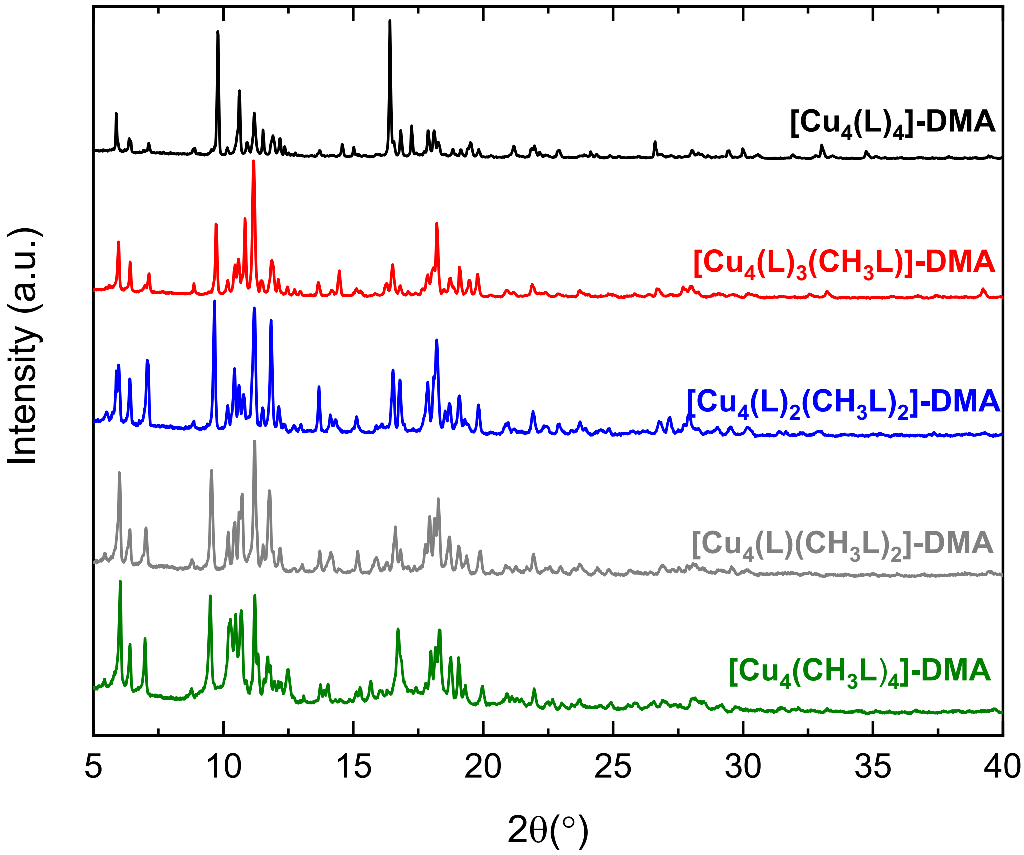

Figure S26. Powder X-ray diffractogram patterns of the as-synthesised family $[Cu_4(L)_{4-x}(CH_3L)_x]$-DMA (x=0, 1, 2, 3, 4).

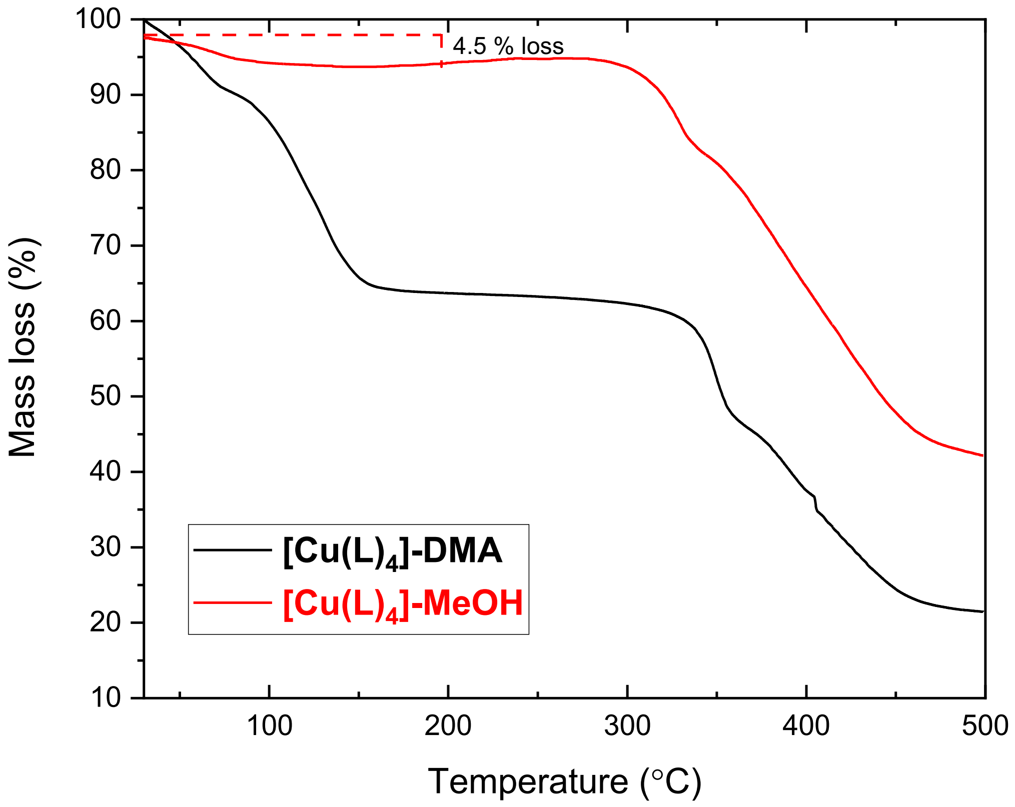

Figure S27. TGA trace of **[Cu_4(L)$_4$]-DMA** (black) and after solvent exchange with MeOH **[Cu_4(L)$_4$]-MeOH** (red). The mass loss in **[Cu_4(L)$_4$]-DMA** is divided into three main steps before decomposition, which are attributed to the loss of solvent from the pores up to approximately 150 °C, before loss of solvent attached to the paddlewheels and decomposition just above 330 °C. The trace for **[Cu_4(L)$_4$]-MeOH** shows mass loss of 7.2 % which is attributed to the loss of non-coordinated solvent before entering a plateau at around 250 °C. Based on the TGA trace of MeOH exchanged phase, the proposed formula for **[Cu_4(L)$_4$]-MeOH** is [Cu_4(L)$_4$(MeOH)$_2$(H_2O)$_2$]·5 MeOH.

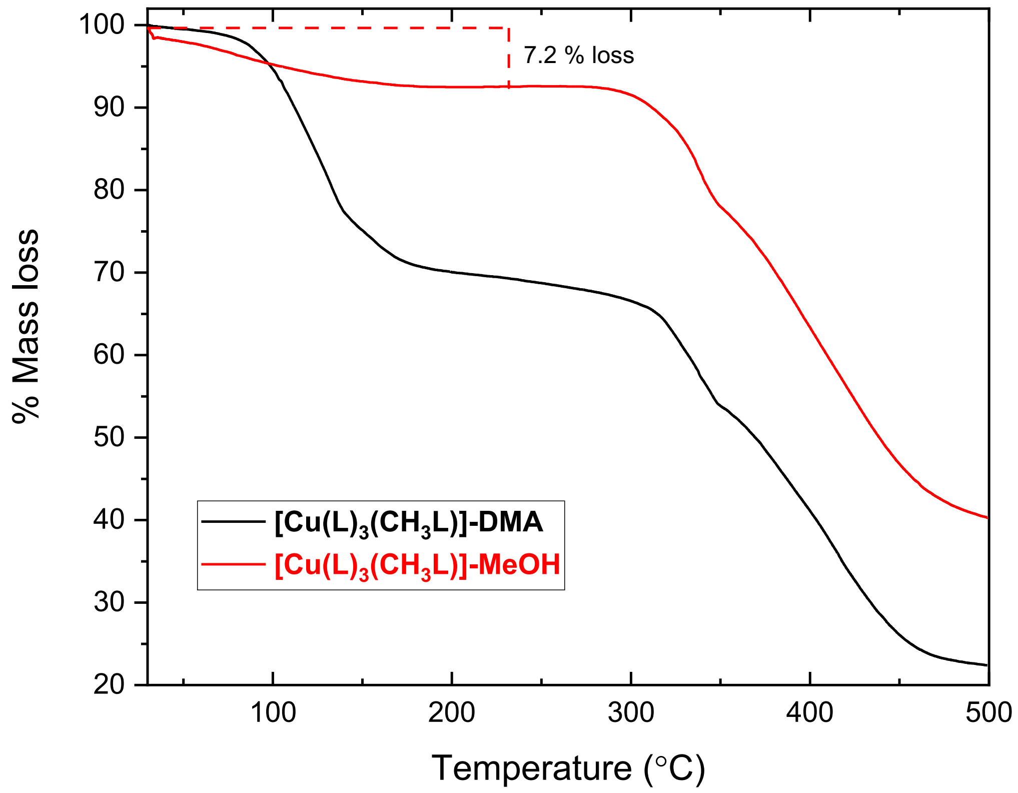

Figure S28. TGA trace of **$[Cu_4(L)_3(CH_3L)]$-DMA** (black) and after solvent exchange with MeOH **$[Cu_4(L)_3(CH_3L)]$-MeOH** (red). The mass loss in **$[Cu_4(L)_3(CH_3L)]$-DMA** is divided into three main steps before decomposition, which are attributed to the loss of solvent from the pores up to approximately 160 °C, before loss of solvent attached to the paddlewheels and decomposition just above 320 °C. The trace for **$[Cu_4(L)_3(CH_3L)]$-MeOH** shows mass loss of 7.2 % which is attributed to the loss of non-coordinated solvent before entering a plateau at around 250 °C. Based on the TGA trace of MeOH exchanged phase, the proposed formula for **$[Cu_4(L)_3(CH_3L)]$-MeOH** is $[Cu_4(L)_3(CH_3L)(MeOH)_2(H_2O)_2]\cdot 5$ MeOH.

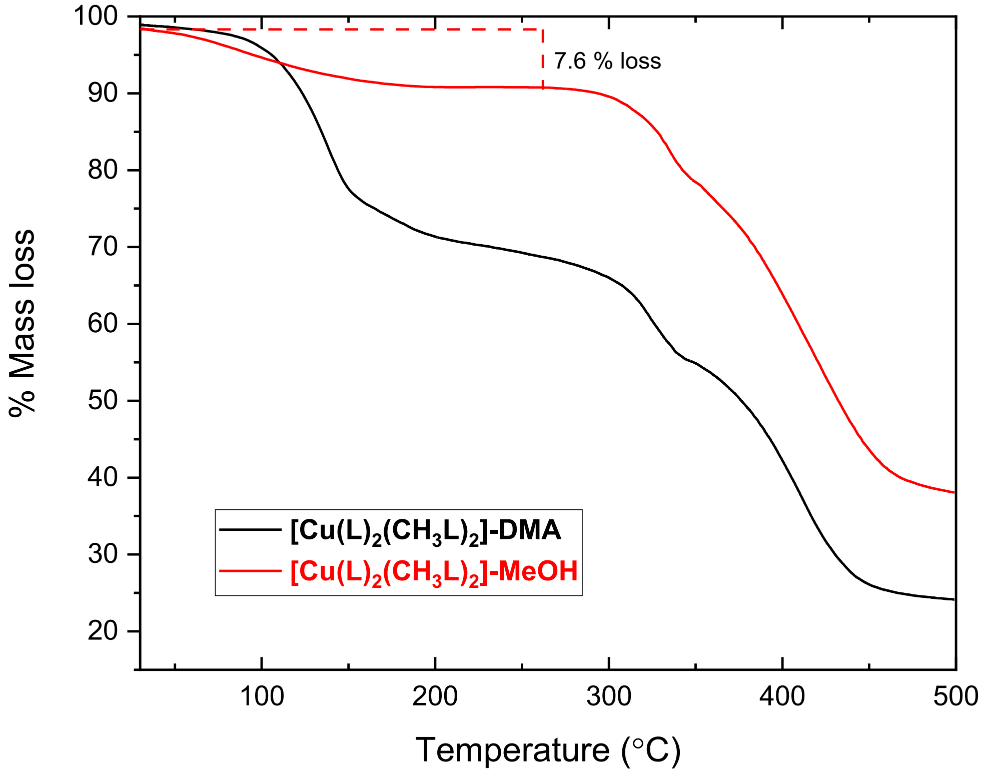

Figure S29. TGA trace of **$[Cu_4(L)_2(CH_3L)_2]$-DMA** (black) and after solvent exchange with MeOH **$[Cu_4(L)_2(CH_3L)_2]$-MeOH** (red). The mass loss in **$[Cu_4(L)_2(CH_3L)_2]$-DMA** is divided into three main steps before decomposition, which are attributed to the loss of solvent from the pores up to approximately 160 °C, before loss of solvent attached to the paddlewheels and decomposition just above 300 °C. The trace for **$[Cu_4(L)_2(CH_3L)_2]$-MeOH** shows mass loss of 7.6 % which is attributed to the loss of non-coordinated solvent before entering a plateau at around 250 °C. Based on the TGA trace of MeOH exchanged phase, the proposed formula for **$[Cu_4(L)_2(CH_3L)_2]$-MeOH** is $[Cu_4(L)_2(CH_3L)_2(MeOH)_2(H_2O)_2]\cdot 5$ MeOH.

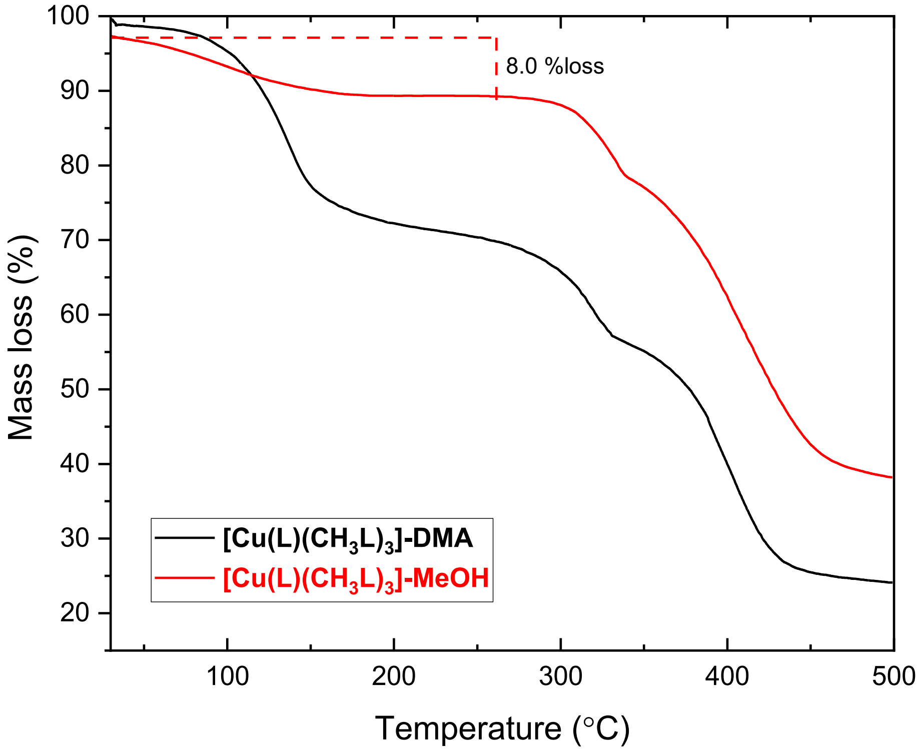

Figure S30. TGA trace of **[Cu_4(L)(CH_3L)$_3$]-DMA** (black) and after solvent exchange with MeOH **[Cu_4(L)(CH_3L)$_3$]-MeOH** (red). The mass loss in **[Cu_4(L)(CH_3L)$_3$]-DMA** is divided into three main steps before decomposition, which are attributed to the loss of solvent from the pores up to approximately 160 °C, before loss of solvent attached to the paddlewheels and decomposition just above 280 °C. The trace for **[Cu_4(L)(CH_3L)$_3$]-MeOH** shows mass loss of 8 % which is attributed to the loss of non-coordinated solvent before entering a plateau at around 260 °C. Based on the TGA trace of MeOH exchange phase, the proposed formula for **[Cu_4(L)(CH_3L)$_3$]-MeOH** is [Cu_4(L)(CH_3L)$_3$(MeOH)$_2$(H_2O)$_2$]·6 MeOH.

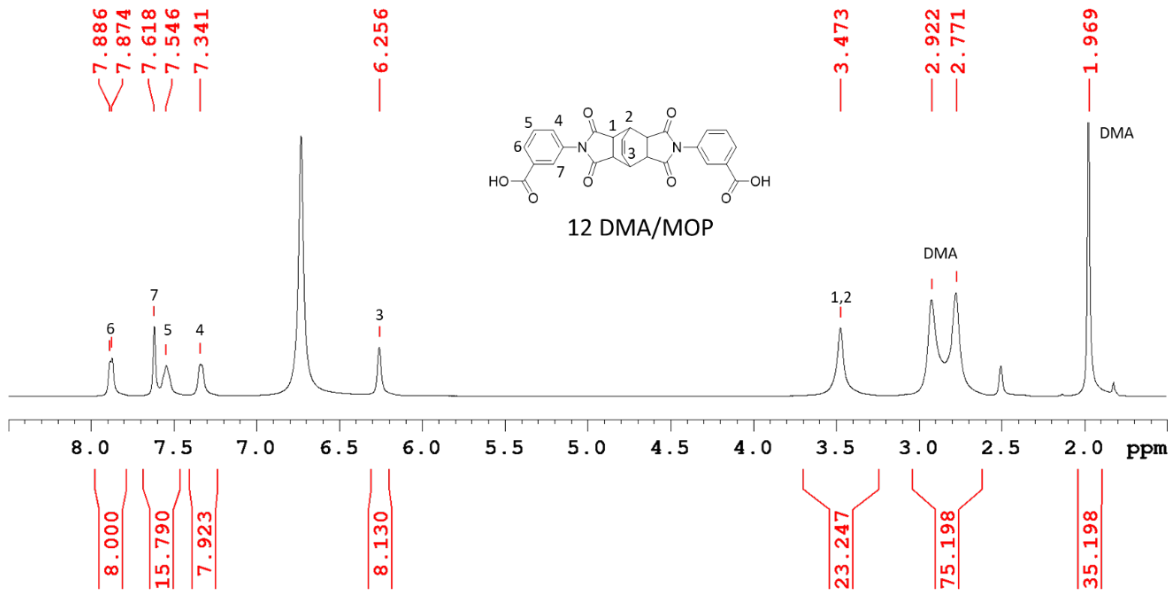

Figure S31. ^{1}H-NMR spectrum of the digestion in DMSO/DCl of **[$Cu_4(L)_4$]-DMA** showing the presence of 12 DMA in the structure

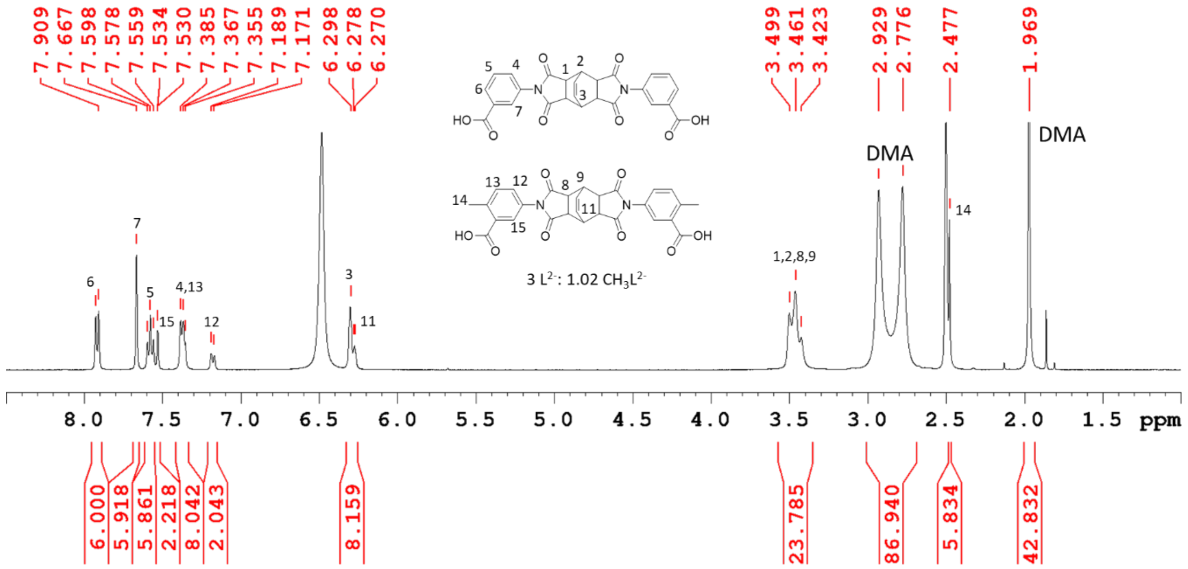

Figure S32. ^{1}H-NMR spectrum of the digestion in DMSO/DCl of **[$Cu_4(L)_3(CH_3L)$]-DMA** showing the presence of 14 DMA in the structure and a ratio of 3 : 1.02 for $L2^{2-}$:CH_3L^{2-}.

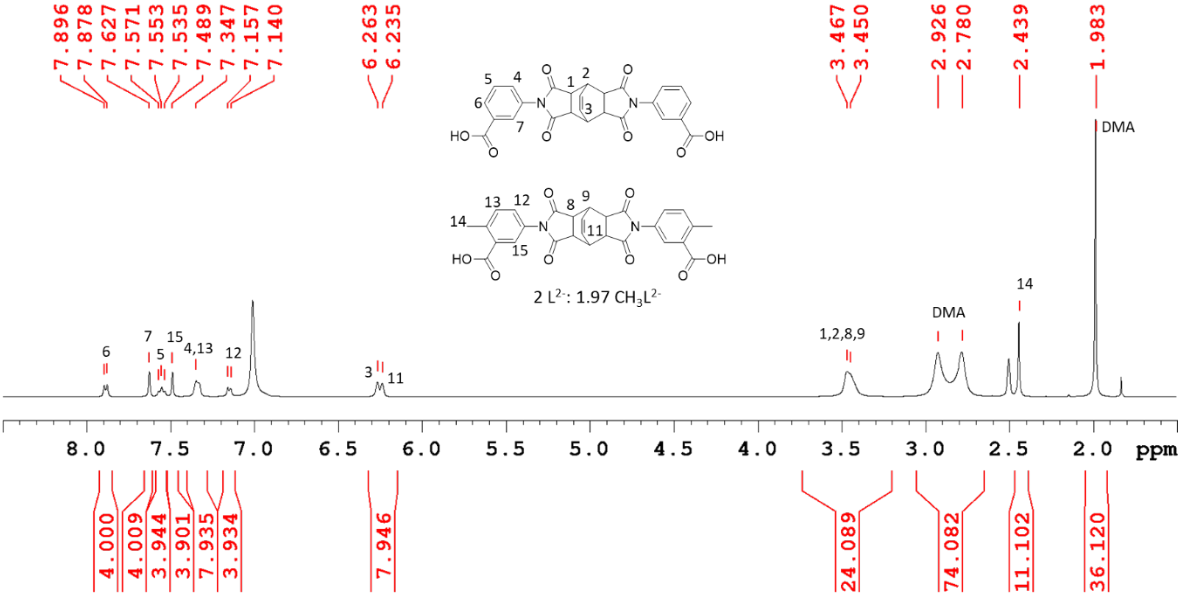

Figure S33. ^{1}H-NMR spectrum of the digestion in DMSO/DCl of **[$Cu_4(L)_2(CH_3L)_2$]-DMA** showing the presence of 12 DMA in the structure and a ratio of 2 : 1.97 for $L2^{2-}$:CH_3L^{2-}.

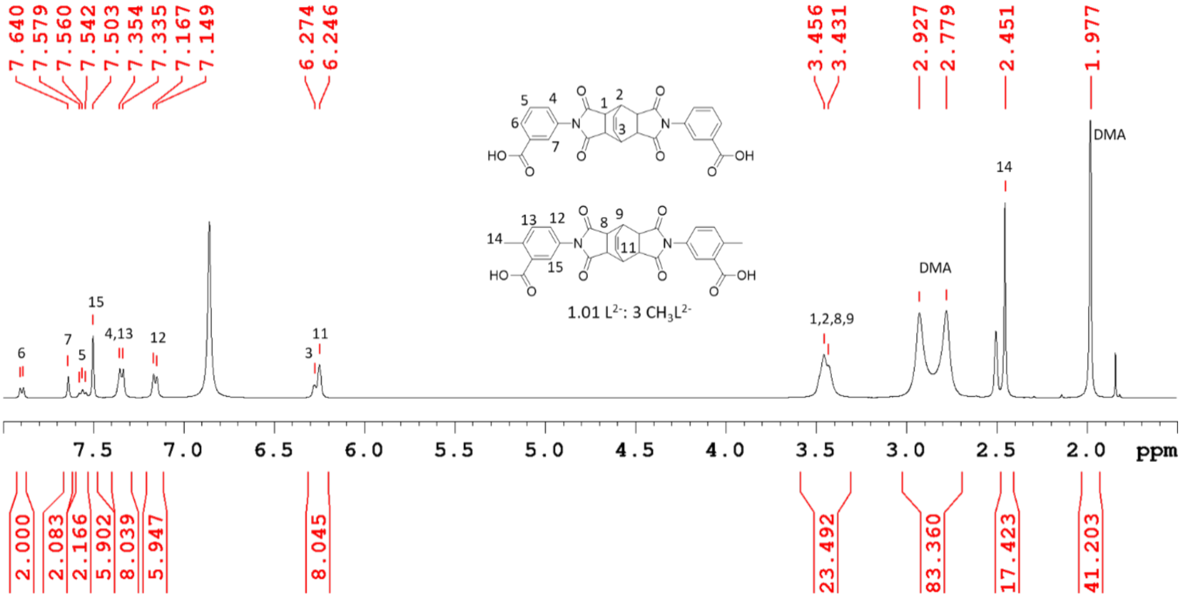

Figure S34. ^{1}H-NMR spectrum of the digestion in DMSO/DCl of **[$Cu_4(L)(CH_3L)_3$]-DMA** showing the presence of 14 DMA in the structure and a ratio of 1.01:3 for $L2^{2-}$:CH_3L^{2-}.

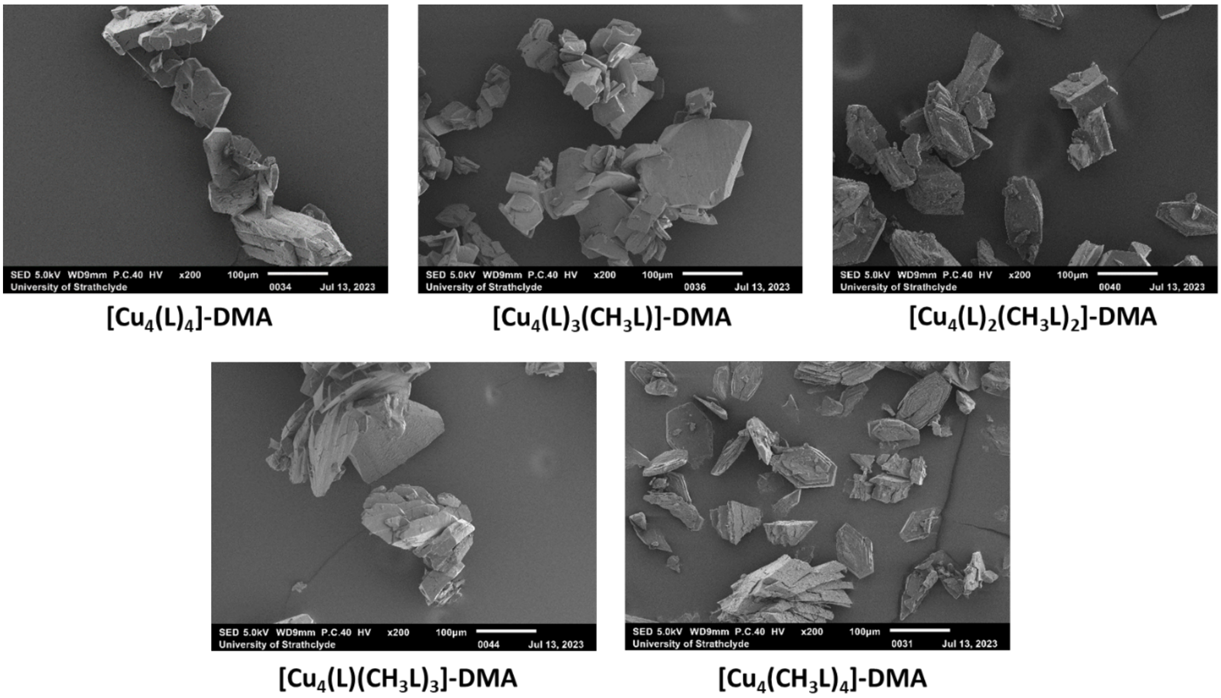

Figure S35. SEM images for the as-synthesised family [$Cu_4(L)_{4-x}(CH_3L)_x$]-DMA (x=0, 1, 2, 3, 4). Magnification used is x200.

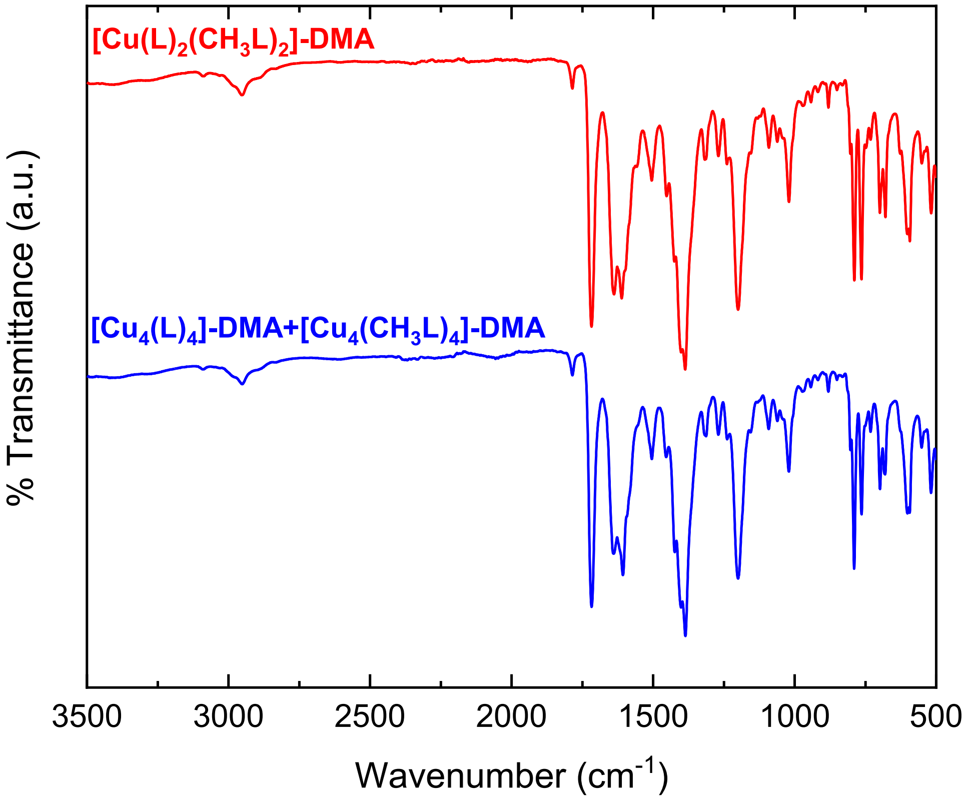

Figure S36. IR spectra of the scrambled cage **[$Cu_4(L)_2(CH_3L)_2$]-DMA** (red) and the physical mixture **[$Cu_4(L)_4$]-DMA + [$Cu_4(CH_3L)_4$]-DMA** (blue).

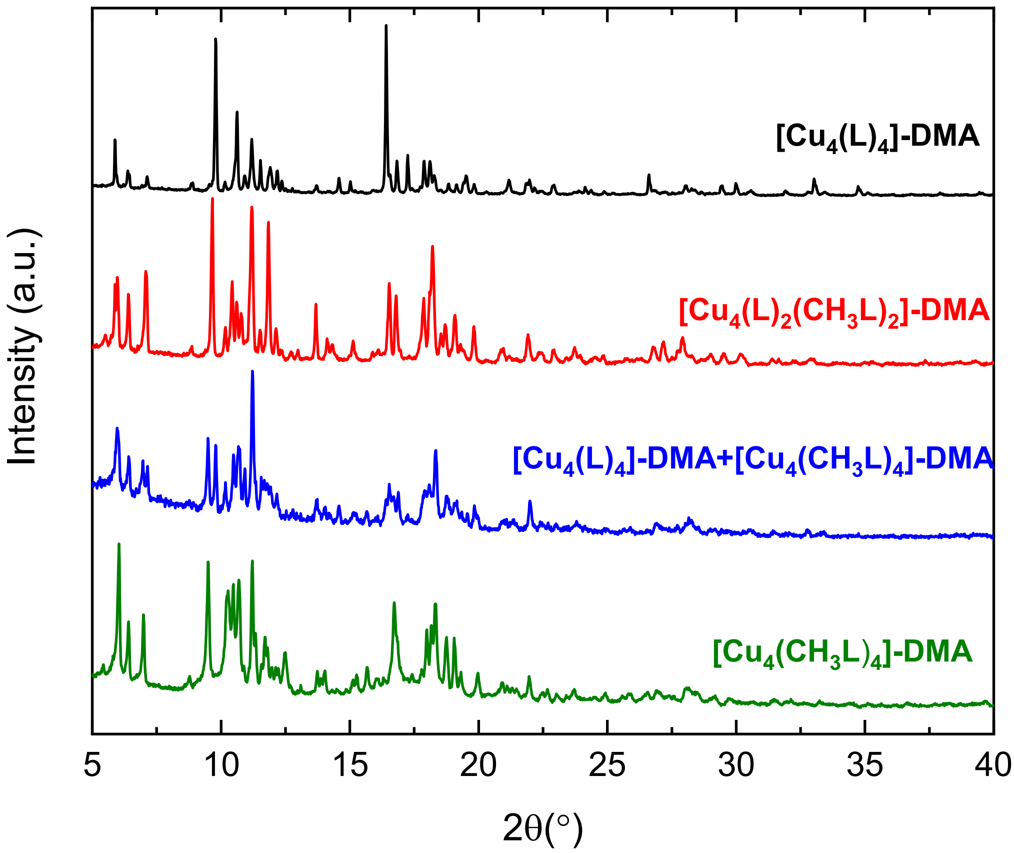

Figure S37. Full powder X-ray diffractograms of bulk homoleptic cage **[$Cu_4(L)_4$]-DMA** (black), the scrambled cage **[$Cu_4(L)_2(CH_3L)_2$]-DMA** (red), physical mixture **[$Cu_4(L)_4$]-DMA + [$Cu_4(CH_3L)_4$]-DMA** and bulk sample of homoleptic cage **[$Cu_4(CH_3L)_4$]-DMA** (olive).

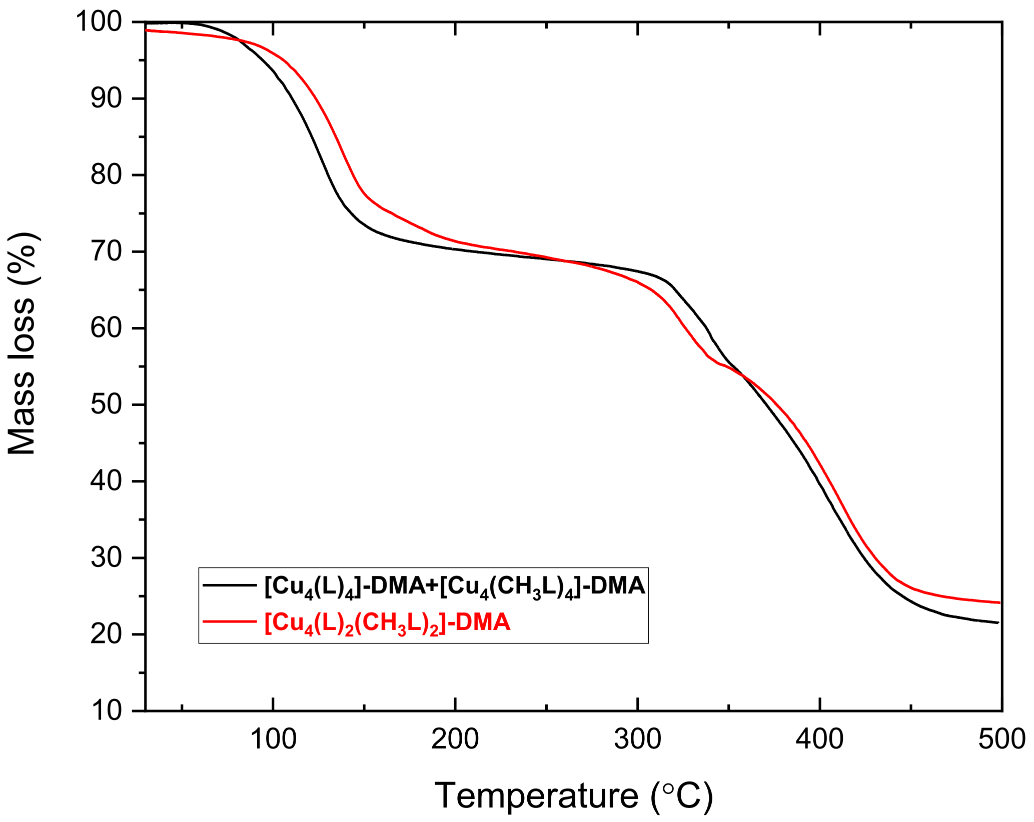

Figure S38. TGA trace of the scrambled cage **$[Cu_4(L)_2(CH_3L)_2]$-DMA** (red) compared to the TGA trace of the physical mixture **$[Cu_4(L)_4]$-DMA + $[Cu_4(L)_4]$-DMA** (black).

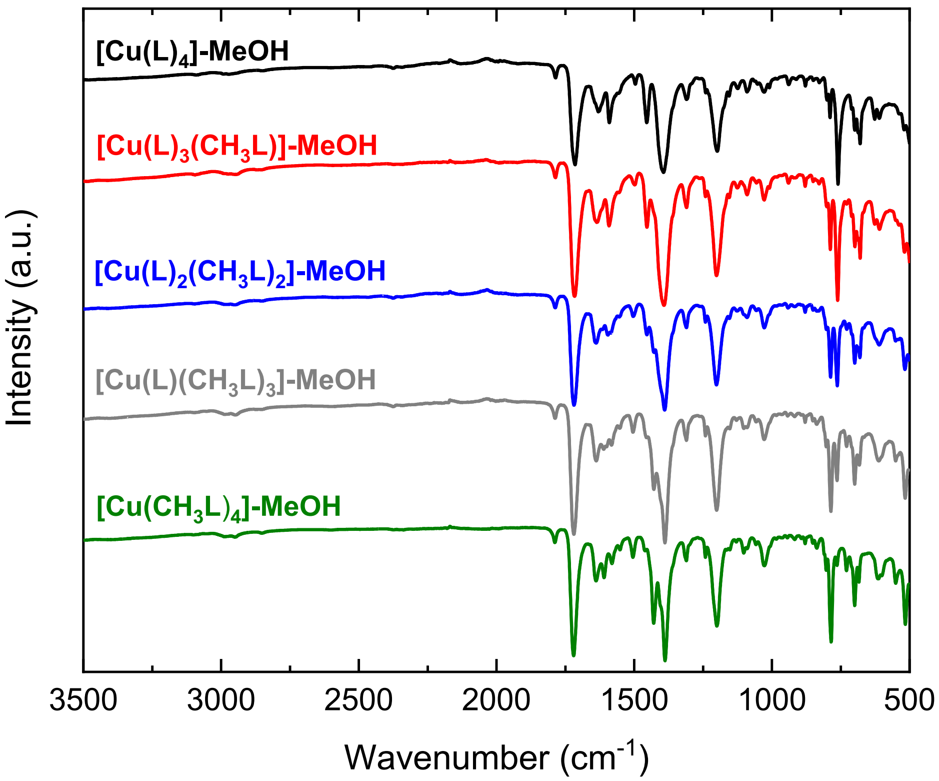

Figure S39. Infra-red spectra after solvent exchange with MeOH for the family $[Cu_4(L)_{4-x}(CH_3L)_x]$-MeOH (x=0, 1, 2, 3, 4).

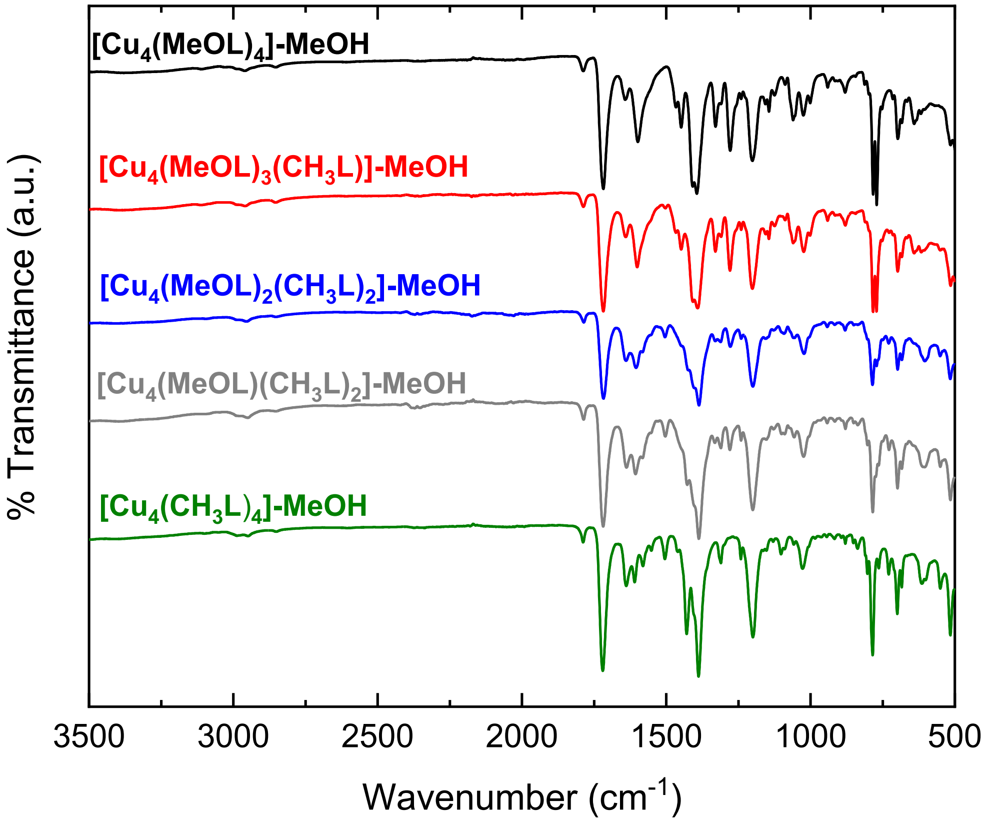

Figure S40. Infra-red spectra after solvent exchange with MeOH for the family $[Cu_4(MeOL)_{4-x}(CH_3L)_x]$-MeOH ($x$=0, 1, 2, 3, 4).

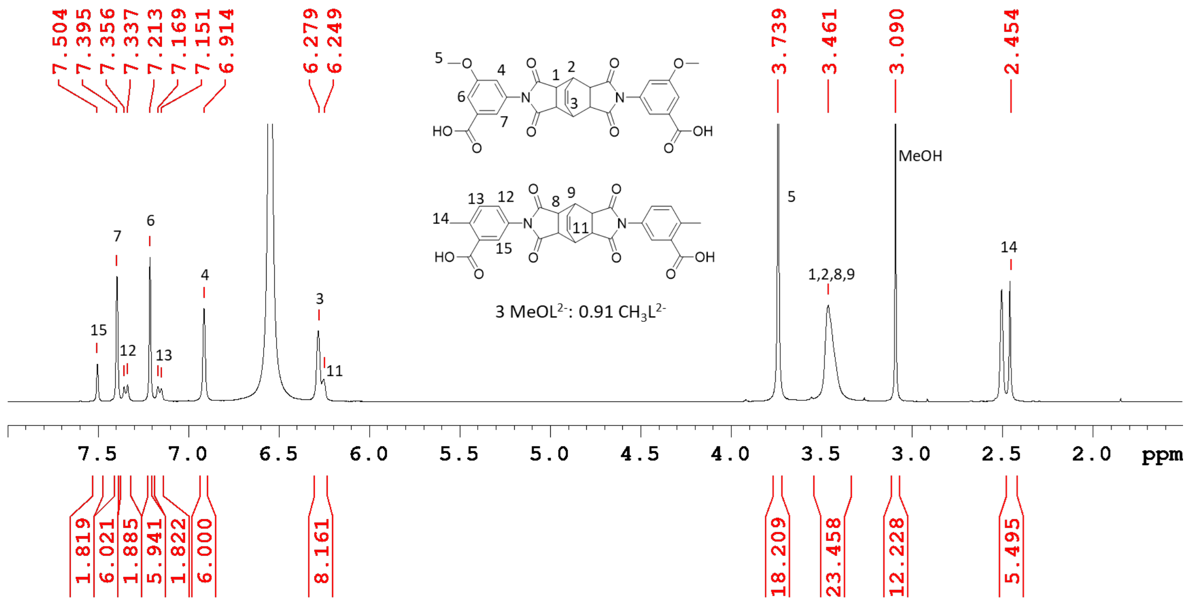

Figure S41. ^{1}H-NMR spectrum of the digestion in DMSO/DCl of **[Cu_4(MeOL)$_3$(CH_3L)]-MeOH** showing the presence of 4 MeOH in the structure and a ratio of 3 : 0.91 for MeOL2^{2-} : CH_3L^{2-}.

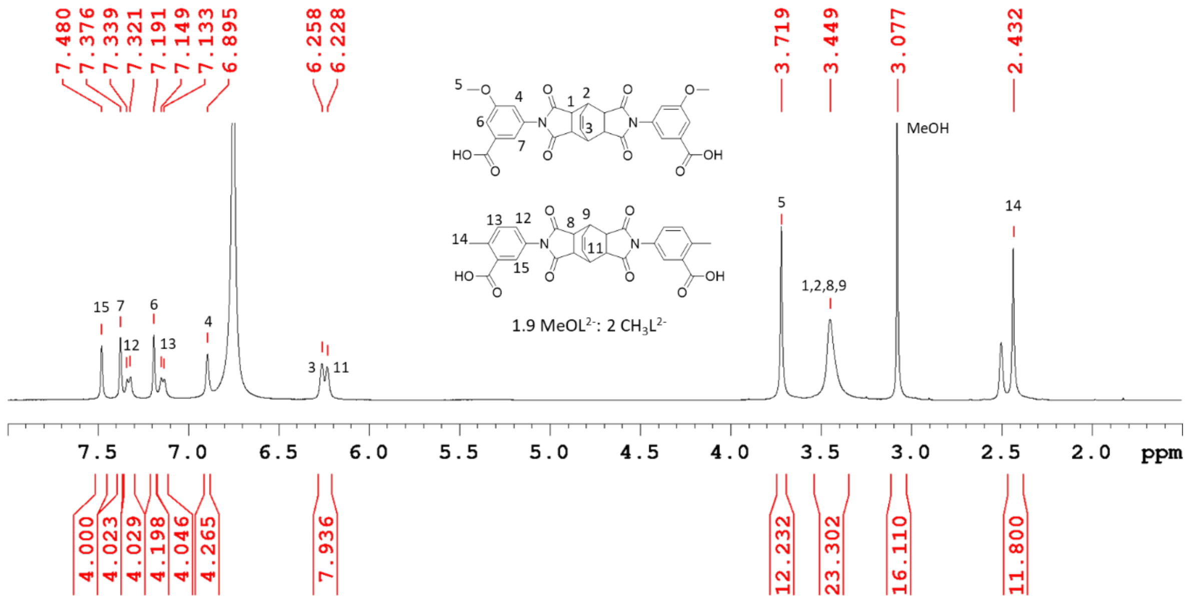

Figure S42. ^{1}H-NMR spectrum of the digestion in DMSO/DCl of **[Cu_4(MeOL)$_2$(CH_3L)$_2$]-MeOH** showing the presence of 5.3 MeOH in the structure and a ratio of 1.9 : 2 for MeOL2^{2-} : CH_3L^{2-}.

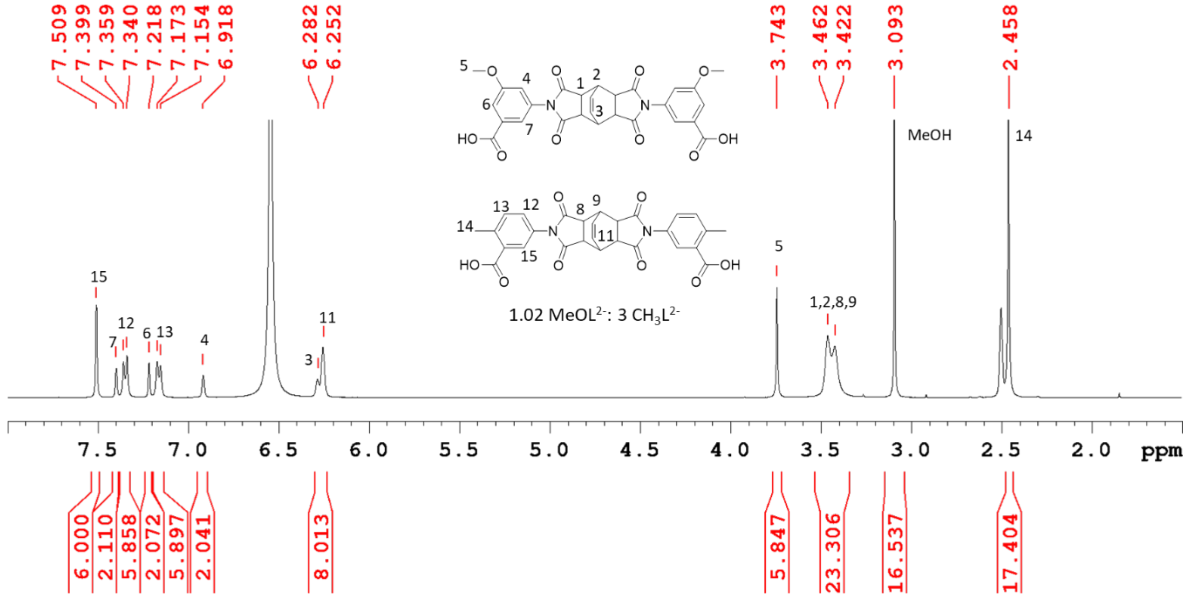

Figure S43. ^{1}H-NMR spectrum of the digestion in DMSO/DCl of **[Cu$_4$(MeOL)(CH$_3$L)$_3$]-MeOH** showing the presence of 5.5 MeOH in the structure and a ratio of 1.02 : 3 for MeOL2^{2-} : CH$_3$L^{2-}.

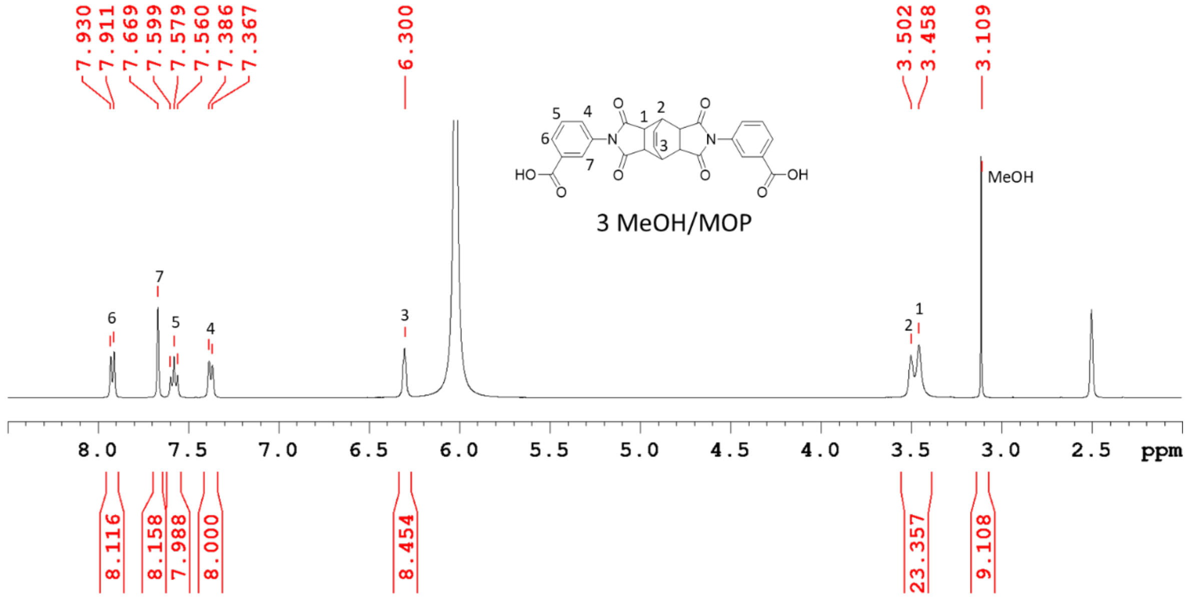

Figure S44. ^{1}H-NMR spectrum of the digestion in DMSO/DCl of **[Cu$_4$(L)$_4$]-MeOH** showing the presence of 3 MeOH in the structure.

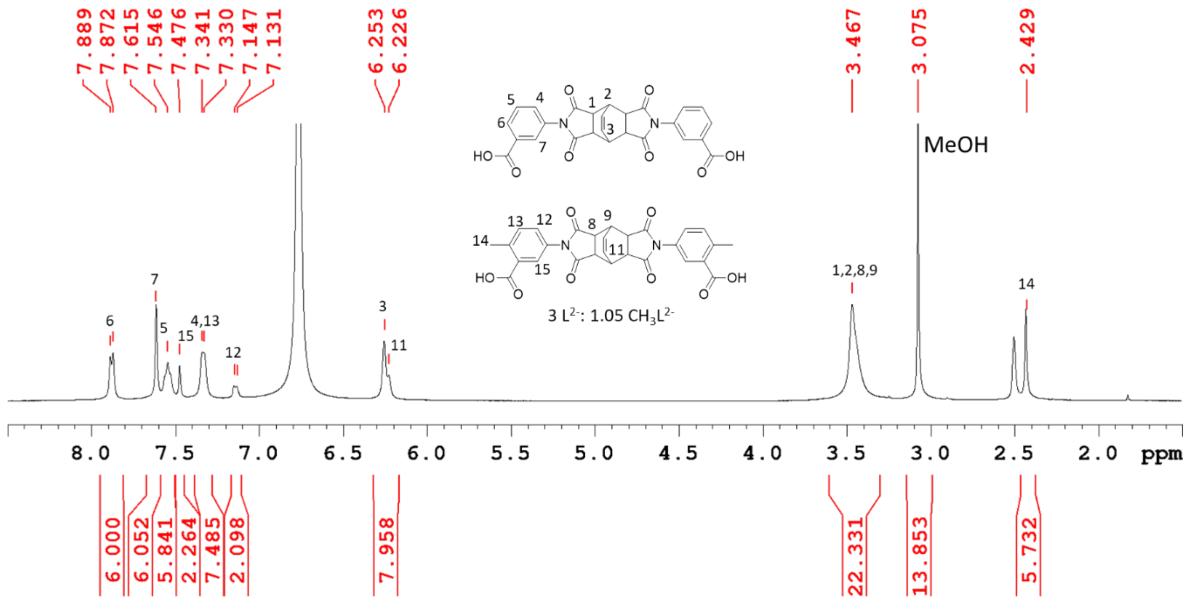

Figure S45. ^{1}H-NMR spectrum of the digestion in DMSO/DCl of **[$Cu_4(L)_3(CH_3L)$]-MeOH** showing the presence of 4.5 MeOH in the structure and a ratio of 3 : 1.05 for L2^{2-} : CH_3L^{2-}.

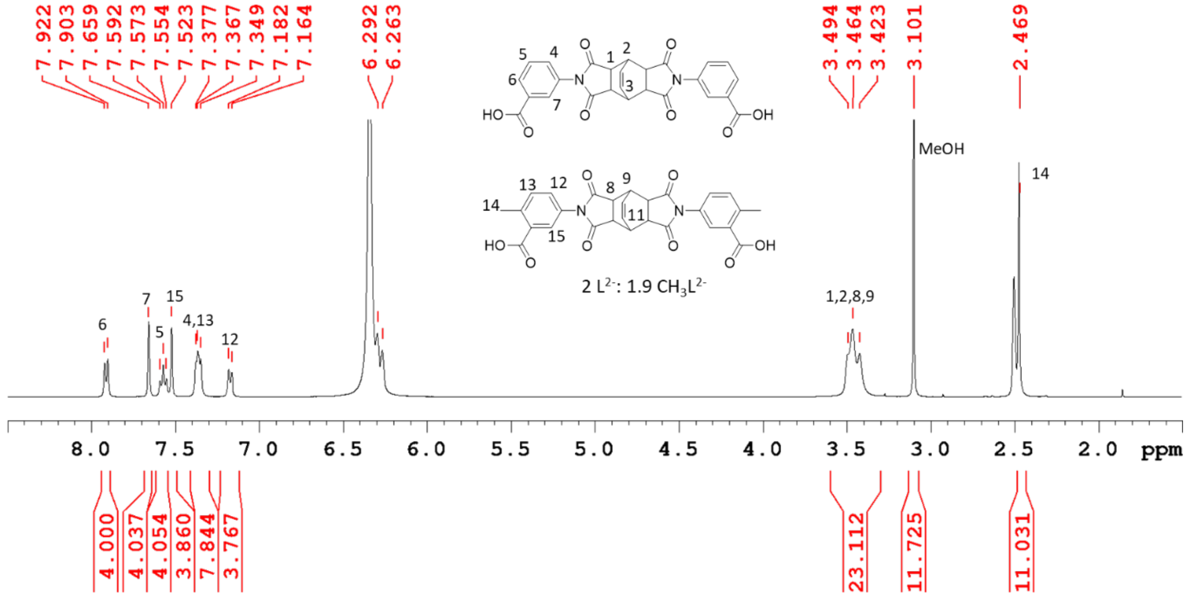

Figure S46. ^{1}H-NMR spectrum of the digestion in DMSO/DCl of **[$Cu_4(L)_2(CH_3L)_2$]-MeOH** showing the presence of 4 MeOH in the structure 2 : 1.9 for L2^{2-} : CH_3L^{2-}.

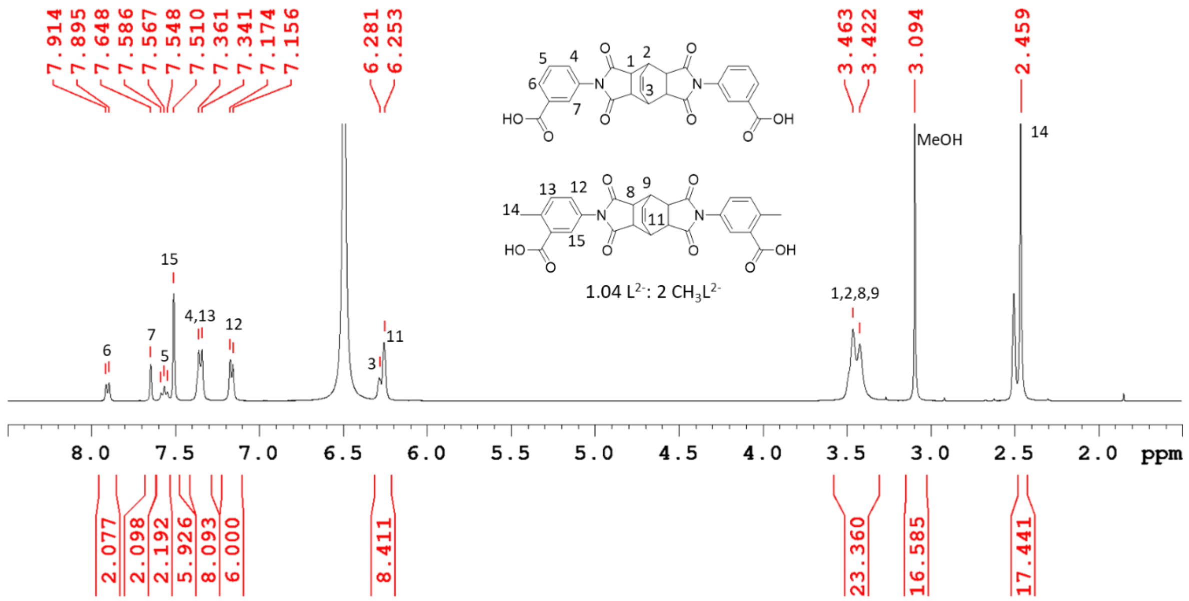

Figure S47. ^{1}H-NMR spectrum of the digestion in DMSO/DCl of **$[Cu_4(L)(CH_3L)_3]$-MeOH** showing the presence of 5.5 MeOH in the structure 1.04 : 2 for $L2^{2-}$: CH_3L^{2-}.

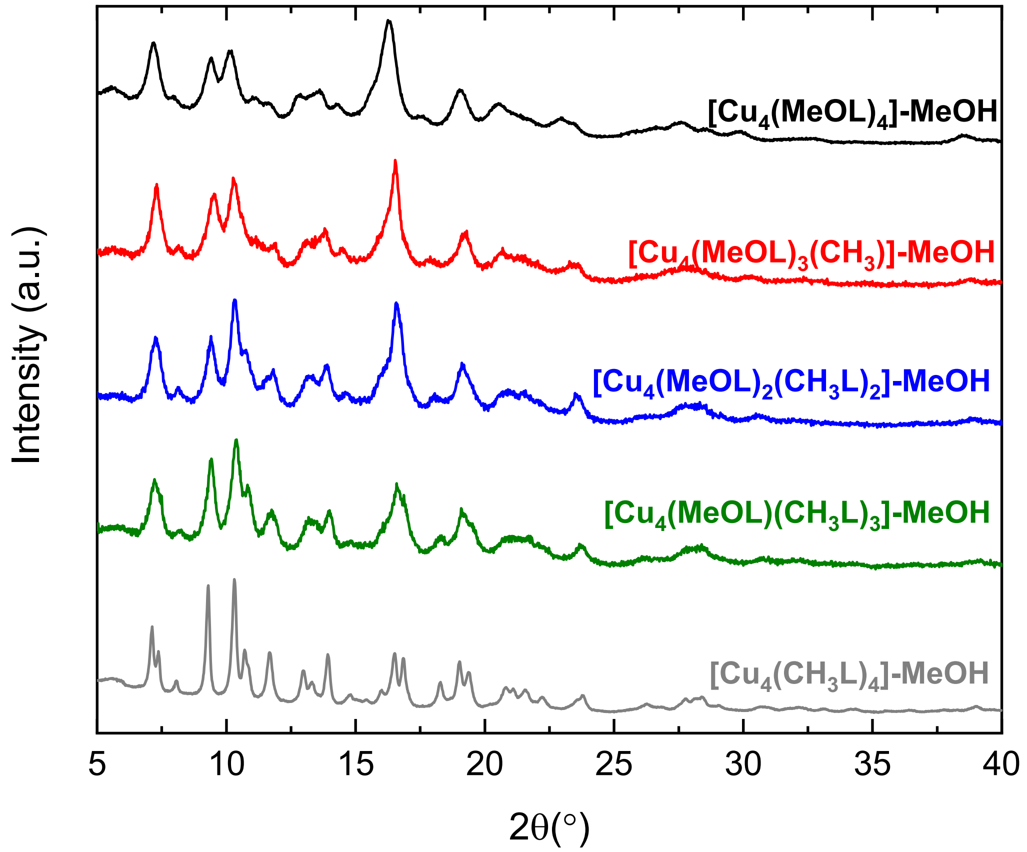

Figure S48. Powder X-ray diffractograms after solvent exchange with MeOH for the family $[Cu_4(MeOL)_{4-x}(CH_3L)_x]$-MeOH ($x$=0, 1, 2, 3, 4).

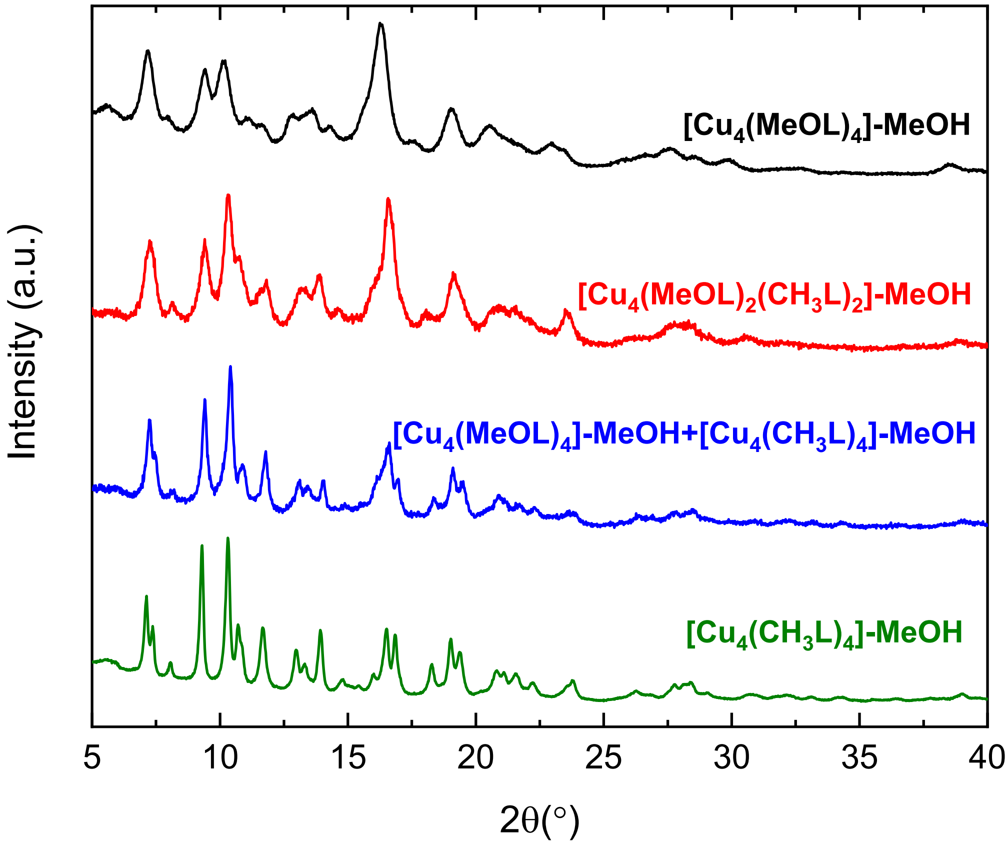

Figure S49. Powder X-ray diffractograms after solvent exchange with MeOH for the homoleptic cages **[$Cu_4(MeOL)_4$]-MeOH** (black) and **[$Cu_4(CH_3L)_4$]-MeOH** (olive) compared to the scrambled cage **[$Cu_4(MeOL)_2(CH_3L)_2$]-MeOH** (red) and the physical mixture **[$Cu_4(MeOL)_4$]-MeOH + [$Cu_4(CH_3L)_4$]-MeOH** (blue).

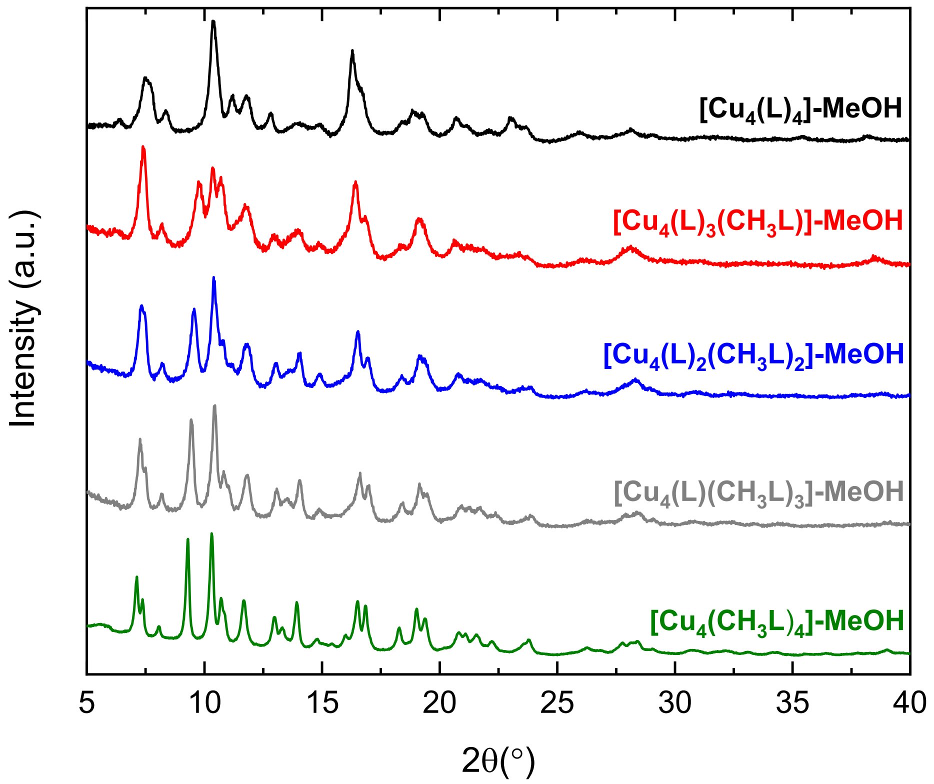

Figure S50. Powder X-ray diffractograms after solvent exchange with MeOH for the family $[Cu_4(L)_{4-x}(CH_3L)_x]$-MeOH (x=0, 1, 2, 3, 4).

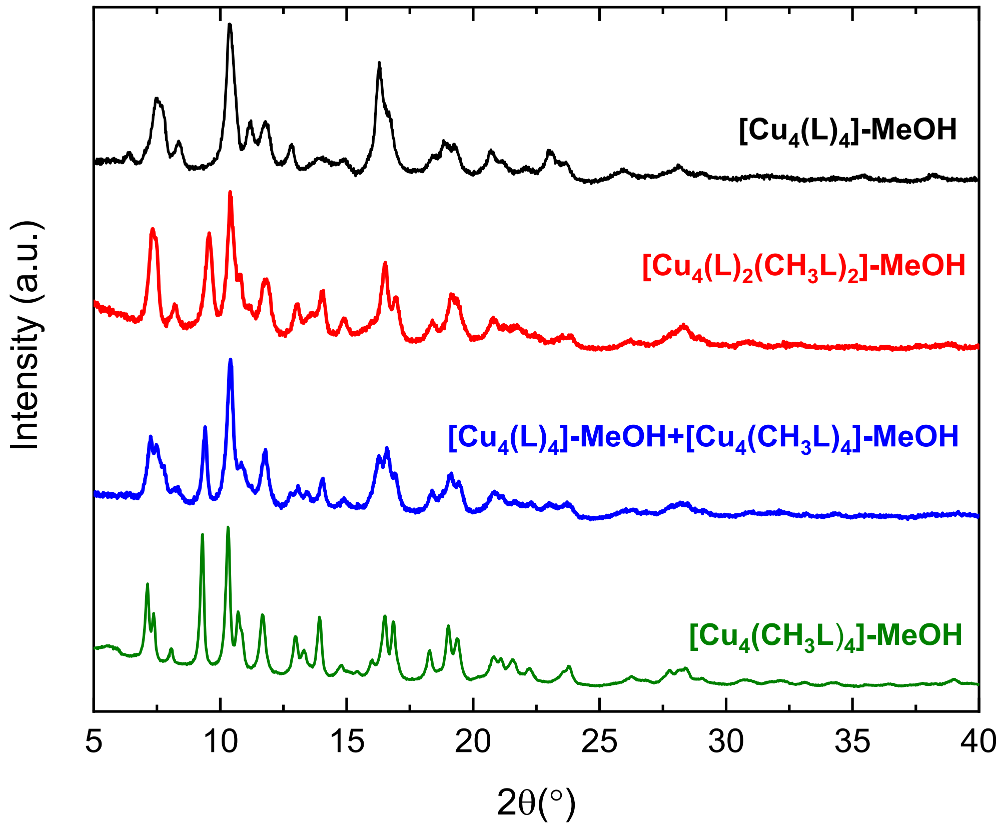

Figure S51. Powder X-ray diffractograms after solvent exchange with MeOH for the homoleptic cages **[$Cu_4(L)_4$]-MeOH** (black) and **[$Cu_4(CH_3L)_4$]-MeOH** (olive) compared to the scrambled cage **[$Cu_4(L)_2(CH_3L)_2$]-MeOH** (red) and the physical mixture **[$Cu_4(L)_4$]-MeOH + [$Cu_4(CH_3L)_4$]-MeOH** (blue).

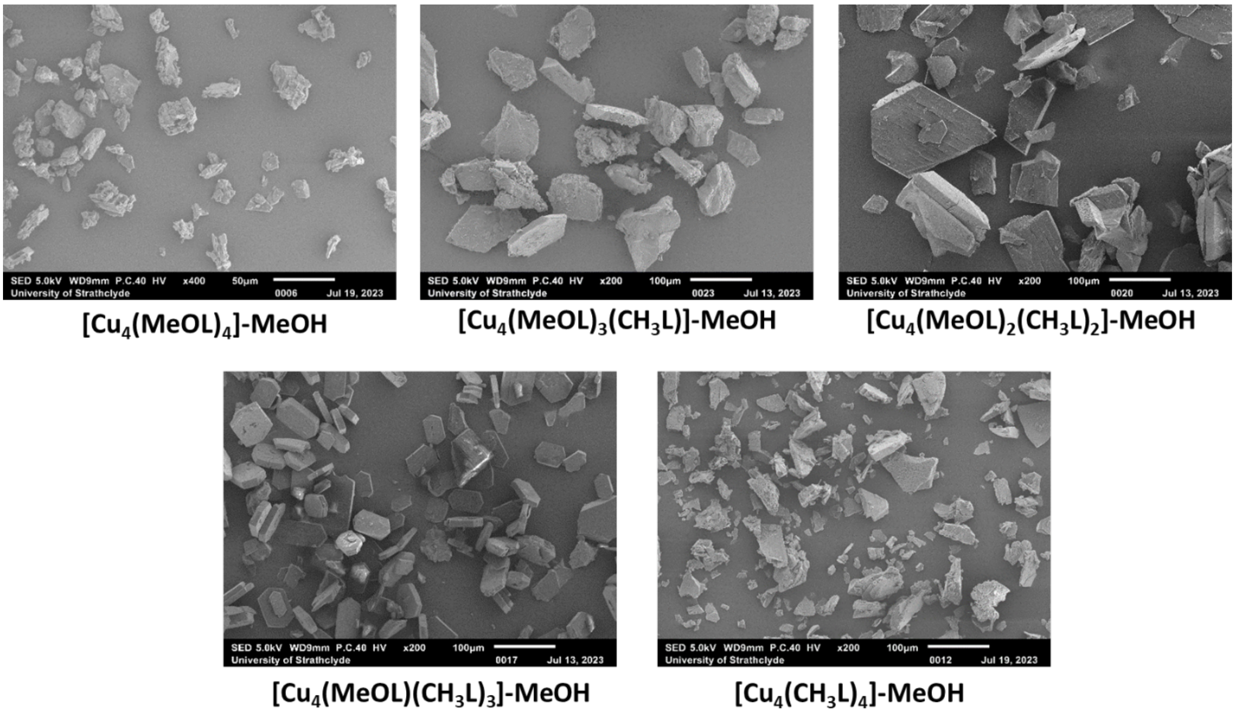

Figure S52. SEM images for the family [Cu$_4$(MeOL)$_{4-x}$(CH$_3$L)$_x$]-MeOH (x=0, 1, 2, 3, 4). Magnification used is x200.

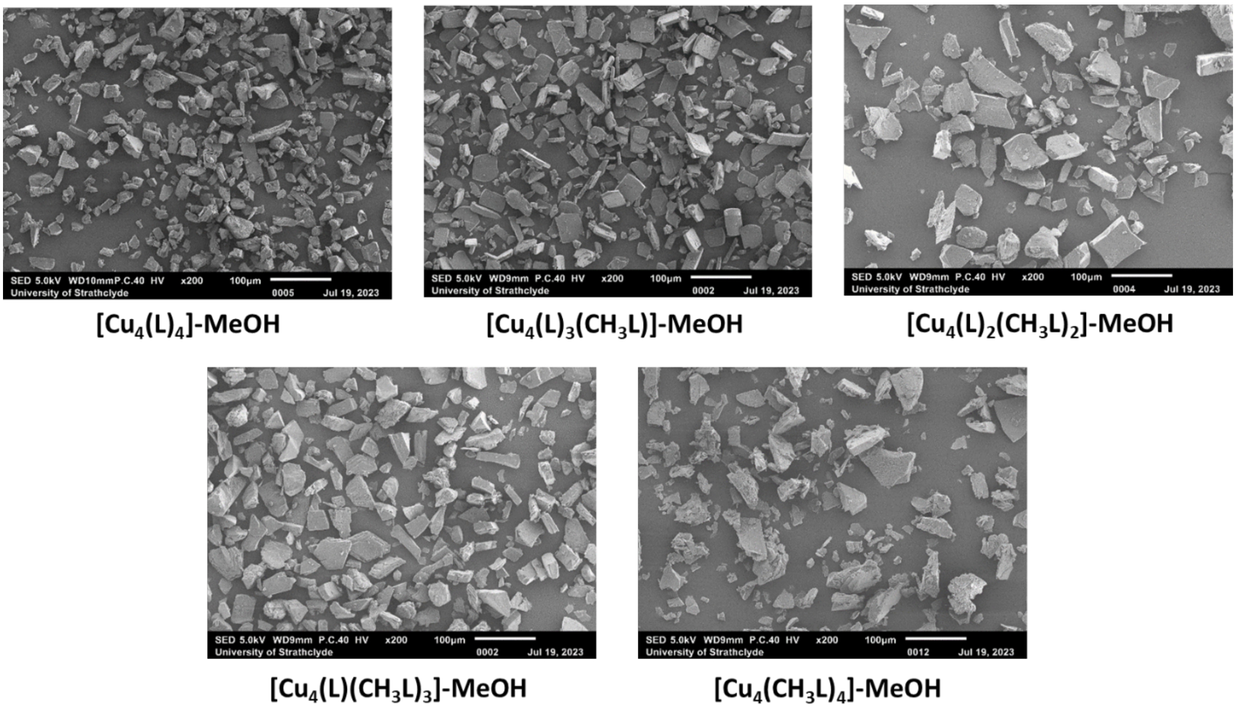

Figure S53. SEM images for the family [Cu$_4$(L)$_{4-x}$(CH$_3$L)$_x$]-MeOH (x=0, 1, 2, 3, 4). Magnification used is x200.

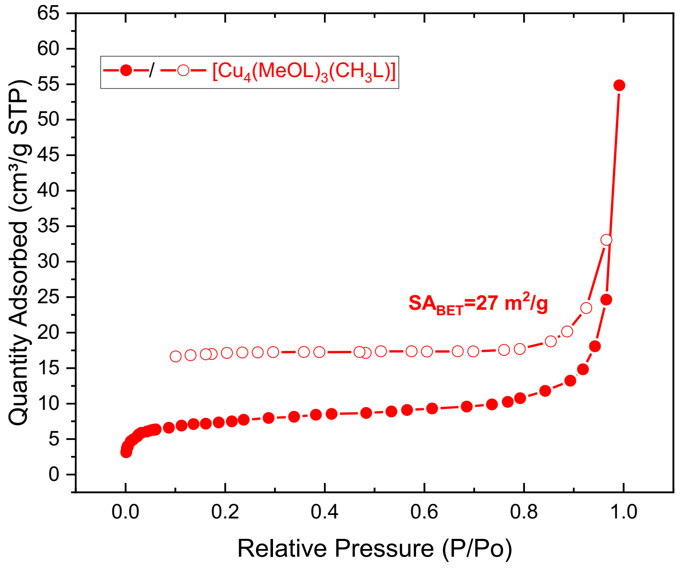

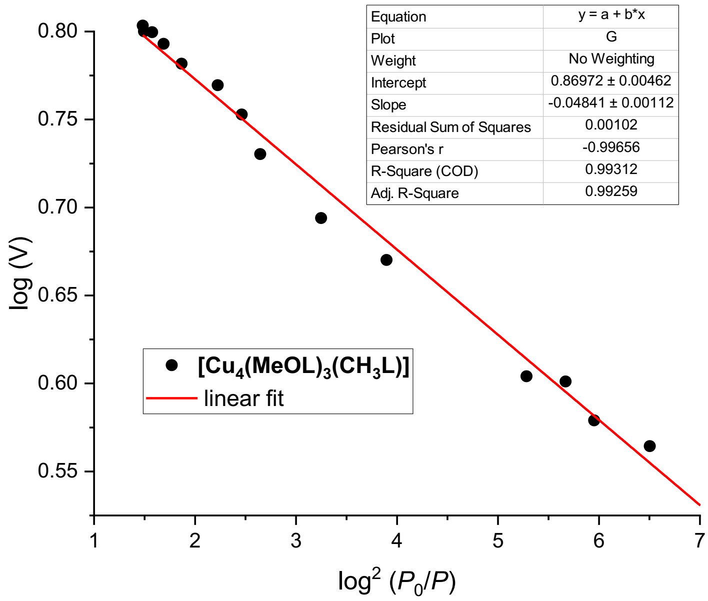

Figure S54. (top) N_2 isotherm at 77 K for **[$Cu_4(MeOL)_3(CH_3L)$]** and its calculated BET surface area. (bottom) Linear fit for Dubinin-Radishkevich approximation for the calculation of microporosity.

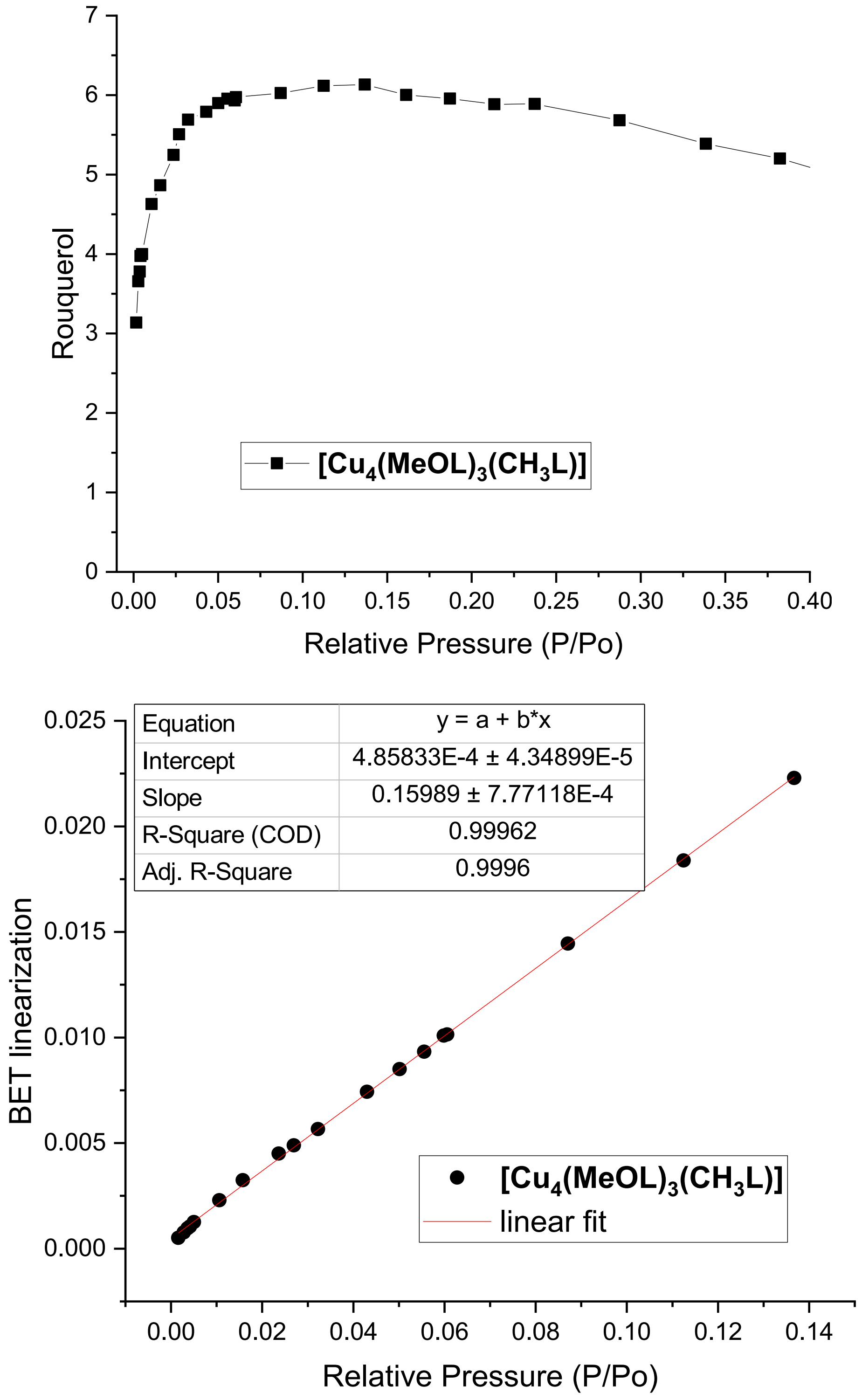

Figure S55. (top) Rouquerol graph calculated from isotherm. (bottom) Linear fit used for BET surface area calculation.

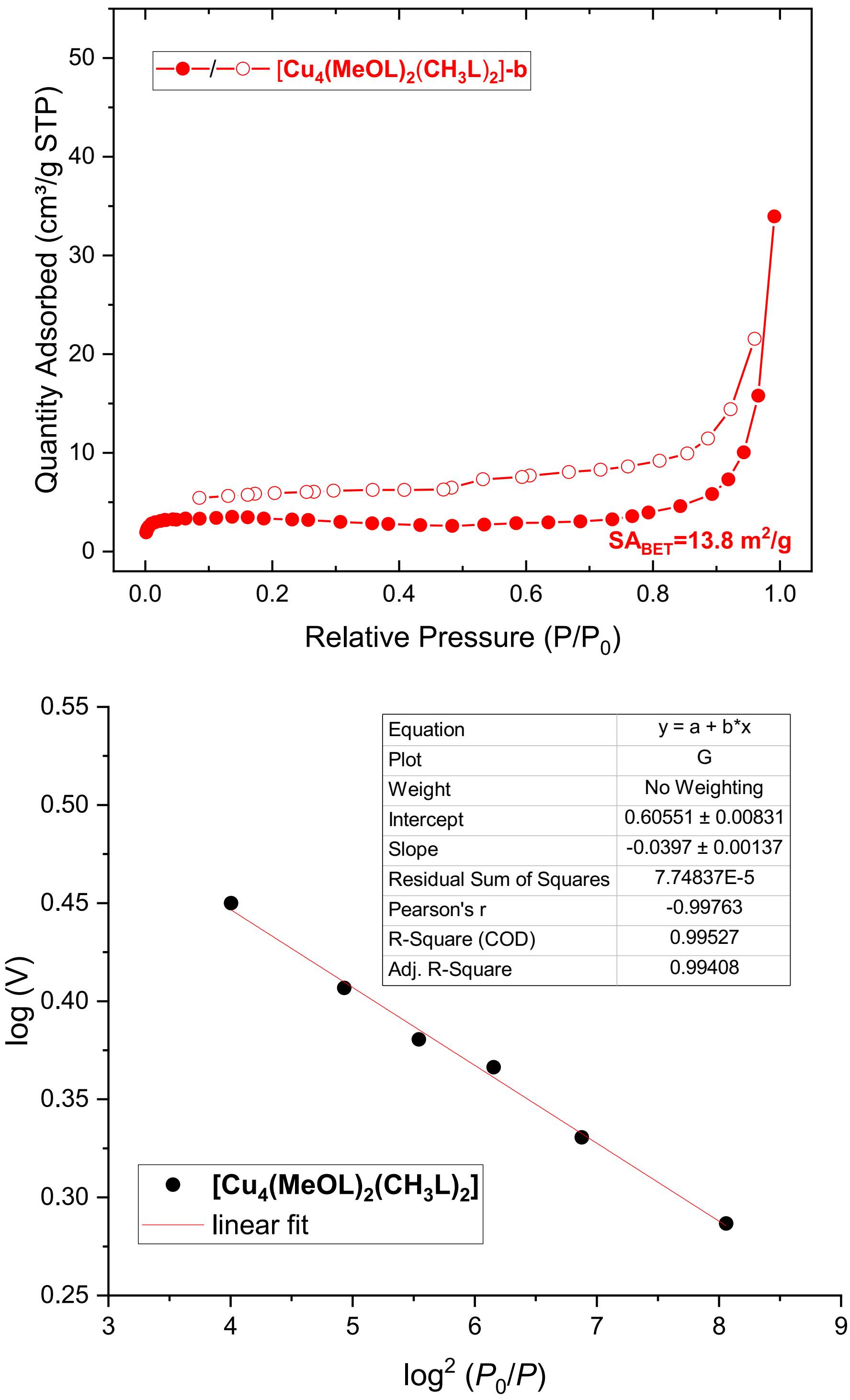

Figure S56. (top) N_2 isotherm at 77 K for **[$Cu_4(MeOL)_2(CH_3L)_2$]** and its calculated BET surface area. (bottom) Linear fit for Dubinin-Radishkevich approximation for the calculation of microporosity.

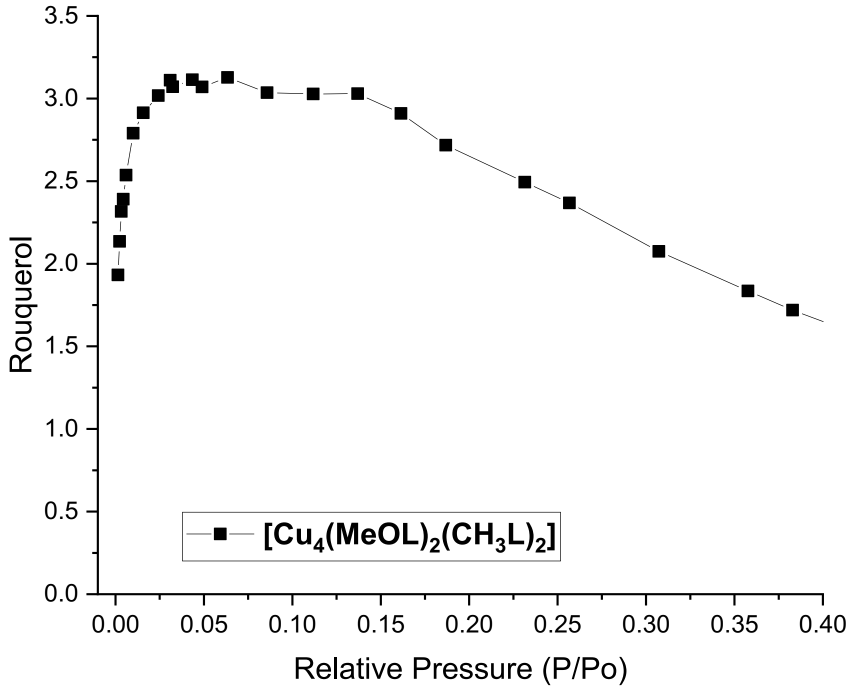

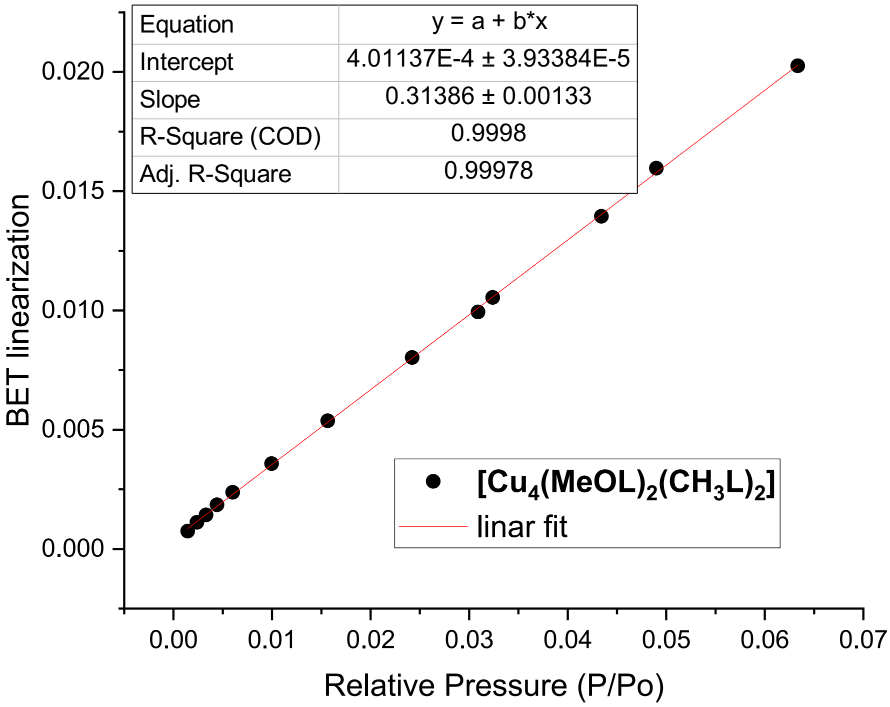

Figure S57. (top) Rouquerol graph calculated from isotherm. (bottom) Linear fit used for BET surface area calculation.

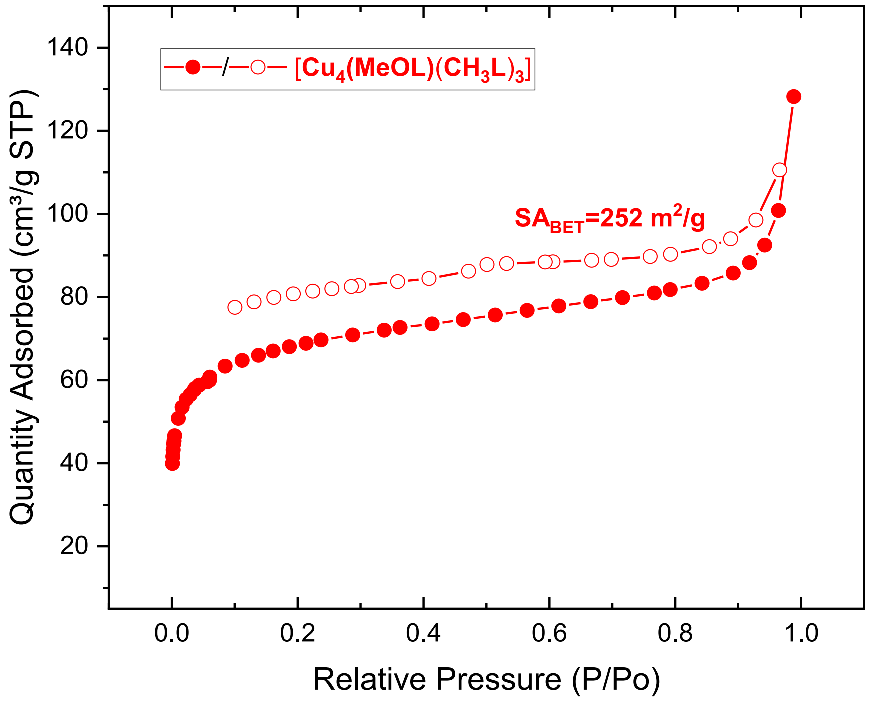

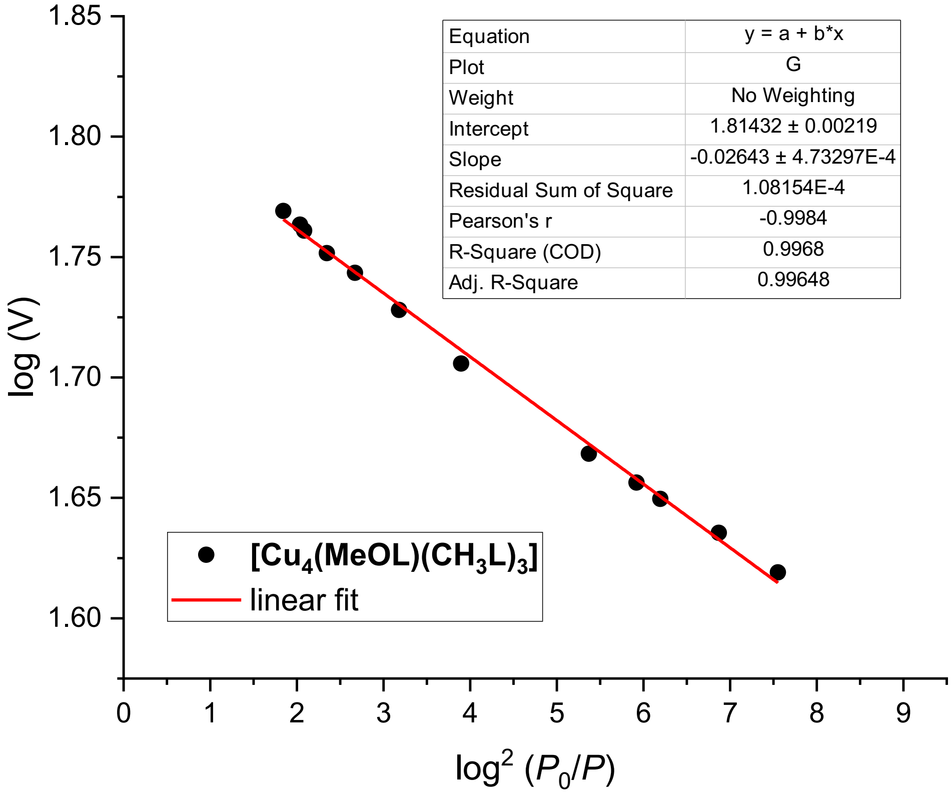

Figure S58. (top) N_2 isotherm at 77 K for **[Cu_4(MeOL)(CH_3L)$_3$]** and its calculated BET surface area. (bottom) Linear fit for Dubinin-Radishkevich approximation for the calculation of microporosity.

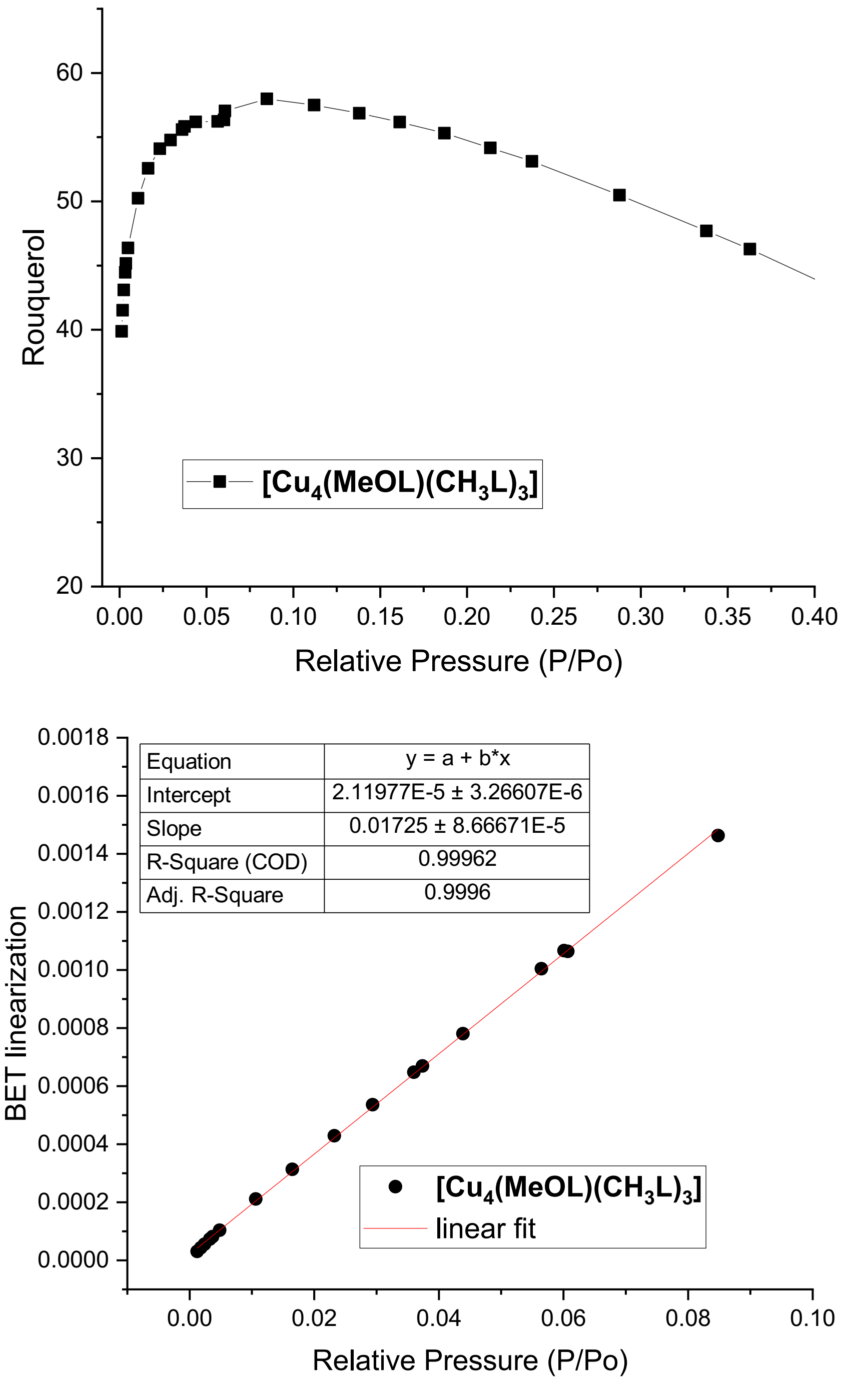

Figure S59. (top) Rouquerol graph calculated from isotherm. (bottom) Linear fit used for BET surface area calculation.

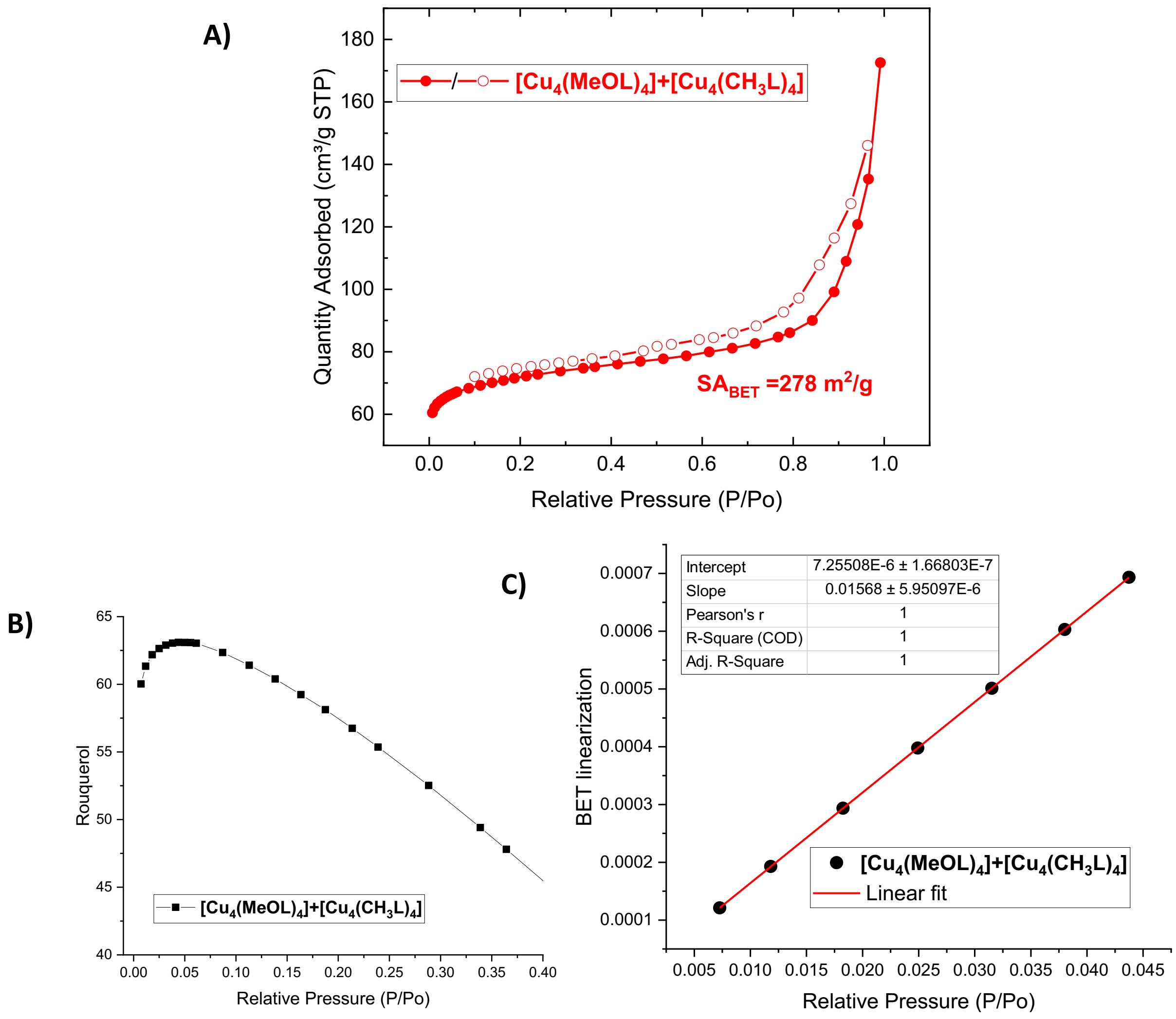

Figure S60. A) N_2 isotherm at 77 K for **$[Cu_4(MeOL)_4]$ + $[Cu_4(CH_3L)_4]$** and its calculated BET surface area. B) Rouquerol graph calculated from isotherm. C) BET linear fit used for surface area calculation.

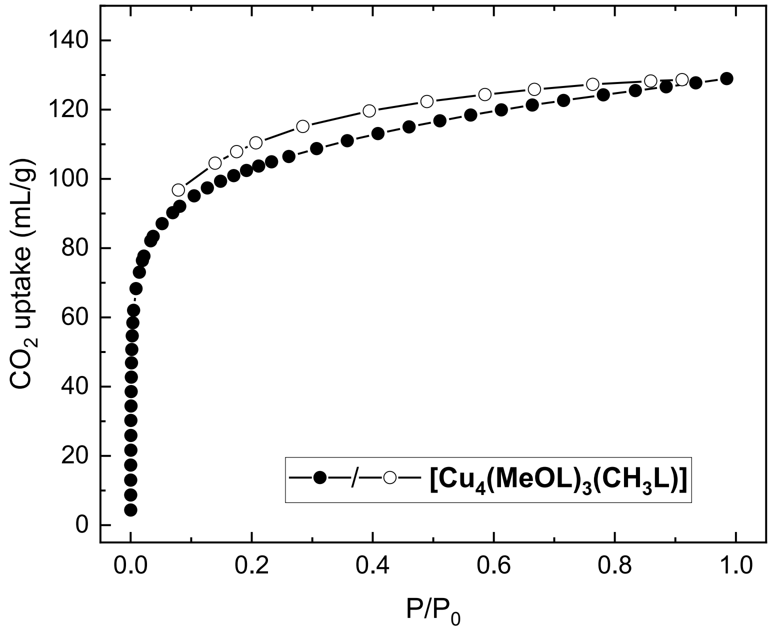

Figure S61. CO_2 isotherm at 195 K for **[$Cu_4(MeOL)_3(CH_3L)$]**.

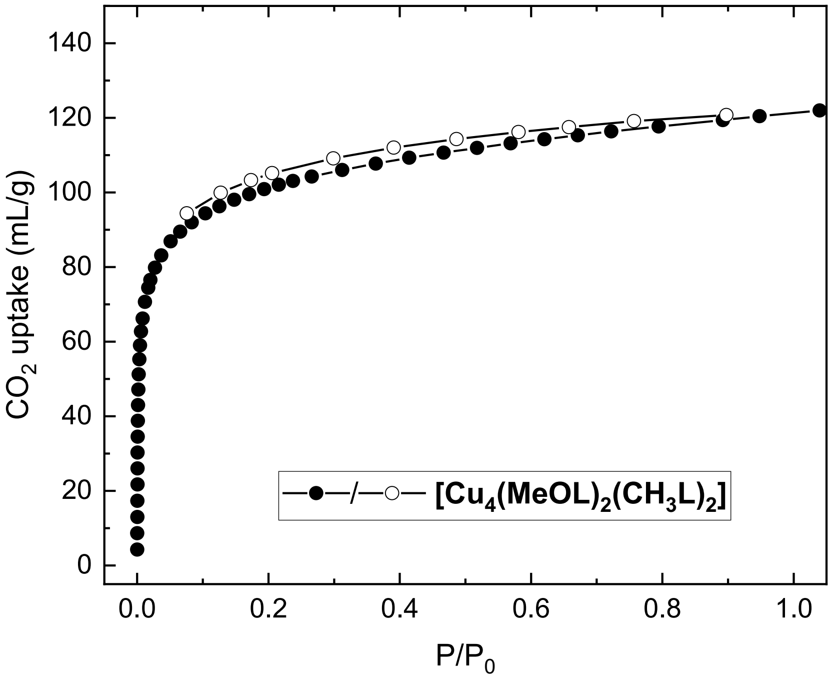

Figure S62. CO_2 isotherm at 195 K for **[$Cu_4(MeOL)_2(CH_3L)_2$]**.

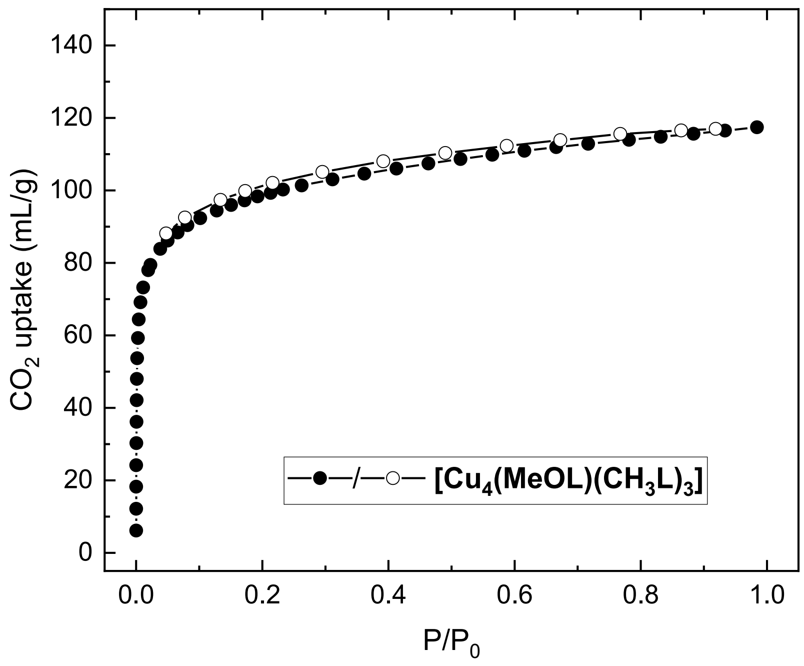

Figure S63. CO_2 isotherm at 195 K for **$[Cu_4(MeOL)(CH_3L)_3]$**.

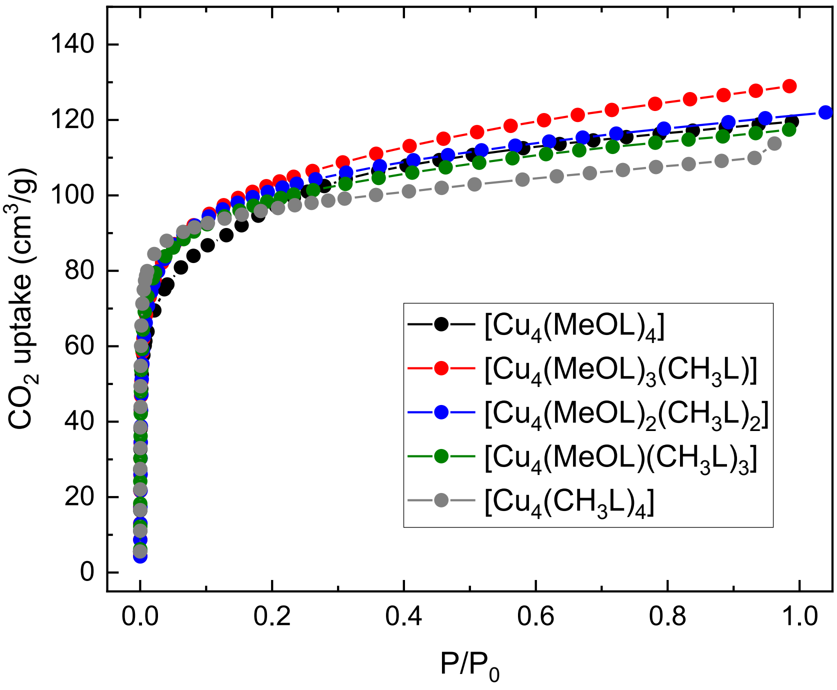

Figure S64. Compilation of CO_2 isotherms for the family $[Cu_4(MeOL)_{4-x}(CH_3L)_x]$ (x=0, 1, 2, 3, 4). Desorption branches have been omitted for clarity.

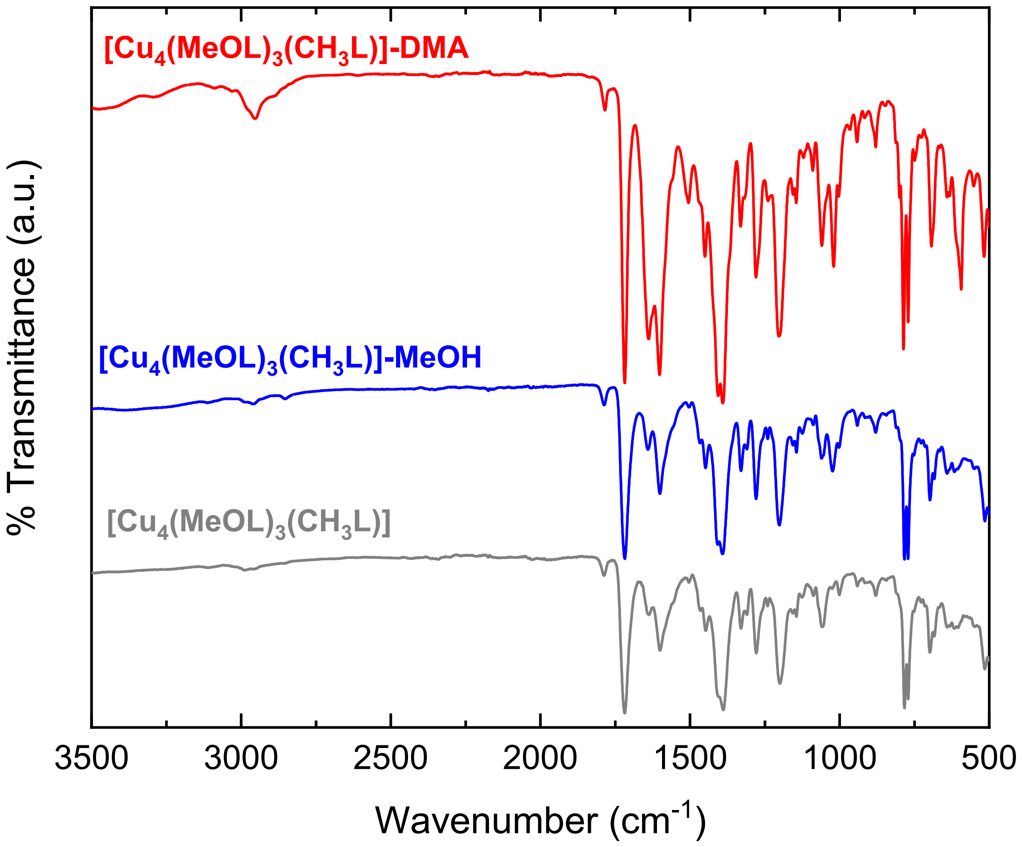

Figure S65. Infra-red spectra of bulk **[$Cu_4(MeOL)_3(CH_3L)$]-DMA** (red), after MeOH exchange **[$Cu_4(MeOL)_3(CH_3L)$]-MeOH** (blue), and after gas sorption analysis in **[$Cu_4(MeOL)_3(CH_3L)$]** (grey).

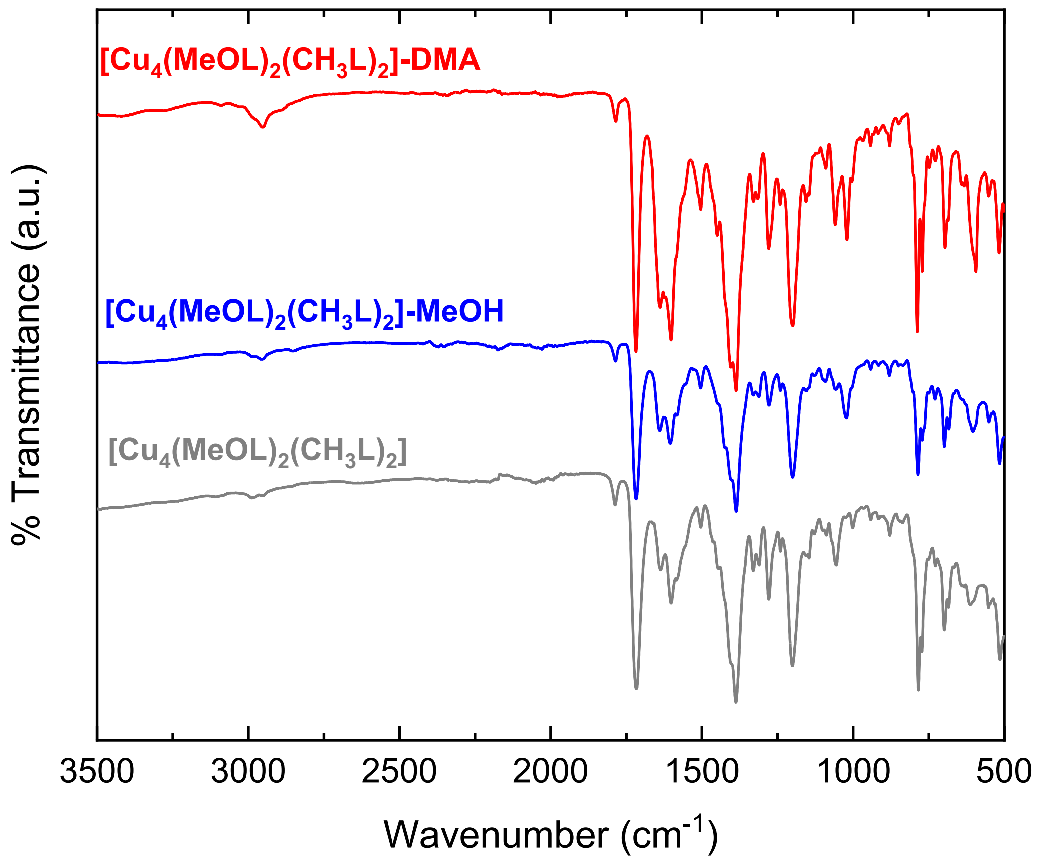

Figure S66. Infra-red spectra of bulk **[$Cu_4(MeOL)_2(CH_3L)_2$]-DMA** (red), after MeOH exchange **[$Cu_4(MeOL)_2(CH_3L)_2$]-MeOH** (blue), and after gas sorption analysis in **[$Cu_4(MeOL)_2(CH_3L)_2$]** (grey).

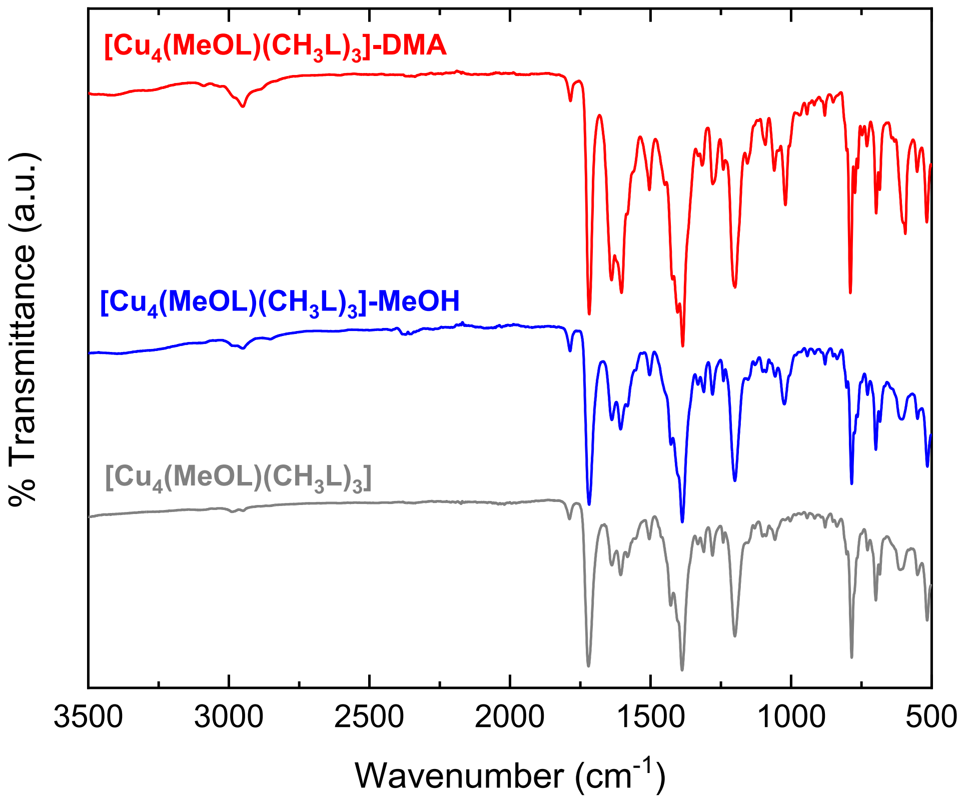

Figure S67. Infra-red spectra of bulk **[Cu_4(MeOL)(CH_3L)$_3$]-DMA** (red), after MeOH exchange **[Cu_4(MeOL)(CH_3L)$_3$]-MeOH** (blue), and after gas sorption analysis in **[Cu_4(MeOL)(CH_3L)$_3$]** (grey).

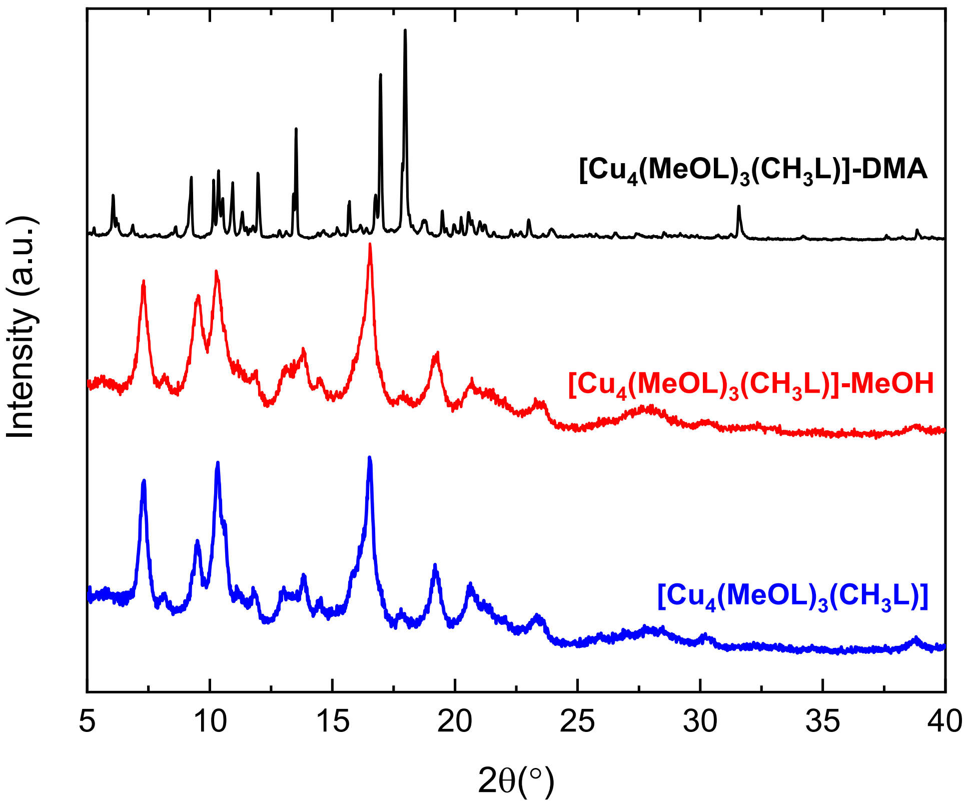

Figure S68. Powder X-ray diffractograms of bulk **[$Cu_4(MeOL)_3(CH_3L)$]-DMA** (black), after MeOH exchange **[$Cu_4(MeOL)_3(CH_3L)$]-MeOH** (red), and after gas sorption analysis in **[$Cu_4(MeOL)_3(CH_3L)$]** (blue).

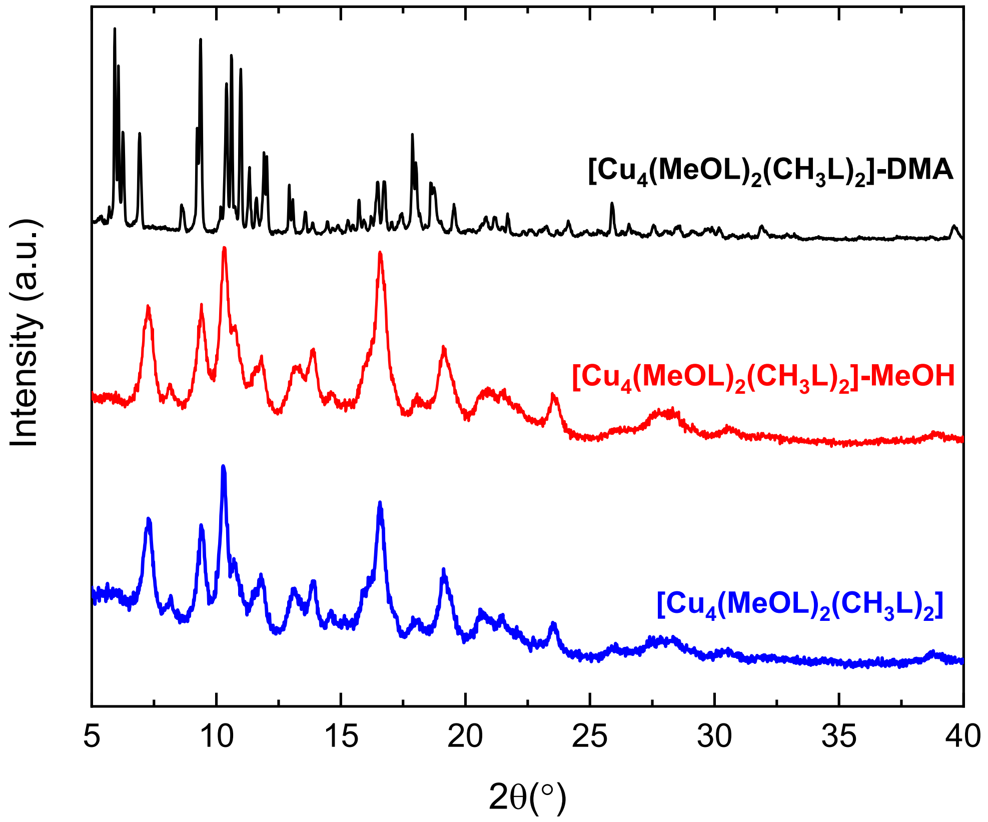

Figure S69. Powder X-ray diffractograms of bulk **[Cu$_4$(MeOL)$_2$(CH$_3$L)$_2$]-DMA** (black), after MeOH exchange **[Cu$_4$(MeOL)$_2$(CH$_3$L)$_2$]-MeOH** (red), and after gas sorption analysis in **[Cu$_4$(MeOL)$_2$(CH$_3$L)$_2$]** (blue).

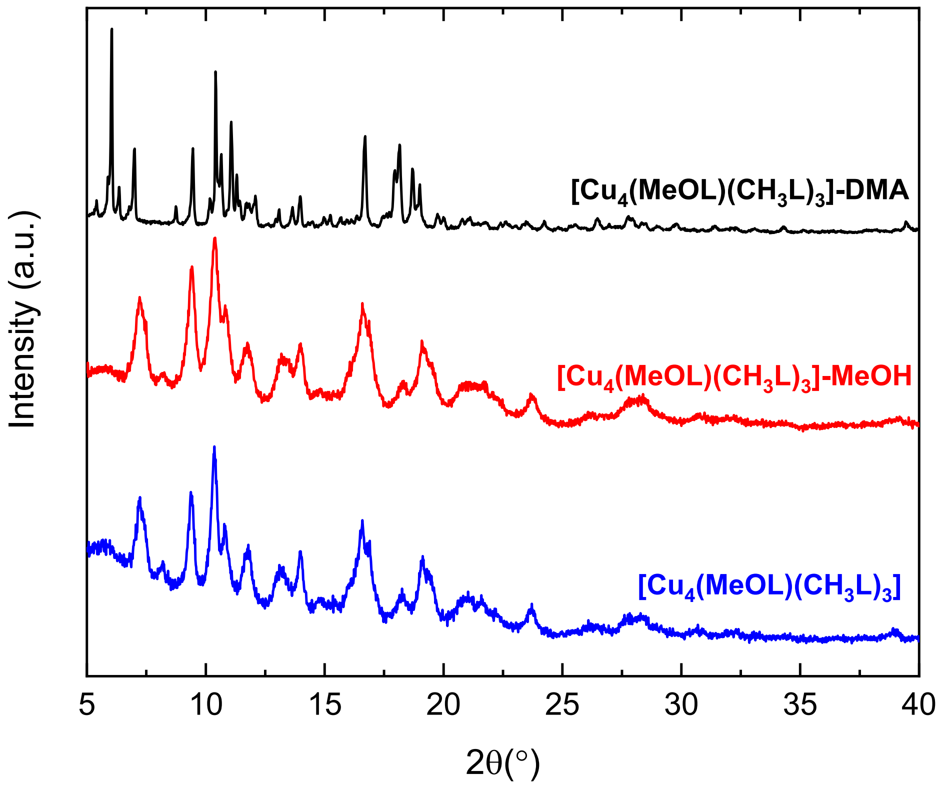

Figure S70. Powder X-ray diffractograms of bulk **[Cu$_4$(MeOL)(CH$_3$L)$_3$]-DMA** (black), after MeOH exchange **[Cu$_4$(MeOL)(CH$_3$L)$_3$]-MeOH** (red), and after gas sorption analysis in **[Cu$_4$(MeOL)(CH$_3$L)$_3$]** (blue).

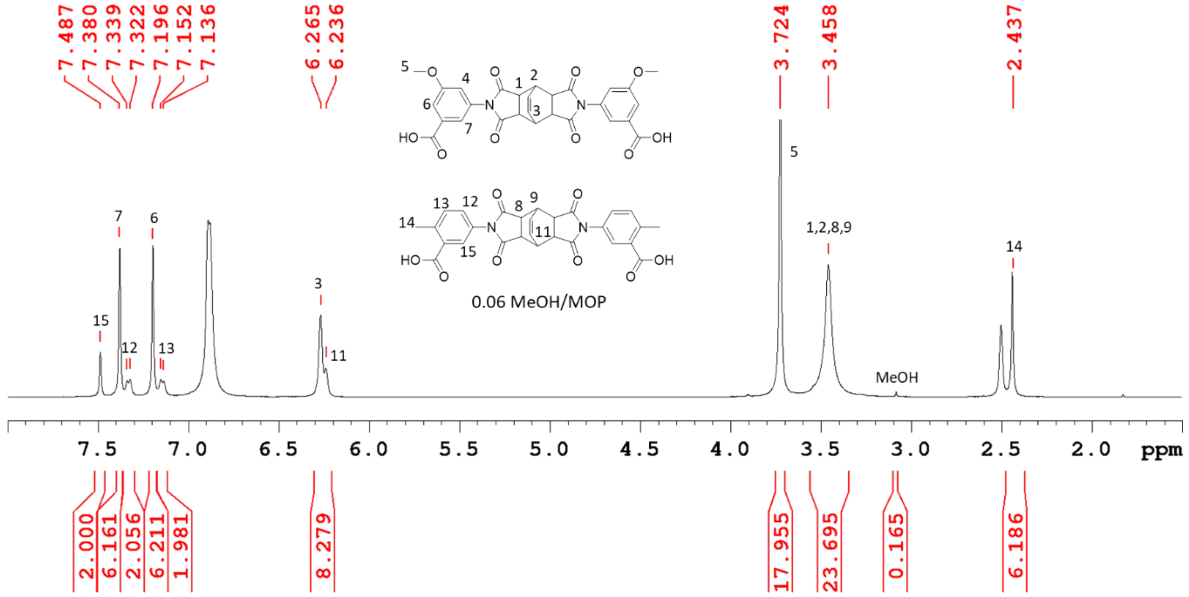

Figure S71. ^{1}H-NMR spectrum of the digestion in DMSO/DCl of **[$Cu_4(MeOL)_3(CH_3L)$]** after gas sorption measurements.

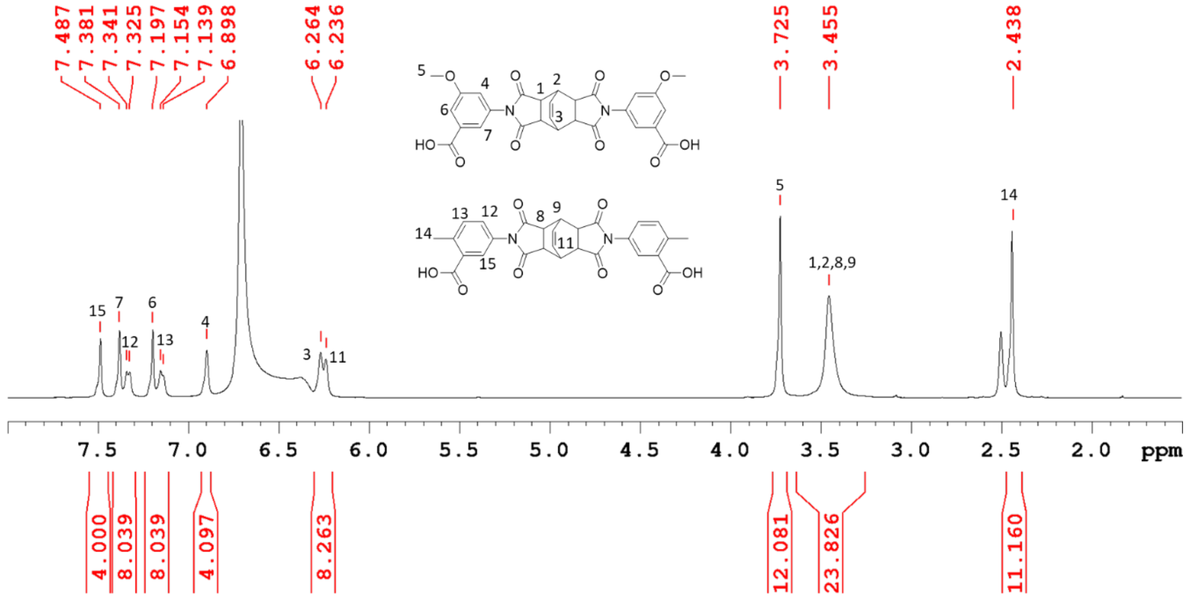

Figure S72. ^{1}H-NMR spectrum of the digestion in DMSO/DCl of **[$Cu_4(MeOL)_2(CH_3L)_2$]** after gas sorption measurements

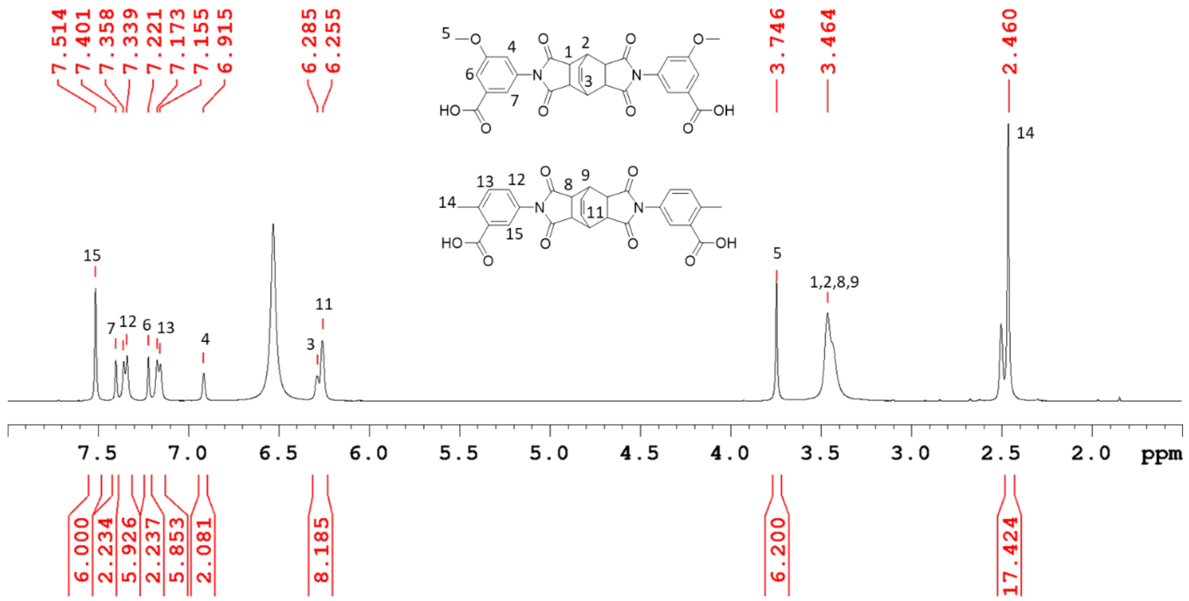

Figure S73. ^{1}H-NMR spectrum of the digestion in DMSO/DCl of **[Cu$_4$(MeOL)(CH$_3$L)$_3$]** after gas sorption measurements

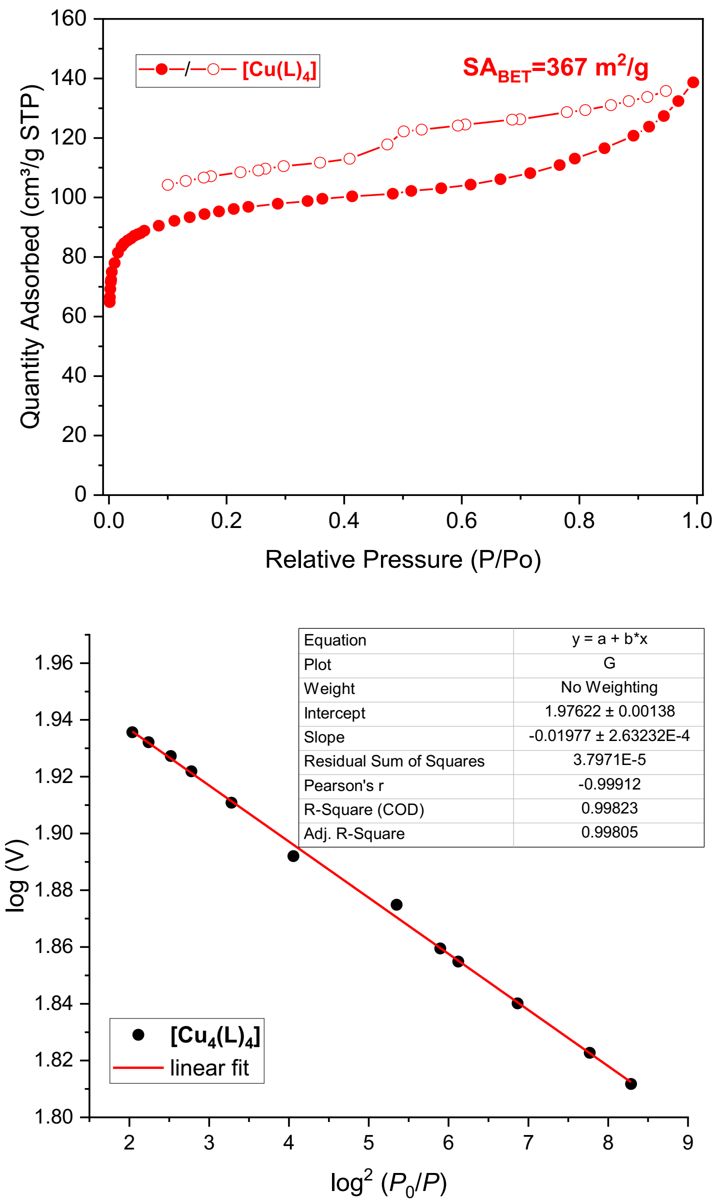

Figure S74. (top) N_2 isotherm at 77 K for **[Cu₄(L)₄]** and its calculated BET surface area. (bottom) Linear fit for Dubinin-Radishkevich approximation for the calculation of microporosity.

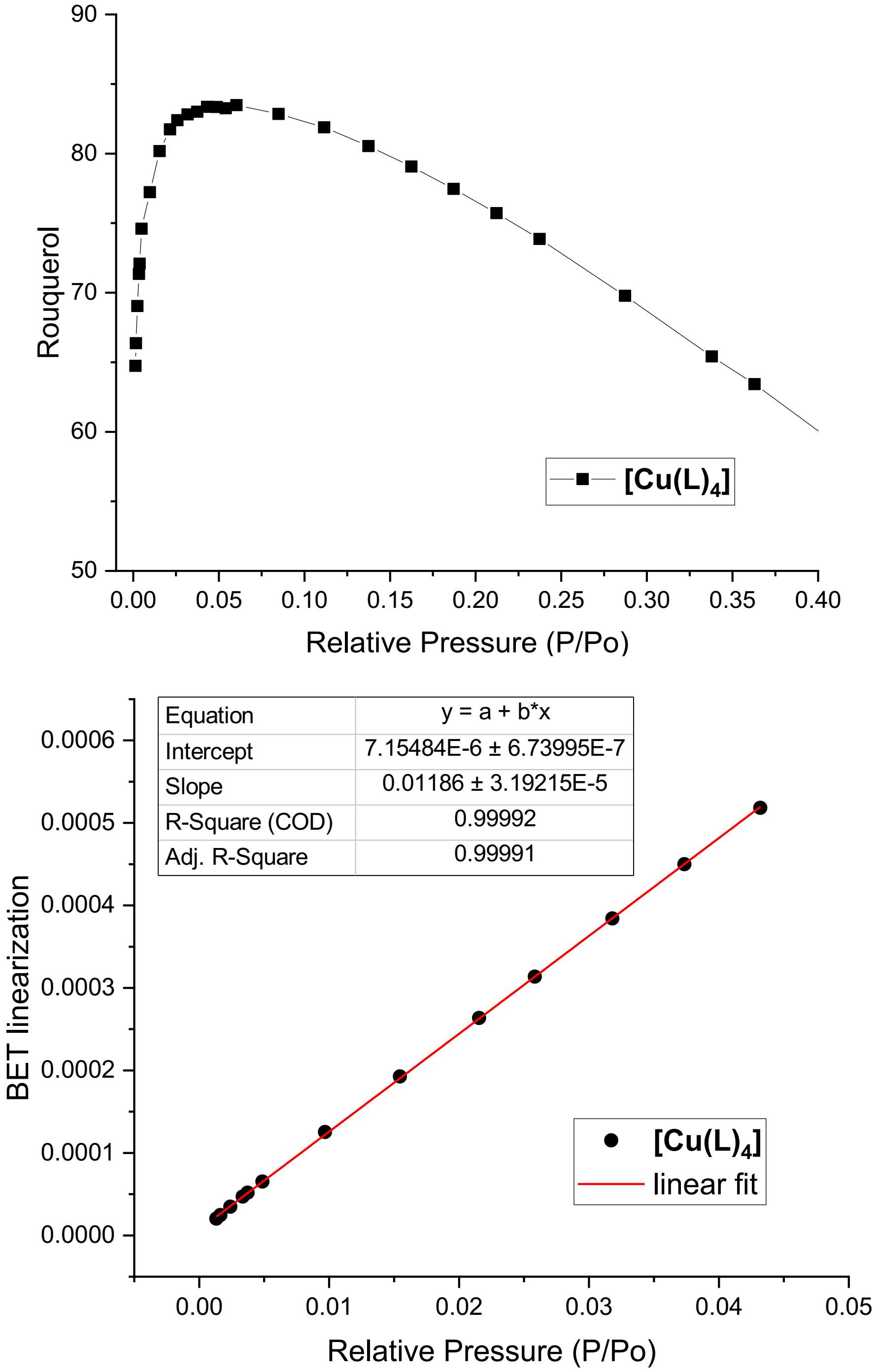

Figure S75. (top) Rouquerol graph calculated from isotherm. (bottom) Linear fit used for BET surface area calculation.

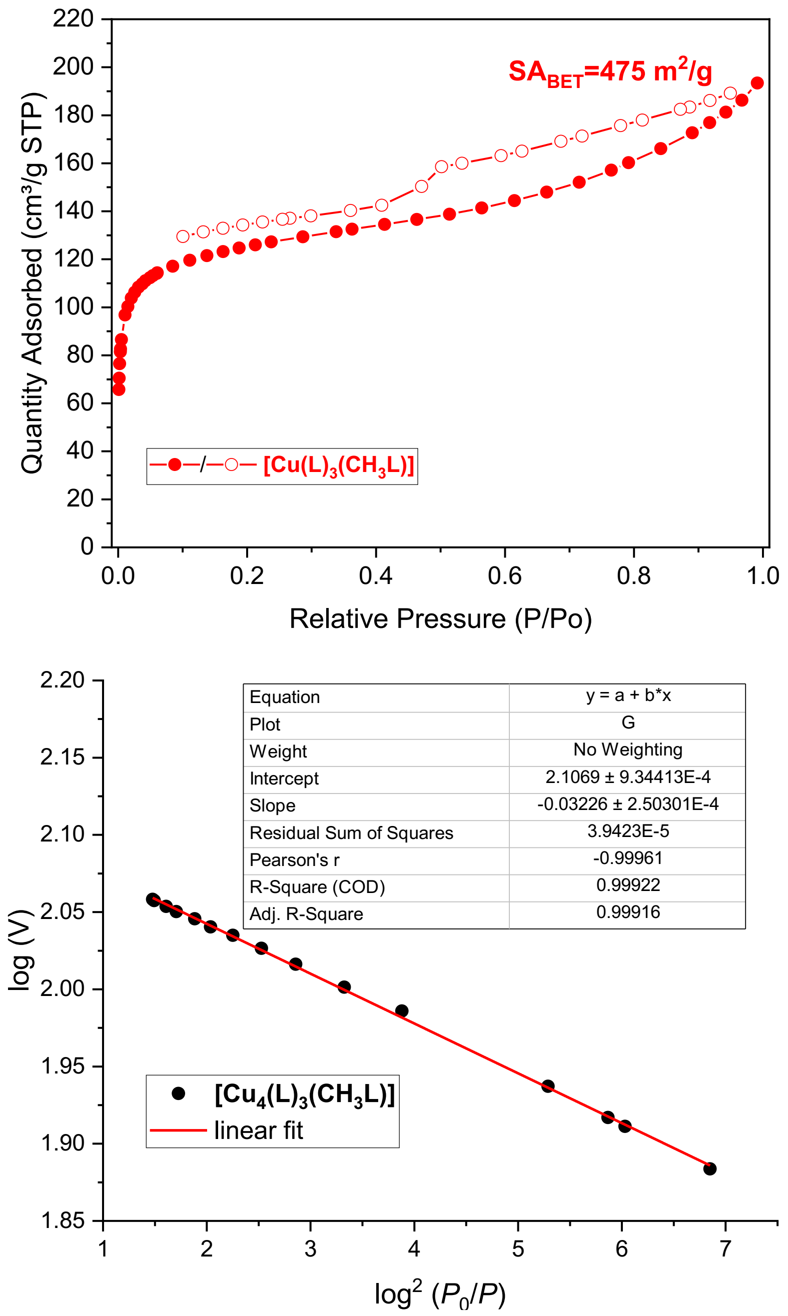

Figure S76. (top) N_2 isotherm at 77 K for **[Cu$_4$(L)$_3$(CH$_3$L)]** and its calculated BET surface area. (bottom) Linear fit for Dubinin-Radishkevich approximation for the calculation of microporosity.

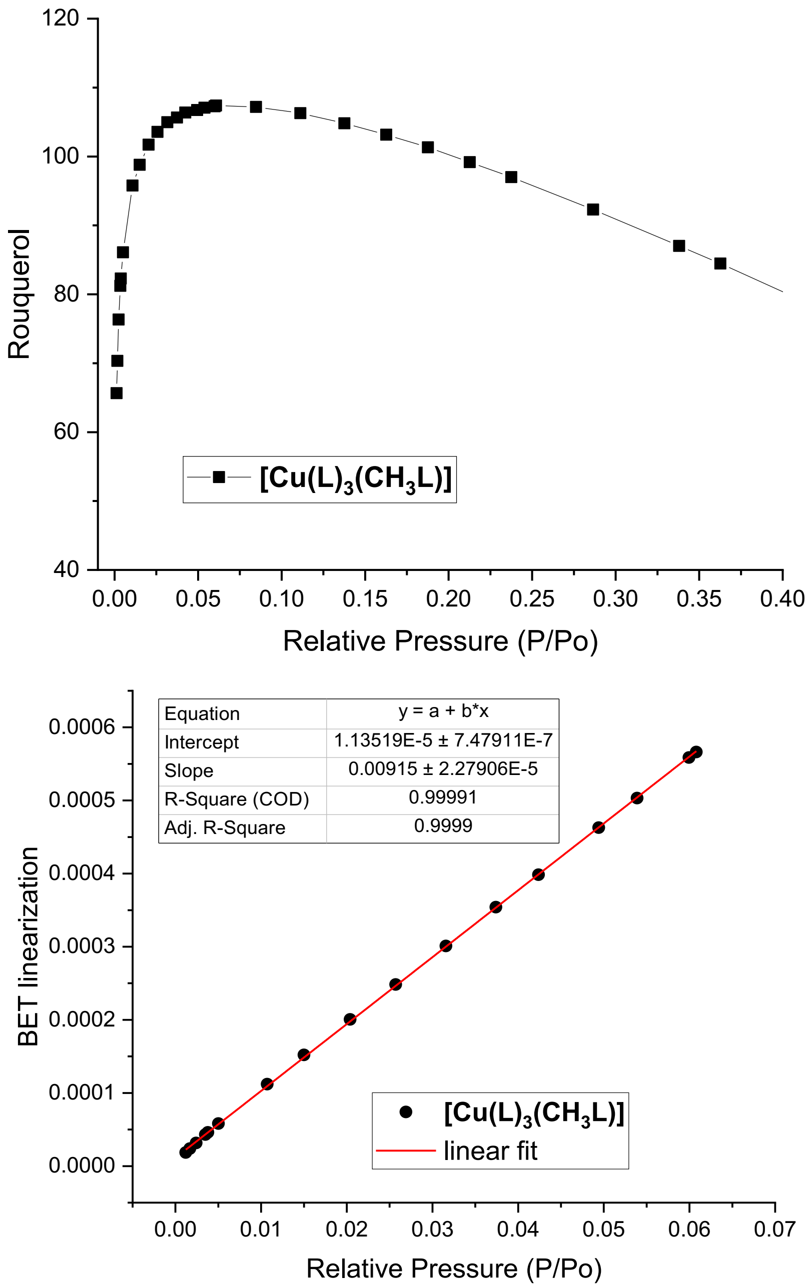

Figure S77. (top) Rouquerol graph calculated from isotherm. (bottom) BET linear fit used for surface area calculation.

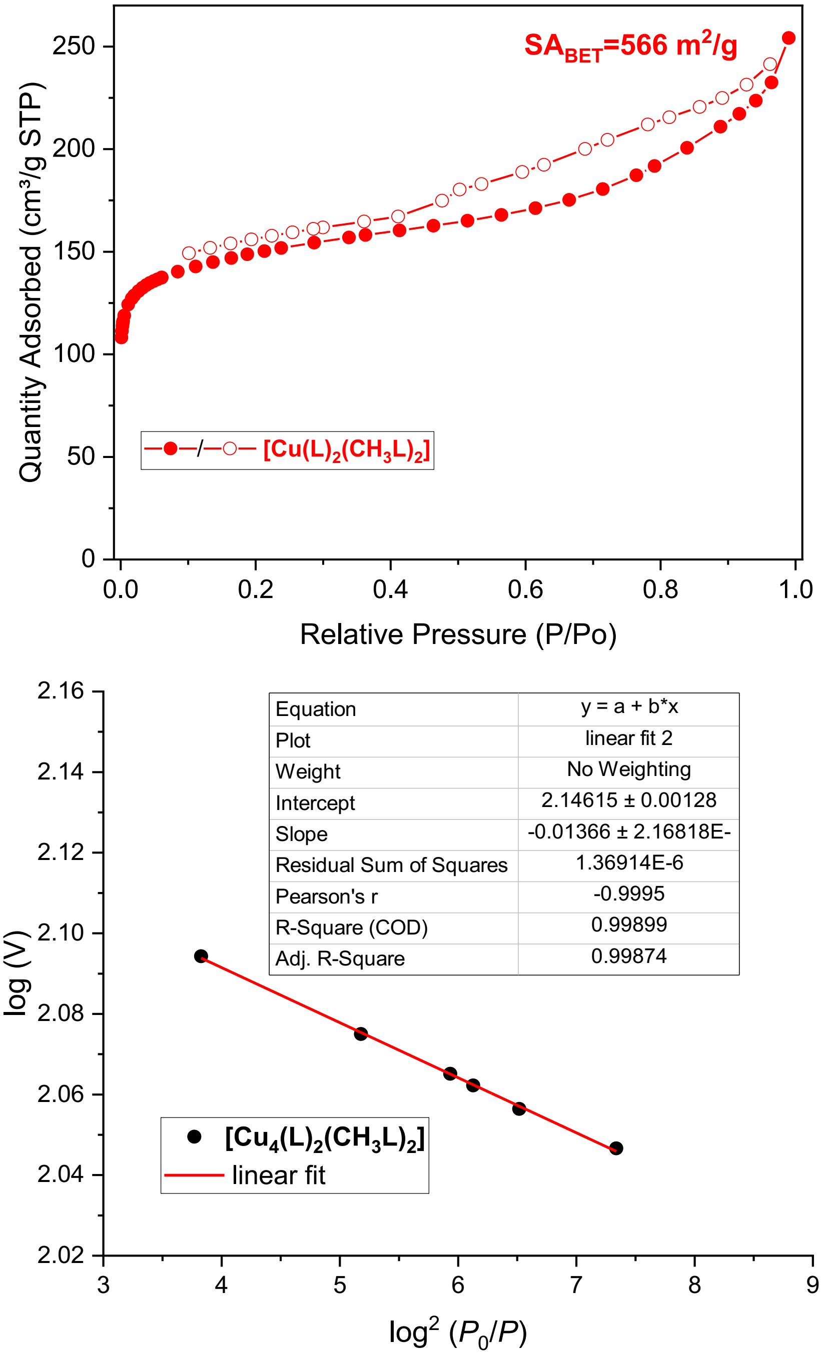

Figure S78. (top) N_2 isotherm at 77 K for **[Cu$_4$(L)$_2$(CH$_3$L)$_2$]** and its calculated BET surface area. (bottom) Linear fit for Dubinin-Radishkevich approximation for the calculation of microporosity.

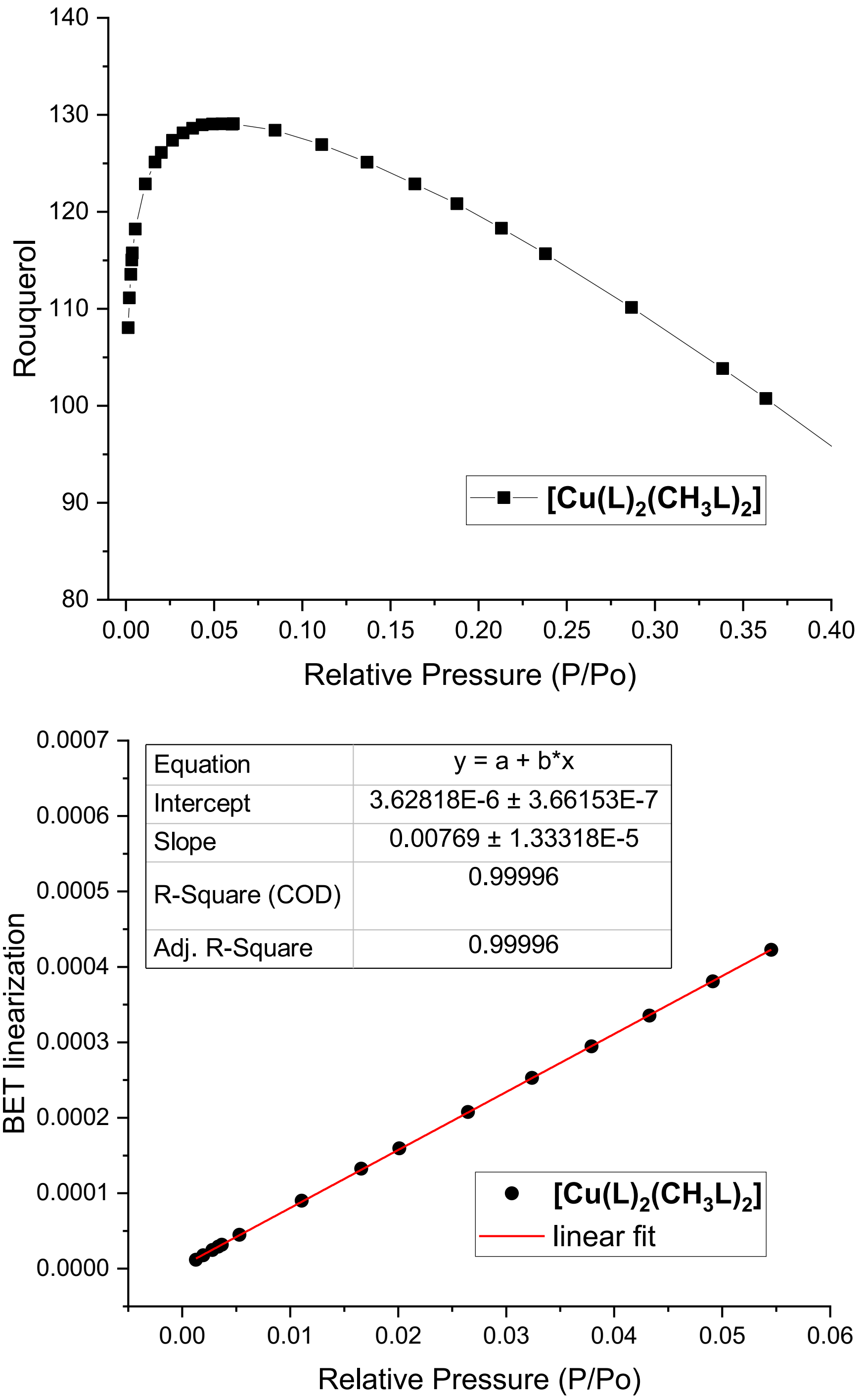

Figure S79. (top) Rouquerol graph calculated from isotherm. (bottom) BET linear fit used for surface area calculation.

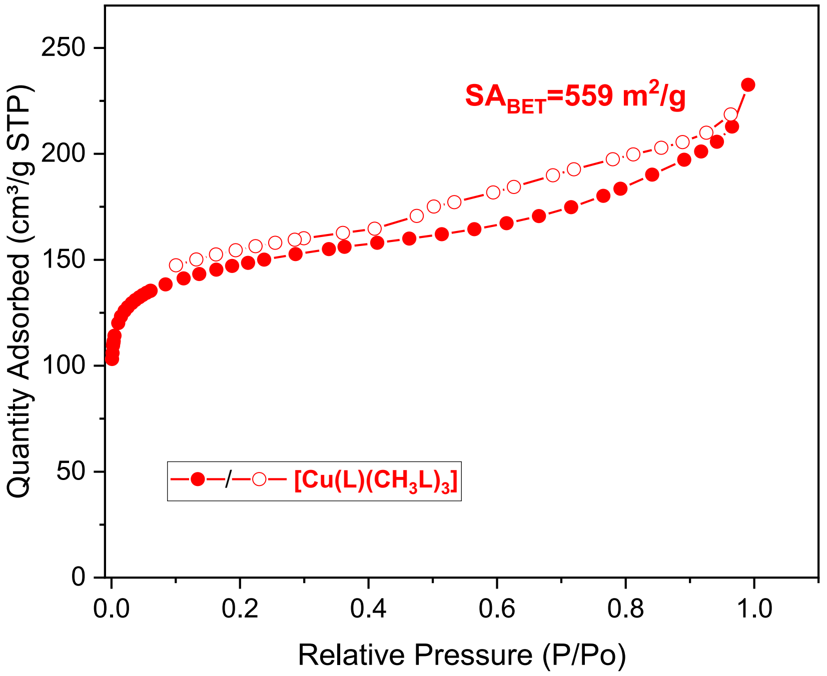

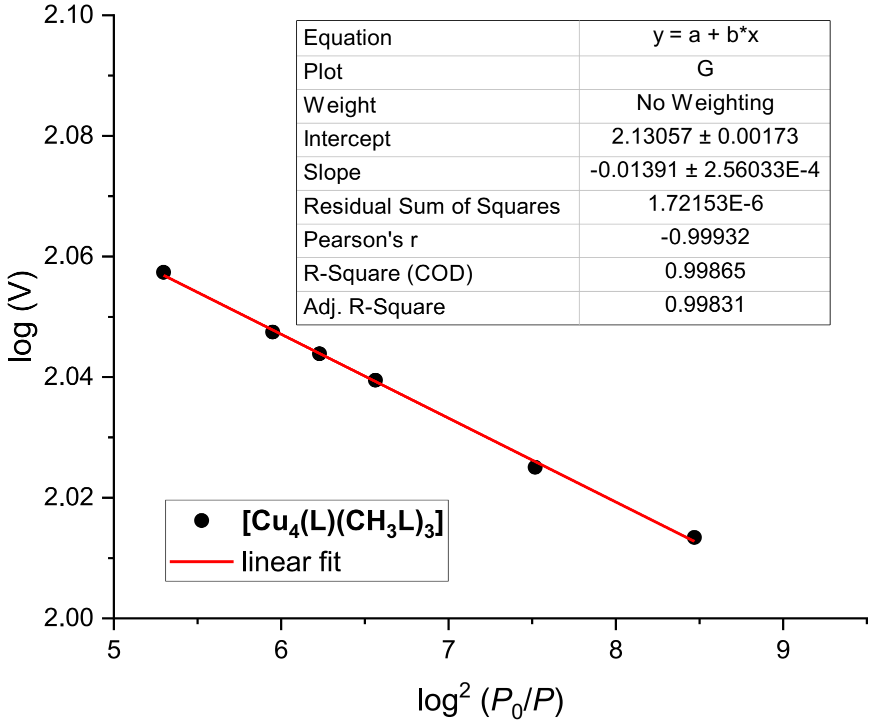

Figure S80. (top) N_2 isotherm at 77 K for **[Cu$_4$(L)(CH$_3$L)$_3$]** and its calculated BET surface area. (bottom) Linear fit for Dubinin-Radishkevich approximation for the calculation of microporosity.

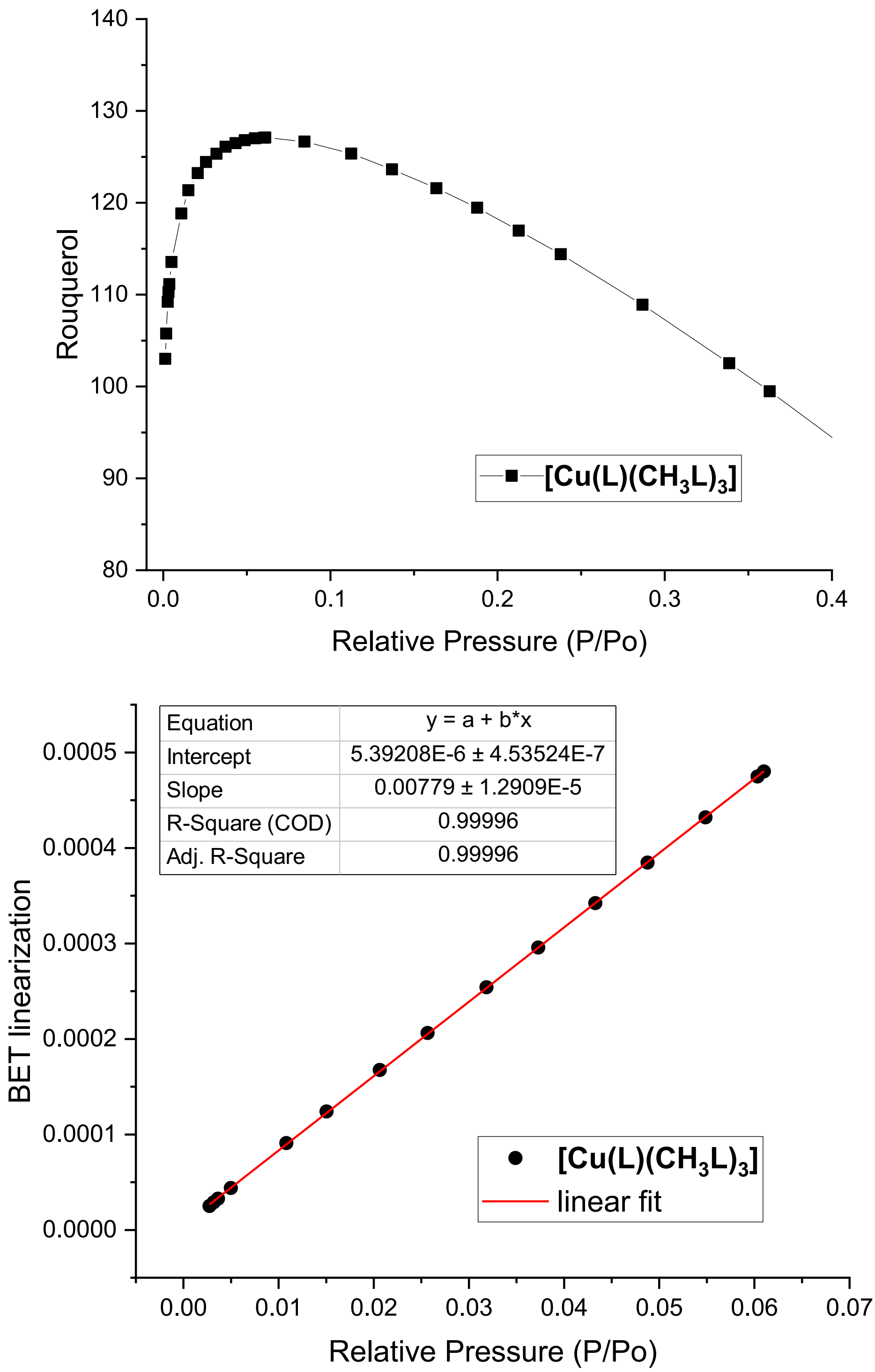

Figure S81. (top) Rouquerol graph calculated from isotherm. (bottom) BET linear fit used for surface area calculation.

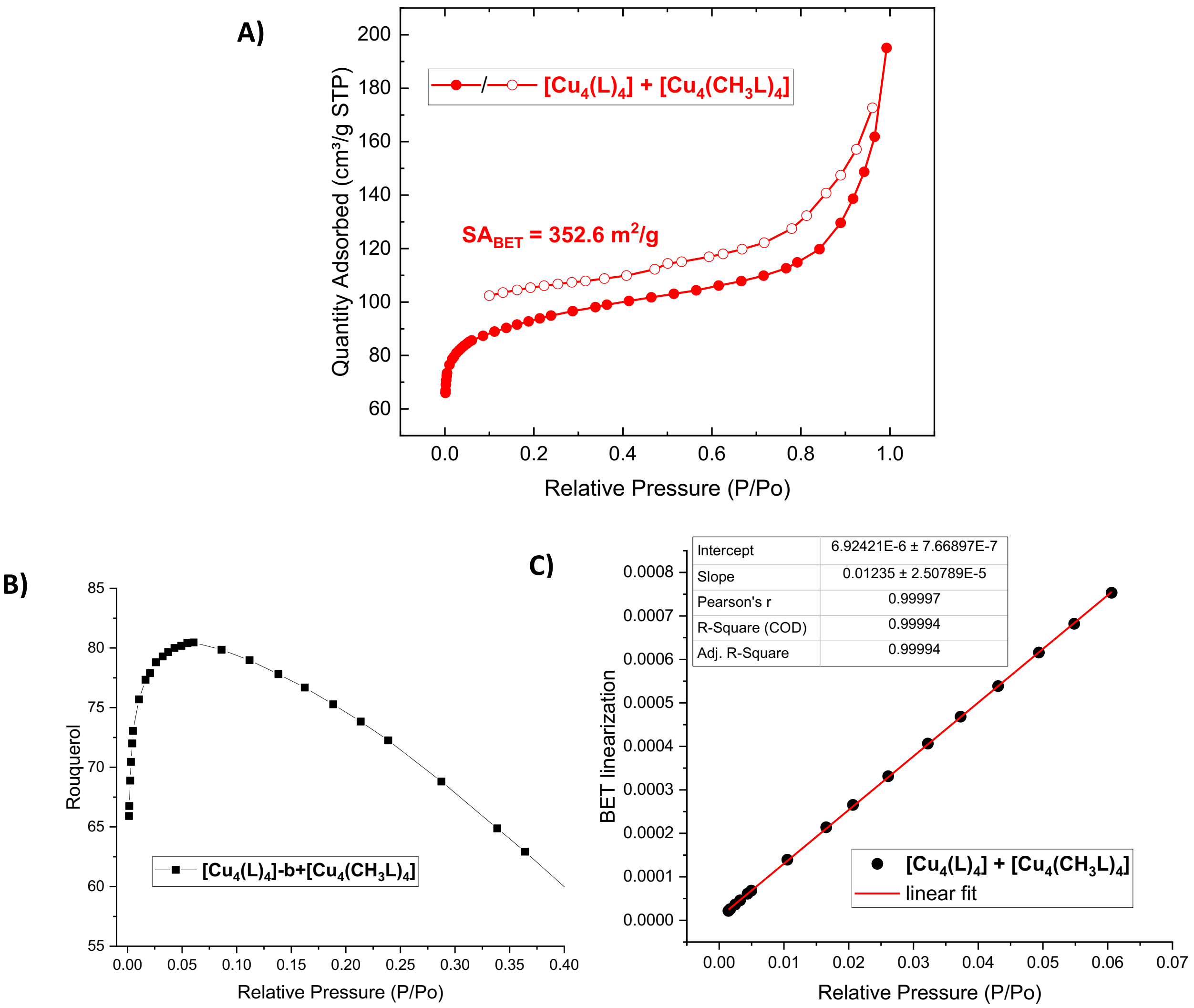

Fig. S82. A) N_2 isotherm at 77 K for **[Cu4(L)4] + [Cu4(CH3L)4]** and its calculated BET surface area. B) Rouquerol graph calculated from isotherm. C) BET linearization used for BET surface area calculation.

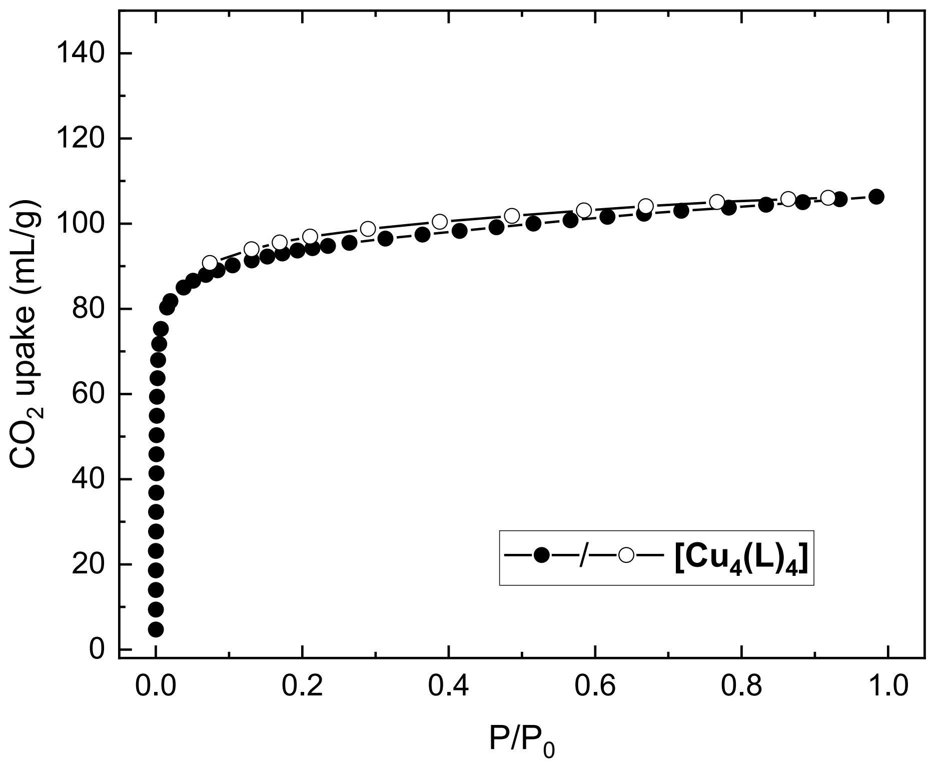

Figure S83. CO_2 isotherm at 195 K for **[Cu$_4$(L)$_4$]**.

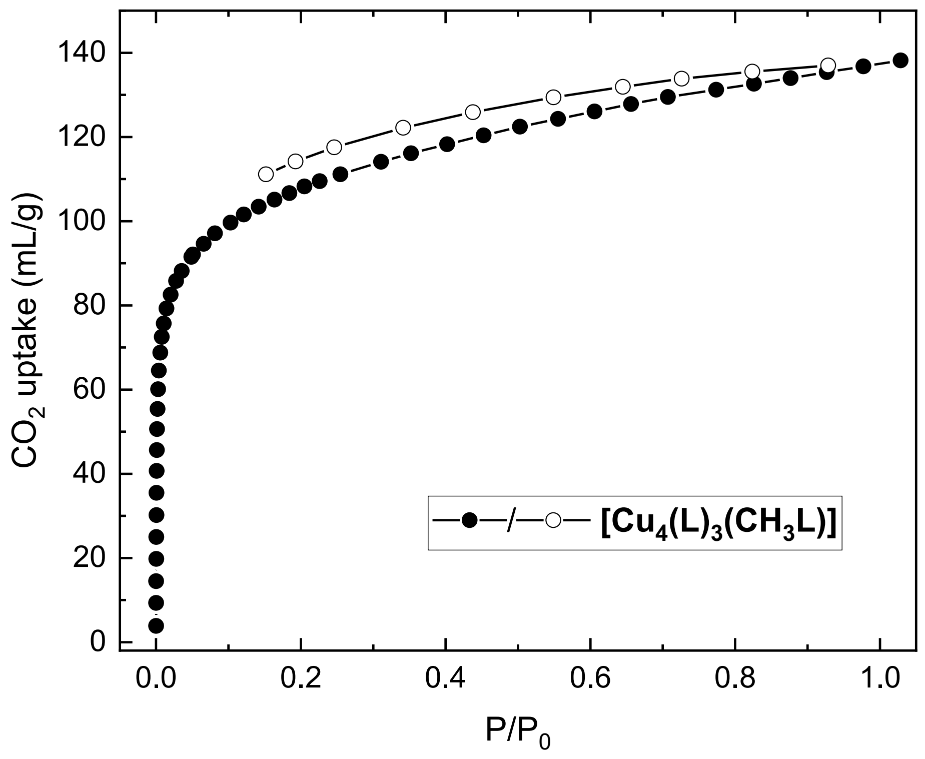

Figure S84. CO_2 isotherm at 195 K for **[Cu$_4$(L)$_3$(CH$_3$L)]**.

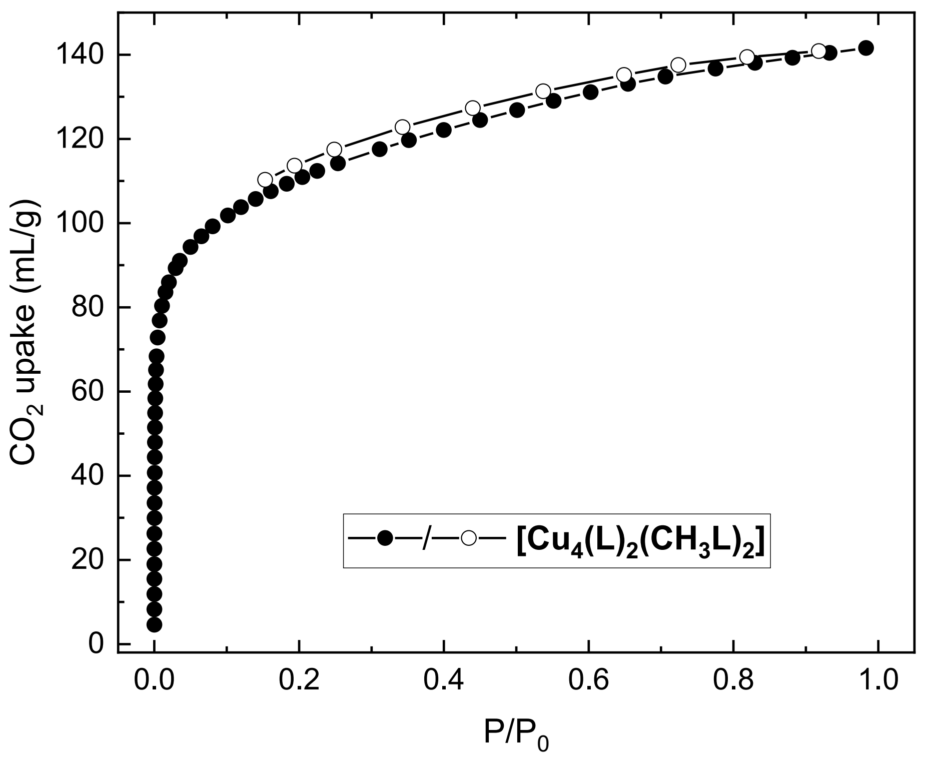

Figure S85. CO_2 isotherm at 195 K for **[Cu$_4$(L)$_2$(CH$_3$L)$_2$]**.

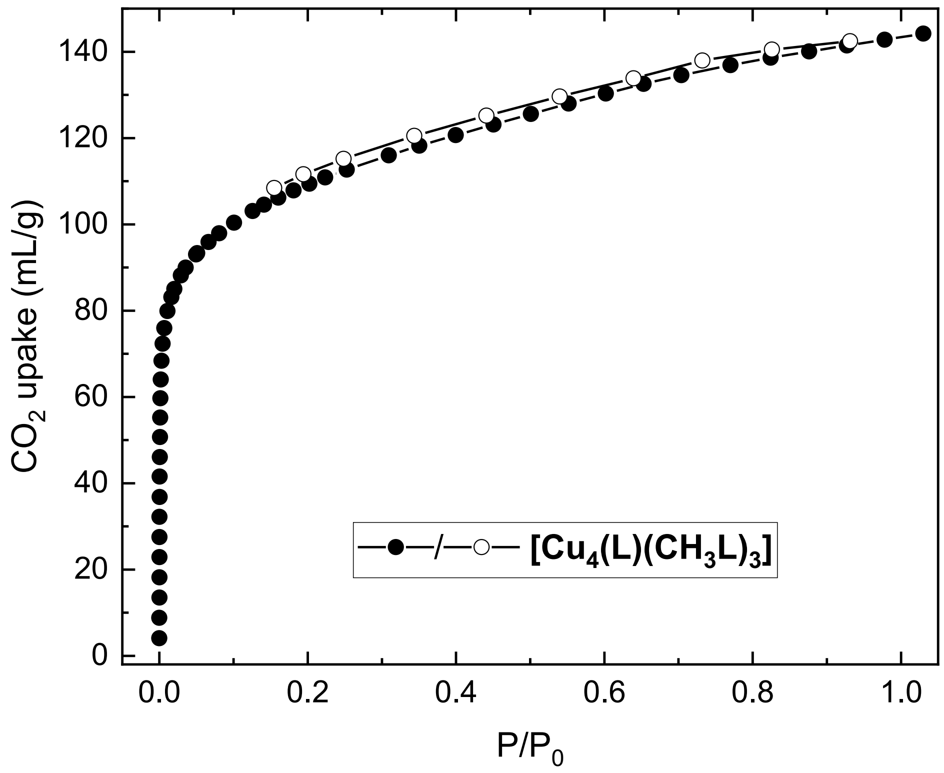

Figure S86. CO_2 isotherm at 195 K for **[Cu$_4$(L)(CH$_3$L)$_3$]**.

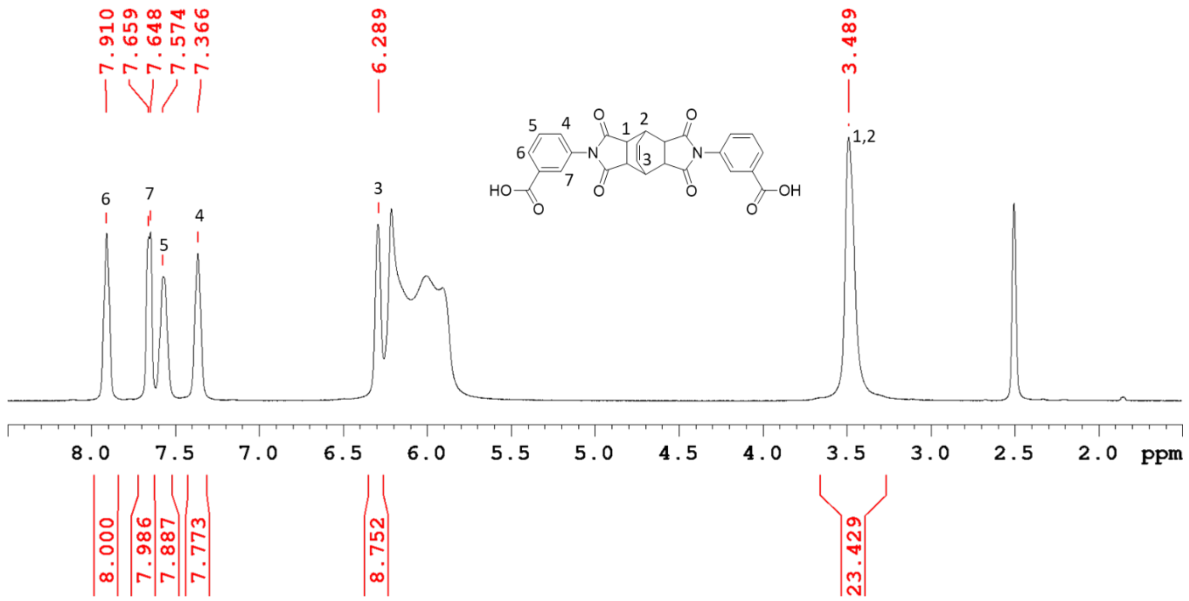

Figure S87. ^{1}H-NMR spectrum of the digestion in DMSO/DCl of **[$Cu_4(L)_4$]** after gas sorption measurements.

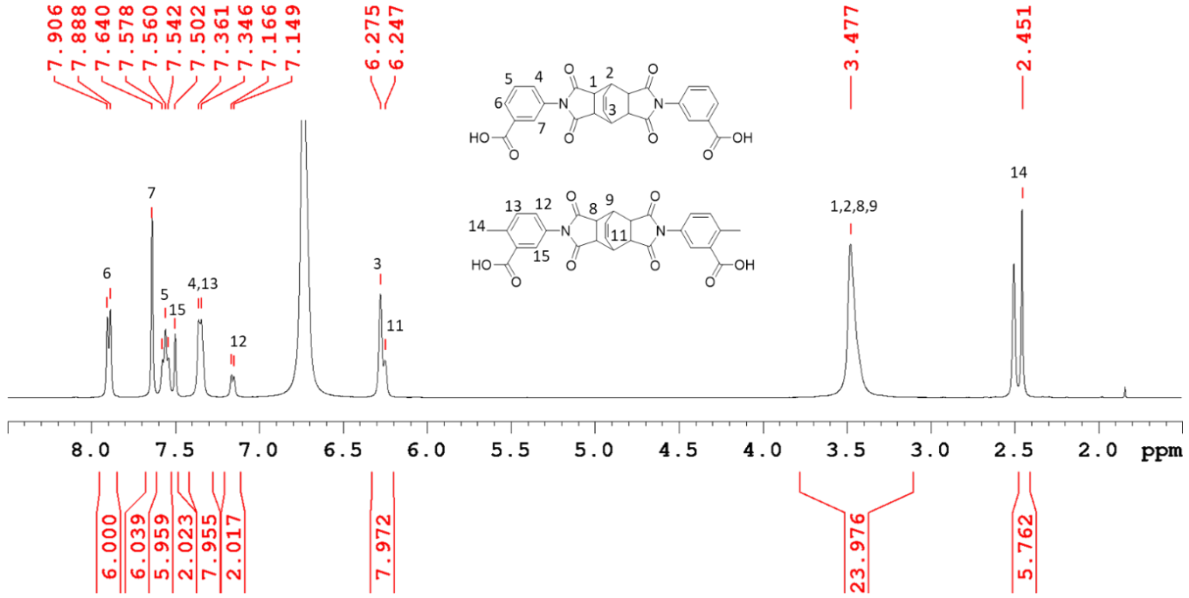

Figure S88. ^{1}H-NMR spectrum of the digestion in DMSO/DCl of **[$Cu_4(L)_3(CH_3L)$]** after gas sorption measurements.

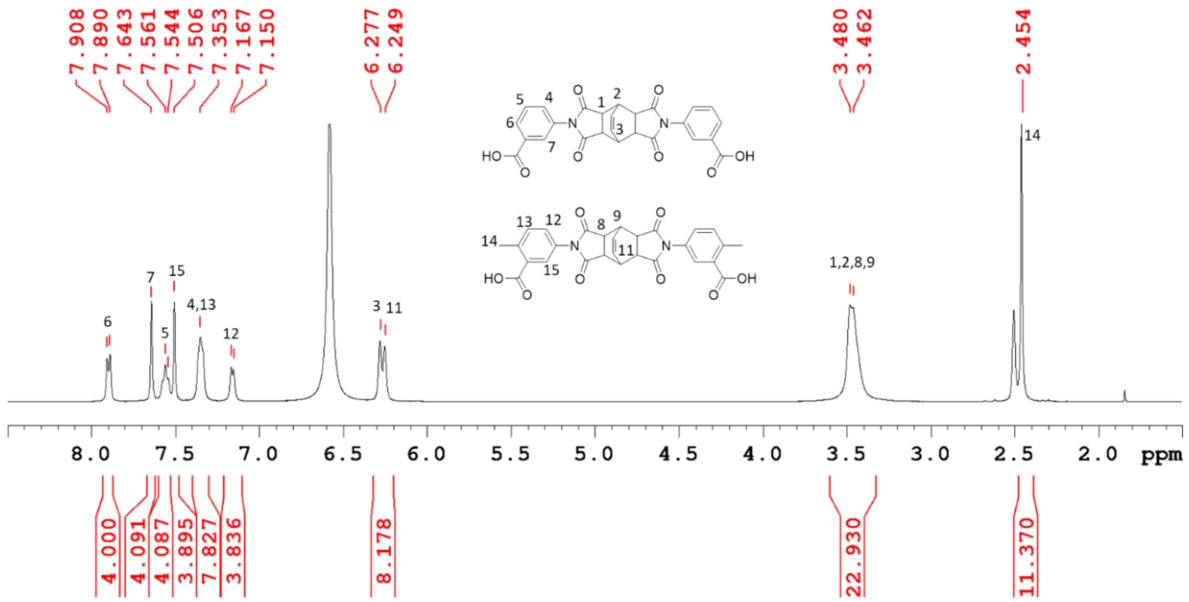

Figure S89. ^{1}H-NMR spectrum of the digestion in DMSO/DCl of **[$Cu_4(L)_2(CH_3L)_2$]** after gas sorption measurements.

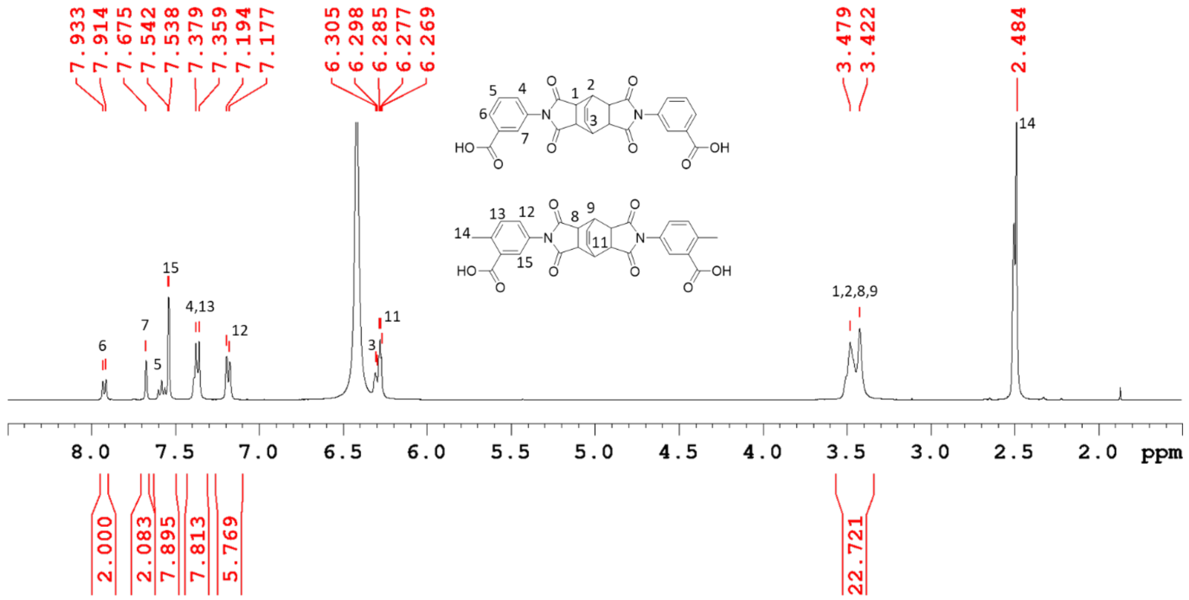

Figure S90. ^{1}H-NMR spectrum of the digestion in DMSO/DCl of **[$Cu_4(L)(CH_3L)_3$]** after gas sorption measurements.

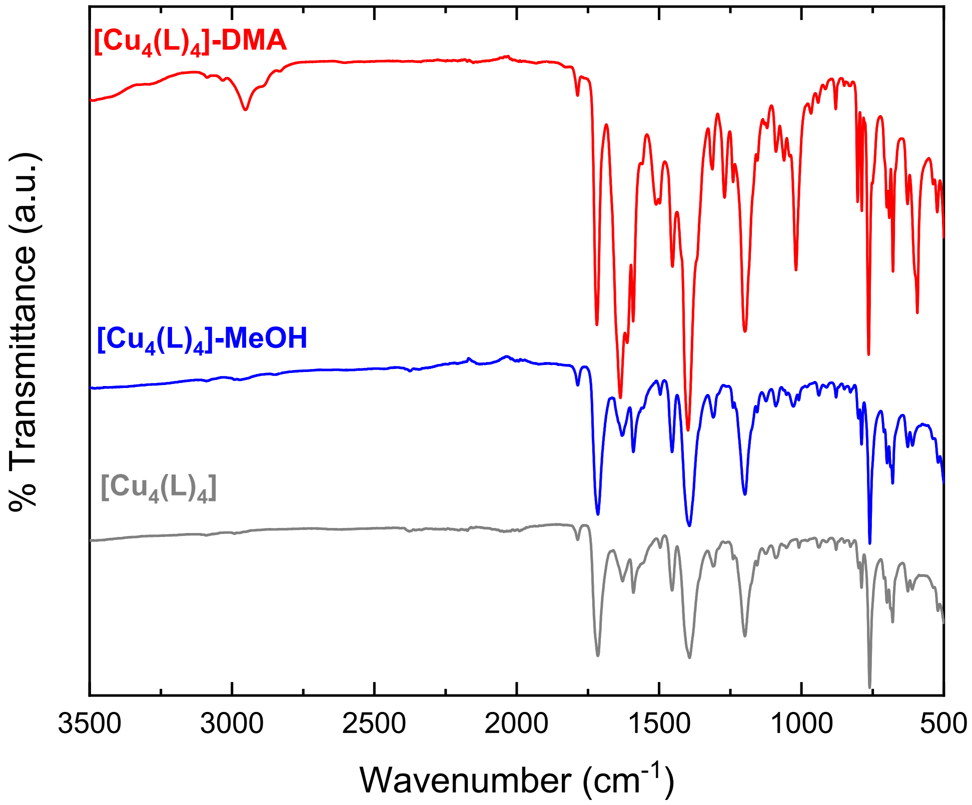

Figure S91. Infra-red spectra of bulk **[$Cu_4(L)_4$]-DMA** (red), after MeOH exchange **[$Cu_4(L)_4$]-MeOH** (blue), and after gas sorption analysis in **[$Cu_4(L)_4$]** (grey).

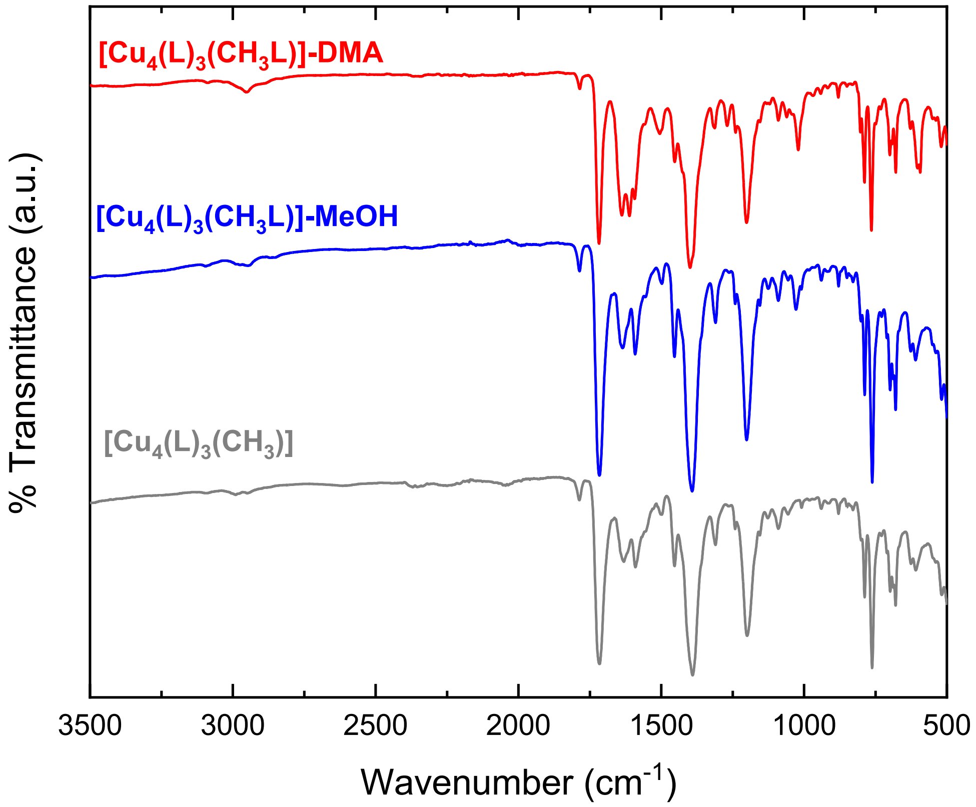

Figure S92. Infra-red spectra of bulk **$[Cu_4(L)_3(CH_3L)]$-DMA** (red), after MeOH exchange **$[Cu_4(L)_3(CH_3L)]$-MeOH** (blue), and after gas sorption analysis in **$[Cu_4(L)_3(CH_3L)]$** (grey).

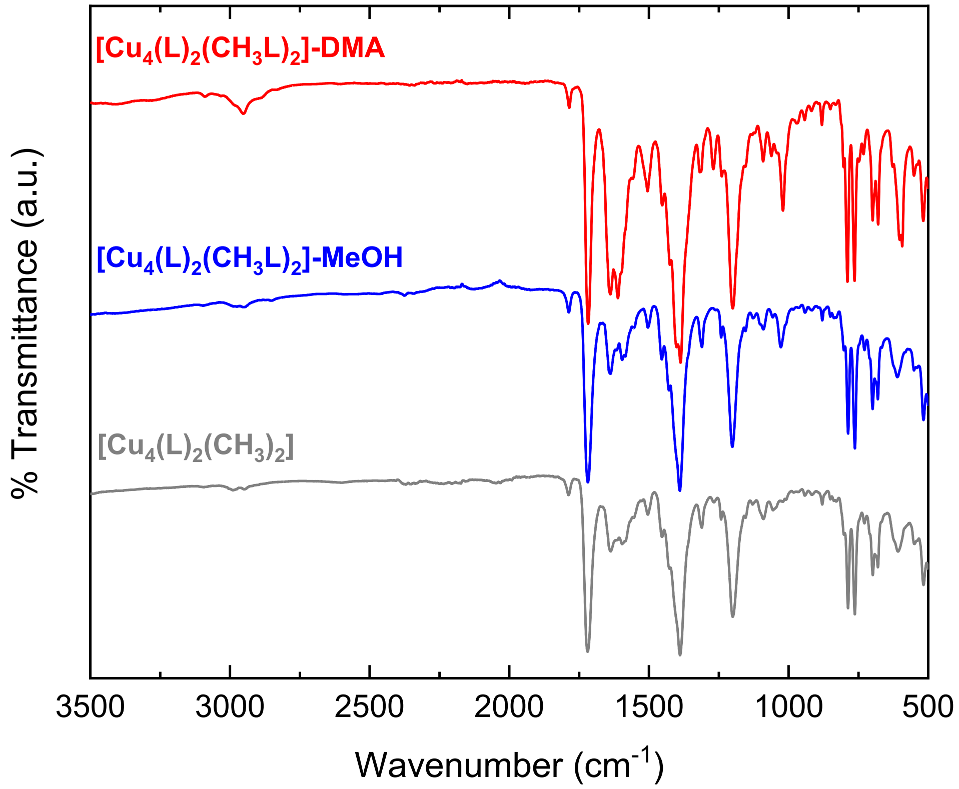

Figure S93. Infra-red spectra of bulk **[$Cu_4(L)_2(CH_3L)_2$]-DMA** (red), after MeOH exchange **[$Cu_4(L)_2(CH_3L)_2$]-MeOH** (blue), and after gas sorption analysis in **[$Cu_4(L)_2(CH_3L)_2$]** (grey).

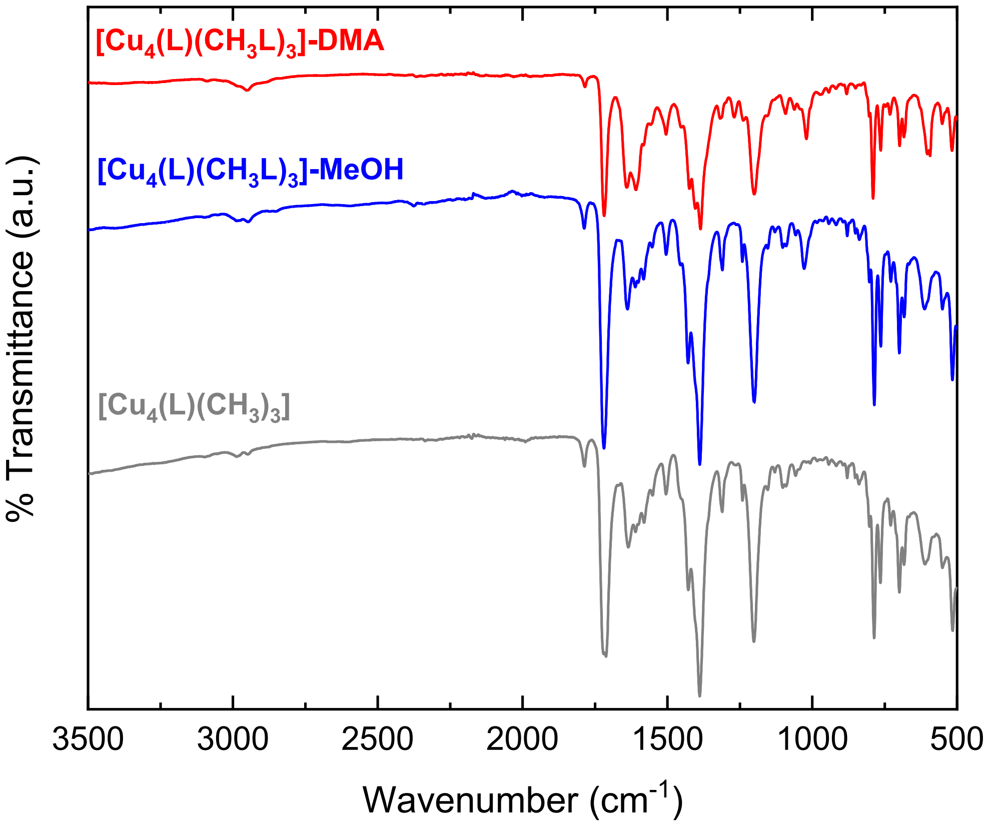

Figure S94. Infra-red spectra of bulk **$[Cu_4(L)(CH_3L)_3]$-DMA** (red), after MeOH exchange **$[Cu_4(L)(CH_3L)_3]$-MeOH** (blue), and after gas sorption analysis in **$[Cu_4(L)(CH_3L)_3]$** (grey).

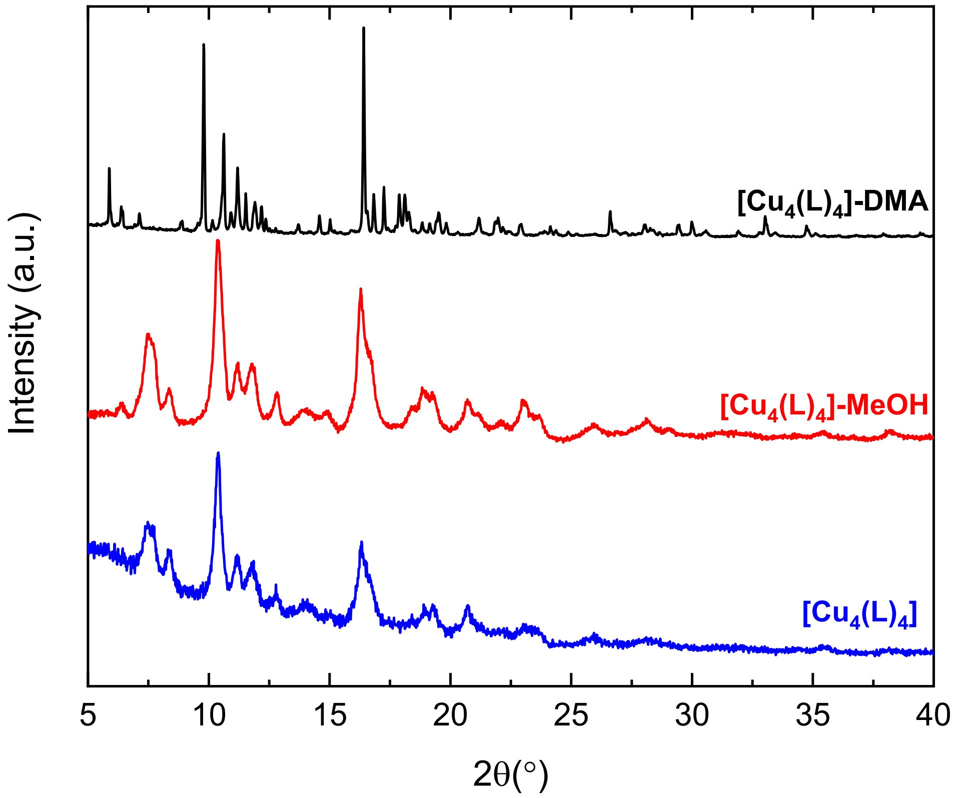

Figure S95. Powder X-ray diffractograms of bulk **[Cu_4(L)$_4$]-DMA** (black), after MeOH exchange **[Cu_4(L)$_4$]-MeOH** (red), and after gas sorption analysis in **[Cu_4(L)$_4$]** (blue).

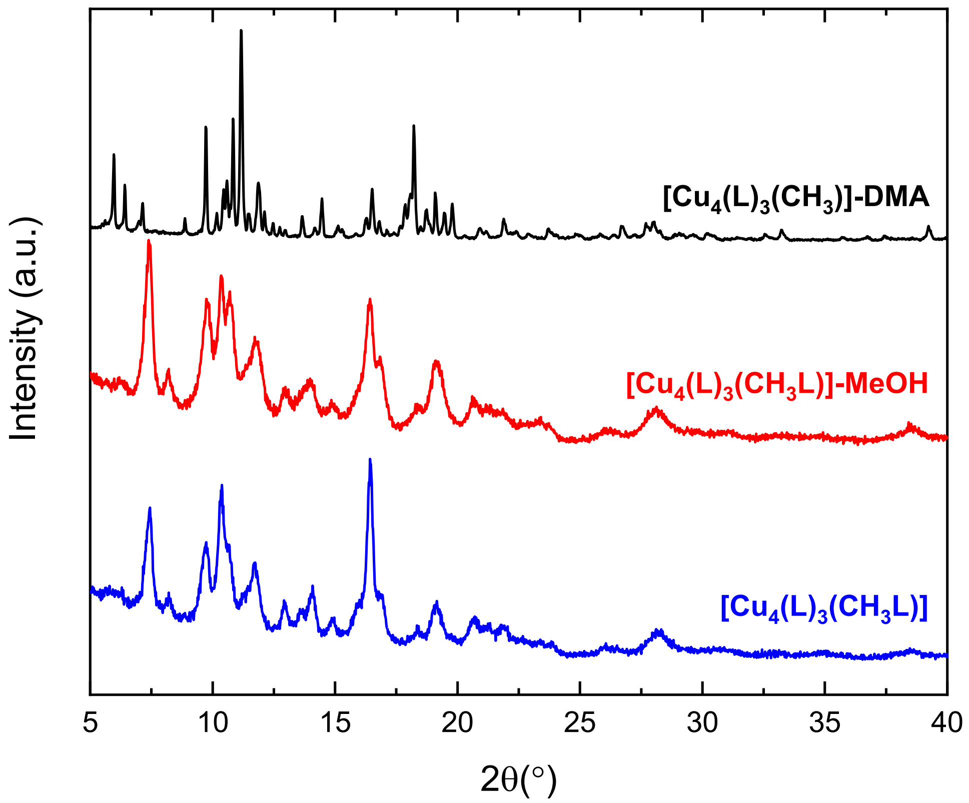

Figure S96. Powder X-ray diffractograms of bulk **[Cu_4(L)$_3$(CH_3L)]-DMA** (black), after MeOH exchange **[Cu_4(L)$_3$(CH_3L)]-MeOH** (red), and after gas sorption analysis in **[Cu_4(L)$_3$(CH_3L)]** (blue).

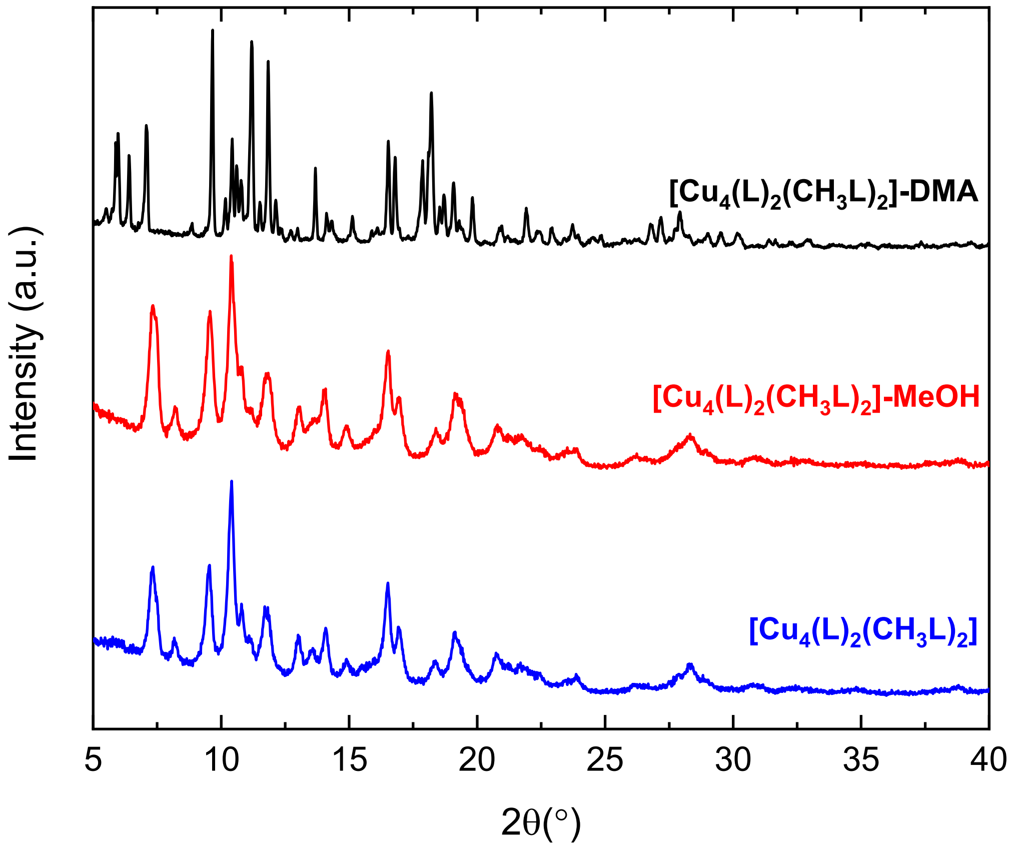

Figure S97. Powder X-ray diffractograms of bulk **[$Cu_4(L)_2(CH_3L)_2$]-DMA** (black), after MeOH exchange **[$Cu_4(L)_2(CH_3L)_2$]-MeOH** (red), and after gas sorption analysis in **[$Cu_4(L)_2(CH_3L)_2$]** (blue).

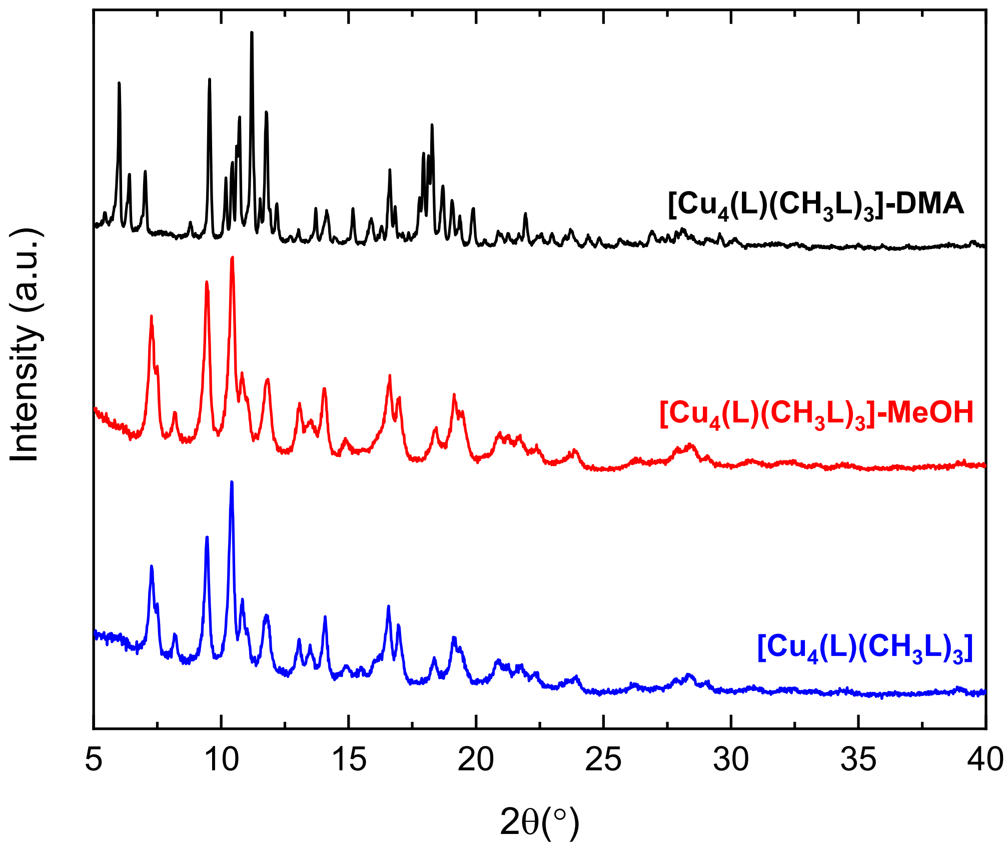

Figure S98. Powder X-ray diffractograms of **[Cu$_4$(L)(CH$_3$L)$_3$]-DMA** (black), after MeOH exchange **[Cu$_4$(L)(CH$_3$L)$_3$]-MeOH** (red), and after gas sorption analysis in **[Cu$_4$(L)(CH$_3$L)$_3$]** (blue).

Single crystal X-ray diffraction

General. All measurements were made with a Rigaku Synergy-i instrument. Structures were refined to convergence against F^2 using all independent reflections. Full structural details, including structure factors, have been deposited with the CCDC as deposition numbers 2303899 to 2303902. For **$[Cu_4(L)_4]$-DMA**. All the non-hydrogen atoms present in the structure were refined anisotropically, including the coordinated solvent molecules and the solvent molecules present in the void. Due to disorder, the benzoic ring containing the atoms C23, C24, C25 and were split into two different positions (labelled A and B) with a relative occupancy of 0.57:0.43 due to disorder. Coordinated DMA molecule containing the atoms O100, O1D, C100, C1D, C101, C2D, C102, C3D, C103, C4D, N100, N1D was split into two positions with a relative occupancy of 0.74:0.26. Finally, DMA solvent molecule present in the void containing the atoms N201, O200, O300, C201, C202, C203, C204, N300, O300, C301, C302, C303 and C304 were split in two different positions with a relative occupancy of 0.63:0.37. Due to the high disorder present in the void of the cage related with solvent DMA molecules, SQUEEZE[1] algorithm in PLATON[2] was used to account the electron density. Only a single void was found with a volume of 3168 Å^3 containing 931 electrons per unit cell (or 466 electrons per MOP). This accounts approximately for 9 DMA molecules (432 electrons) and 3 water molecule (30 electrons) per MOP. Here and in the scrambled structures below, appropriate restraints and constraints were applied to disordered groups so as to ensure that geometric parameters and displacement ellipsoids approximated to normal behaviour.

For **$[Cu_4(L)_3(CH_3L)]$-DMA** all non hydrogen atoms forming the skeleton of the cage, including coordinated solvent molecules were refined anisotropically with the exception of methyl groups formed by C1M and C2M and benzoic ring formed by the atoms C2, C3, C4, C5, C6, C7, C2A, C3A, C4, C5A, C6A and C7A due to disorder. The atoms of this disordered ring were modelled as split between two different positions with an occupancy of 0.64:0.36. As the crystal correspond with a scrambled material, the atoms of the methyl groups of the methylated ligands refined to occupancies of approximately 0.20 for C1M and C2M and 0.30 for C3M and C4M. All DMA solvent molecules in the void of the cage were refined isotropically due to high disorder. DMA molecule in the void of the cage containing the atoms N2S, N2T, N3S, N3T, C5S, C5T, C6S, C6T, C7S, C7T, C8S, C8T was modelled in two positions with a relative occupancy of 0.56:0.44. DMA molecule containing atoms N3S, N3T, O4S, O4T, C9S, C9T, C10S, C10T, C11S, C11T, C12S, C12T was modelled in two

different positions with an occupancy of 0.51:0.49. DMA molecule containing atoms N4S, N4T, O5S, O5T, C13S, C13T, C14S, C14T, C15S, C15T, C16S, C16T was modelled in two different positions with an occupancy of 0.51:0.49. DMA molecule containing atoms N5S, N5T, O6S, O6T, C17S, C17T, C18S, C18T, C19S, C19T, C20S, C20T was modelled in two different positions with an occupancy of 0.67:0.33.

For **$[Cu_4(L)_2(CH_3L)_2]$-DMA** all non-hydrogen atoms forming the skeleton of the cage, including the coordinated solvent molecules in the structure were refined anisotropically with the exception of benzoic ring containing the atoms C2, C3, C4, C5, C6, C7, C2A, C3A, C4A, C5A, C6A and C7A that were modelled isotropically due to disorder. Due to scrambling, the methyl groups occupancies were refined to approximately 0.52 for C1M and C2M and 0.64 for C3M and C4M. All DMA solvent molecules present in the void of the cage were refined isotropically due to disorder. DMA molecule containing the atoms N2S, N2T, N3S, N3T, C5S, C5T, C6S, C6T, C7S, C7T, C8S, C8T was modelled in two positions with a relative occupancy of 0.52:0.48. DMA molecule containing atoms N3S, N3T, O4S, O4T, C9S, C9T, C10S, C10T, C11S, C11T, C12S, C12T was modelled in two different positions with an occupancy of 0.55:0.45. DMA molecule containing atoms N4S, N4T, O5S, O5T, C13S, C13T, C14S, C14T, C15S, C15T, C16S, C16T was modelled in two different positions with an occupancy of 0.54:0.46. DMA molecule containing atoms N5S, N5T, O6S, O6T, C17S, C17T, C18S, C18T, C19S, C19T, C20S, C20T was modelled in two different positions with an occupancy of 0.58:0.42.

For **$[Cu_4(L)(CH_3L)_3]$-DMA** all non-hydrogen atoms forming the skeleton of the MOP were refined anisotropically, with the exception of the disordered benzoic ring formed by the atoms C2, C3, C4, C5, C6, C7, C2A C3A, C4A, C5A, C6A and C7A due of disorder. This benzoic ring was modelled over two positions with a relative occupancy of 0.67:0.33. Due to the scrambled character of the structure, the atoms of the methyl groups of one ligand type have been modelled with refined occupancies of approximately 0.67 for C1M and C2M and 0.89 for C3M and C4M. Moreover, DMA solvent molecules coordinated to the outer site of the copper paddlewheels were refined anisotroically. Water molecules, coordinated to the inner part of the paddlewheel was refined isotropically due to disorder and split in two different position with a relative occupancy of 0.5:0.5. Solvent DMA molecules in the void of the cage were refined isotropically due to high disorder. The DMA molecule in the void space containing the atoms N3S, N3T, C9S, C9T, C10S, C10T, C11S, C11T,

C12S, C12T, O4S and O4T were split in two different positions with an occupancy of approximately 0.5:0.5.

References

1. A. L. Spek, *Acta Crystallogr.*, 2015, **C71**, 9-18.
2. A. L. Spek, *Acta Crystallogr.*, 2009, **D65**, 148-155.
